# Enhancing sleep quality for nursing home residents with dementia: a pragmatic randomized controlled trial of an evidence-based frontline huddling program

**DOI:** 10.1186/s12877-021-02189-8

**Published:** 2021-04-27

**Authors:** A. Lynn Snow, Julia Loup, Robert O. Morgan, Kathy Richards, Patricia A. Parmelee, Rosa R. Baier, Ellen McCreedy, Barbara Frank, Cathie Brady, Liam Fry, Megan McCullough, Christine W. Hartmann

**Affiliations:** 1 Alabama Research Institute on Aging and the Department of Psychology, The University of Alabama, Gordon Palmer Hall, Tuscaloosa, AL 35487 USA; 2 Tuscaloosa Veterans Affairs Medical Center, Tuscaloosa, AL 35404 USA; 3 Department of Management, Policy and Community Health, School of Public Health, The University of Texas Health Science Center at Houston, Houston, USA; 4 School of Nursing, The University of Texas at Austin, Austin, TX 78701-1412 USA; 5 Brown University School of Public Health, Providence, RI 02912 USA; 6 B&F Consulting, Warren, RI 02885 USA; 7 Department of Internal Medicine, Dell Medical School, The University of Texas at Austin, Austin, TX 78712 USA; 8 Department of Public Health, Zuckerberg College of Health Sciences, University of Massachusetts Lowell, Lowell, MA 01854 USA; 9 Center for Healthcare Organization and Implementation Research, VA Bedford Healthcare System, Bedford, MA 01730 USA

**Keywords:** Sleep, Alzheimer’s disease, Dementia, Quality improvement, Quality indicators, Health care, Program development, Front line staff, Nursing homes, Long term care, Mental health

## Abstract

**Background:**

Disturbed sleep places older adults at higher risk for frailty, morbidity, and even mortality. Yet, nursing home routines frequently disturb residents’ sleep through use of noise, light, or efforts to reduce incontinence. Nursing home residents with Alzheimer’s disease and or related dementias—almost two-thirds of long-stay nursing home residents—are likely to be particularly affected by sleep disturbance. Addressing these issues, this study protocol implements an evidence-based intervention to improve sleep: a nursing home frontline staff huddling program known as LOCK. The LOCK program is derived from evidence supporting strengths-based learning, systematic observation, relationship-based teamwork, and efficiency.

**Methods:**

This study protocol outlines a NIH Stage III, real-world hybrid efficacy-effectiveness pragmatic trial of the LOCK sleep intervention. Over two phases, in a total of 27 non-VA nursing homes from 3 corporations, the study will (1) refine the LOCK program to focus on sleep for residents with dementia, (2) test the impact of the LOCK sleep intervention for nursing home residents with dementia, and (3) evaluate the intervention’s sustainability. Phase 1 (1 year; *n* = 3 nursing homes; 1 per corporation) will refine the intervention and train-the-trainer protocol and pilot-tests all study methods. Phase 2 (4 years; *n* = 24 nursing homes; 8 per corporation) will use the refined intervention to conduct a wedge-design randomized, controlled, clinical trial. Phase 2 results will measure the LOCK sleep intervention’s impact on sleep (primary outcome) and on psychotropic medication use, pain and analgesic medication use, and activities of daily living decline (secondary outcomes). Findings will point to inter-facility variation in the program’s implementation and sustainability.

**Discussion:**

This is the first study to our knowledge that applies a dementia sleep intervention to systematically address known barriers to nursing home quality improvement efforts. This innovative study has future potential to address clinical issues beyond sleep (safety, infection control) and expand to other settings (assisted living, inpatient mental health). The study’s strong team, careful consideration of design challenges, and resulting rigorous, pragmatic approach will ensure success of this promising intervention for nursing home residents with dementia.

**Trial registration:**

NCT04533815, ClinicalTrials.gov, August 20, 2020.

**Supplementary Information:**

The online version contains supplementary material available at 10.1186/s12877-021-02189-8.

## Background

A layman’s case for the benefits of a good night’s sleep is easy to make: imagine someone woke you last night *every two hours*. How would you feel? Now imagine that routine continues every night for the next month. For many residents of nursing homes, such awakening is standard practice. Nursing homes implement such segmented sleeping routines to address various care challenges (e.g., incontinence), even though evidence from both objective and subjective measures identifies disturbed sleep as a key contributor to many types of physical, emotional, and cognitive decline [1–4], including risk for frailty, morbidity, and even mortality [5–10]. Individuals with Alzheimer’s disease and related dementias (hereafter referred to as ‘dementia’)—almost two-thirds of long-stay nursing home residents [11] — are particularly affected by sleep disturbance [12, 13]. Poor sleep quality in this population is associated with increases in self-reported fatigue, difficulties with activities of daily living (ADLs), depression, fall risk, and, together with decreases in memory, mobility, morbidity, and even rates of survival [3, 14–17].

Various factors create suboptimal sleeping conditions for residents in nursing homes, including efforts to reduce incontinence [18], environmental disturbances such as frequent noise and light [19–21], and residents spending much of the day without engaging in physical activity [22, 23]. Nursing home residents with dementia are more likely to receive antipsychotic or antidepressant medications compared to other residents [24]; these medications may worsen nighttime sleep [25], In addition, withdrawal from antipsychotic medication may temporarily worsen sleep [26]. Pain also may affect sleep through nighttime disturbance and reduced physical functioning during the day [27]. The relationship between sleep quality and physical functioning (e.g., activities of daily living) is also well-established, with poorer objective sleep associated with poorer physical functioning in older adults [3, 6, 17, 28] and those with dementia [29].

Despite the common knowledge that sleep disturbance has negative impacts on nursing home residents, and despite the availability of easy ways to measure (e.g., actigraphy tools) and intervene to increase sleep quality, little has been done to improve the situation. A 2018 systematic review of nonpharmacological sleep interventions in nursing homes indicated promise for system-level environmental changes such as increased daytime light exposure, nighttime use of melatonin, and acupressure [30]. But another 2018 review noted that sleep disturbance remains extremely common for nursing home residents [31]. Even when nursing homes do recognize the need for systems-level changes and act to make those changes, their efforts are often translated into a one-size-fits-all approach to implementation, ultimately overlooking the complex interplay between individual residents’ needs, staff availability, and environmental barriers [32–34]. Because good sleep results from a complex interplay of resident, staff-generated, and environmental factors [32], sleep regimens for nursing home residents work best when *individualized* instead of applied generically.

In response, this study aims to improve awareness and measurement of sleep through implementation of a sleep-focused, frontline staff huddling intervention, systematic measurements of individual nursing home residents’ sleep, and targeted intervention sustainment through follow up visits (which may include virtual visits) with participating nursing homes.

### Huddling and the LOCK program

Quality improvement (QI) barriers such as staffing problems and top-down approaches can hamper nursing homes’ efforts to enhance care quality [35, 36]. QI studies in nursing homes have responded to these barriers by underscoring the importance of open communication and relationship building to improve resident clinical outcomes [35, 37, 38]. A recent systematic review highlights key components for successful nursing home QI: changing staff behavior, targeting specific care tasks, and using intervention theories [39]. One QI practice that incorporates these components, and therefore has potential to meaningfully impact resident sleep, is frontline staff huddling. This type of huddling involves brief, stand-up meetings that facilitate efficient, collaborative information exchange about specific topics, concerns, or resident needs. Frontline staff huddling promotes communication across clinical roles [40–42] and improvements in clinical outcomes across residents [43–45]. More broadly, consistent huddling can help improve quality of care and sustain changes individually and systematically by directly involving frontline health care workers [46].

**Fig. 1:**
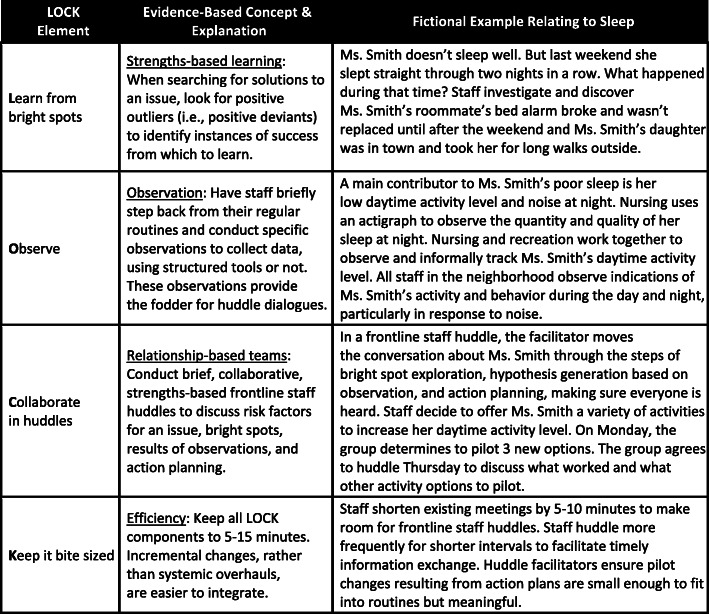
LOCK elements, corresponding evidence-based concepts, and examples

In a prior series of studies [47, 48], we incorporated the evidence-based practices of strengths-based learning [49, 50], systematic observation [51], efficiency [48, 52], and relationship-based teamwork [53, 54] into a specific frontline staff huddling program known as the LOCK program. The LOCK program enables staff to (1) Learn from bright spots (focus on positive evidence of strengths); (2) Observe (collect data through systematic observation); (3) Collaborate in huddles (conduct frontline staff huddles); and (4) Keep it bite-size (limit activities to 5–15 min for efficiency) [48, 55]. (See Fig. 1; LOCK Elements, Corresponding Evidence-Based Concepts, and Examples). The LOCK program actively guides frontline staff through a process of addressing particular resident outcomes of concern—in the case of this proposal, sleep—using frontline staff huddling on a daily, weekly, or other applicable consistent schedule as the foundation. Beyond the focused impact on residents’ sleep, our use of the LOCK program aims to increase nursing home staff application and use of the LOCK huddling method.

A pilot LOCK program targeting staff-resident interactions in 6 Veterans Health Administration (VA) nursing homes resulted in meaningful quantitative and qualitative improvements [47]. The 6 participating nursing homes showed significant pre- and post-intervention improvements in interpersonal interactions with residents for whom staff were providing care and reductions in the quantity of negative staff interactions with residents. The LOCK program was subsequently rolled out to all 134 VA nursing homes using a train-the-trainer approach. With the support of an intervention team that included national experts in nursing home QI implementation, geriatric sleep, and clinical dementia interventions, the rollout of the LOCK program expanded to focus on a variety of resident clinical conditions such as pain and pressure ulcers. The LOCK program’s methods have also improved clinical care in several non-VA nursing homes, including those with a history of serious, intransigent quality issues [46, 56, 57].

The combined results of the series of preliminary work with the LOCK program (1) enabled our team to standardize the LOCK program, (2) resulted in qualitative data on best practices for LOCK program implementation in nursing homes, and (3) generated implementation materials, all of which we will use for the present study to systematically measure sleep outcomes data in the context of our targeted LOCK sleep program.

#### Intervention

This clinical trial is a pragmatic study with two consecutive phases of refining and implementing the LOCK sleep intervention (see Additional Document 1: Manual of Procedures). Phase 1 consists of the following milestones over a period of one year: identifying pilot sites, initiating and completing intervention training, testing primary data collection, and transferring data. Upon successful achievement of these milestones, a four-year Phase 2 will test the impact of implementing the LOCK sleep intervention on nursing home residents’ sleep outcomes. Phase 2 will implement a pragmatic trial in 3 multi-facility, community-based, nursing home corporations caring for residents with dementia. In collaboration with those corporations, we will conduct a three-wave wedge-based, cluster randomized, controlled trial (RCT) with 24 nursing homes.

Our design enables testing of the extent to which the LOCK sleep intervention, after refinement for the nursing home resident dementia population, accomplishes the following:

1. Improves sleep quality as assessed by actigrapy (primary outcome)

2. Reduces psychotropic, hypnotic, and analgesic medication use (secondary outcome); and

3. Minimizes residents’ pain, mood, behavior symptoms, skin breakdown, and decline in activities of daily living (ADL) (secondary outcomes)

Results will additionally provide insight into variation of the intervention’s implementation and sustainability across care settings, nursing home organizational structure, and resident cases. Measurement of the implementation success of our LOCK sleep intervention will be guided by the Consolidated Framework for Implementation Research (CFIR) [58–60].

### Intervention strategies

The LOCK sleep intervention incorporates strategies known to be effective in healthcare organizations and for behavioral change at both a staff and resident level. To further support the its successful implementation, we built several strategies into the study design to efficiently target nursing home staff interaction, improve sleep outcomes for nursing home residents with dementia, and support sustainment: (1) frontline staff involvement, (2) staff training, (3) video dissemination, (4) novel technology testing, and (5) sustainment.

1. Frontline staff involvement

The LOCK sleep intervention explicitly supports frontline staff involvement through a structured frontline-inclusive staff huddling process that ensures collaborative and resident-individualized problem solving. The huddles will be regular short stand-up meetings occurring a few times each week and will include the medical interdisciplinary team, front-line staff, and any other staff who have resident relationships and knowledge (e.g., activities, custodial). Such direct staff involvement is central to achieving improvements in quality of care and naturally integrates open communication and connectivity into care teams’ daily dialogues [37, 38]. Inclusion at the frontline level allows for important information about resident’s needs, preferences, and habits to be incorporated in solutions, while also avoiding the more commonly utilized, and less successful, exclusively top-down QI strategies [35].

2. Staff training

During study Phase 1, we will train corporate coaches from each nursing home corporation on the LOCK sleep intervention. Coaches will be local nursing home employees primarily responsible for the provision of training and regular supervision and support for all site leadership teams. They will also provide oversight of the program at all sites during Phase 2, with the support and weekly supervision of our intervention consultants. Coaches will guide the leadership in each nursing home site by creating a study leadership/implementation team, training and mentoring those teams, and working with those teams to track data and reviewing progress to make midcourse corrections [45]. This train-the-trainer approach requires only minimal facilitation from the research team and consultants, thereby mimicking real-world conditions and enhancing the potential for future sustainment and dissemination of the intervention.

3. Video dissemination

We will create 10-min videos of nursing homes’ successful use of the LOCK sleep intervention over the course of Phase 1 and 2. The videos will be used during the study as education for fellow participating nursing homes and disseminated nationally after study completion.

4. Novel technology testing

After piloting of the LOCK sleep intervention, exploratory qualitative interviews with staff will investigate nursing home impressions of Fitbits in comparison to actigraphy. Fitbits (Fitbit Inc., San Francisco, CA) are an example of the large class of affordable and widely available consumer-level accelerometers, as contrasted with the class of medical-level accelerometers typically used by sleep laboratories (e.g., to collect our primary outcome sleep measurements we will use the Micro-Mini Motionlogger Actigraph by Ambulatory Monitoring Inc., Ardsley, NY). To our knowledge, no studies have examined nursing home staff-perceived benefits of such devices. Although anthropometry studies have demonstrated Fitbits to be inferior measurement devices for precise sleep research measurement purposes [61–63], they might be useful for real-world clinical intervention implementation because they are more financially accessible for nursing homes and allow staff to more easily and understandably interact with the residents’ sleep data in meaningful ways (e.g., user friendliness of design, multiple types of data, data visualization) [64].

5. Sustainment

We plan to investigate the LOCK sleep intervention’s sustainment in each nursing home six months after completion. Sustainment of an intervention—the extent to which it becomes part of usual care—is a little studied but significant area of implementation research [65–67]. During this follow up, we will conduct semi-structured interviews with participating nursing home staff to elucidate barriers to and facilitators of the intervention’s sustainment (or lack thereof).

## Methods

### Setting of the LOCK intervention

The LOCK sleep intervention will be implemented in a total of 27 nursing homes. We will recruit 3 nursing home corporations that have (1) a minimum of 12 nursing home sites (8 will eventually participate in the study), (2) 50+ beds, (3) ≥30 long-stay residents with dementia diagnoses, (4) centralized corporate training staff that can devote 50% time to implement the intervention (corporate coaches). Once the corporations are confirmed, eligible nursing homes within the corporations will be identified for recruitment of Phase 1 and 2. We will finalize nursing home eligibility criteria with the selected corporations using the nursing home’s Minimum Data Set (MDS) and Certification and Survey Provider Enhanced Reporting (CASPER) self-reported data as the trial progresses (see Data Collection for further details). Leaders at each participating site (i.e., administrator, chief nurse, medical director, nurse managers) will commit to (a) researcher-determined scheduling of intervention participation as per the randomization schedule, (b) taking on identity and responsibility of site leadership team, and (c) work with the study staff to execute secure data transfer/shipping protocols. Working with corporations provides the advantage of shared corporate infrastructure across nursing homes to facilitate screening, implementation, and data collection.

Nursing home recruitment for Phase 1 will require cooperation from the 3 enlisted corporations to identify 3 individual nursing homes (1 per corporation) to participate in the piloting and refining of the LOCK sleep intervention. Once identified, the study intervention team (study co-investigators and consultants with clinical and implementation expertise with the LOCK sleep intervention) will lead the Phase 1 implementation of the intervention. Stakeholder engagement will occur at both corporate and the nursing home levels during this phase. At the corporate level, each corporation will assign a corporate coach to attend all nursing home visits (which may be virtual visits) with the implementation team at their corporation’s selected Phase 1 nursing home. This allows training of corporate coaches to ensure preparedness and organizational support for the nursing home when they enter study Phase 2. At the nursing home level, each nursing home will create site leadership and implementation teams to support involvement of all staff in the LOCK sleep intervention.

Nursing home recruitment for Phase 2 includes expanding our sample to an additional 24 nursing homes, 8 from each corporation, using the same inclusion criteria as Phase 1. Once selected, each nursing home will receive the piloted and refined LOCK sleep intervention.

### Resident sample

In Phase 1, we anticipate enrolling 57 residents with dementia who meet inclusion/exclusion criteria (approximately 19 per each of the 3 nursing homes). In Phase 2, we anticipate enrolling 456 residents with dementia (approximately 19 per each of the 24 nursing homes). An approximation of 19 residents per nursing home is calculated according to an averaged 50% prevalence of dementia within nursing homes and conservative 50% consent rate based on our study team’s experience with previous nursing home dementia trials [68, 69]. We have included an estimate of approximately 20% attrition across the two study phases to maintain statistical power [70].

The potential participant pool from each selected nursing home will include all residents aged > = 50 years with a dementia diagnosis. Because the LOCK sleep intervention allows staff to focus on residents with dementia who have the greatest sleep problems, without differentiating by dementia severity, we will include residents across the range of dementia severity. Nursing home staff will be trained to identify nursing home residents with high risk of obstructive sleep apnea using the STOP-Bang screening tool [71–73], as well as trained on appropriate procedures for referring any positively screened residents for medical evaluation. We will exclude residents with a high risk of obstructive sleep apnea who are not yet being treated for said condition due to evidence of inaccurate actigraph measurements in that population [74]. Staff will also exclude residents who have a persistent bilateral resting tremor or paralysis in both arms (a subset of persons with Parkinson’s disease and related significant tremor-causing diagnoses), due to similar actigraph measurement inaccuracies [75].

Due to inclusion of residents with dementia diagnoses, a two-step resident consent process will be used to address the ethical balance between beneficence, assuring that residents who do not have the capacity to consent are protected by their legal authorized representative’s oversight, and promotion of autonomy for residents who do have capacity to consent. First, after leadership teams from each nursing home compiles a list of all residents with a dementia diagnosis, they will send a letter to each resident’s legally authorized representative informing them of the study and inviting the legally authorized representative to opt out if they do not wish to be contacted by research staff [76–79]. Research staff will then contact all representatives who do not opt out, inviting each to consent to the resident’s participation. Once legally authorized representative consent is collected, the second consent stage for nursing home residents with mild dementia will occur. Site leadership team members will work with our study team to identify those residents with a mild MDS Brief Interview for Mental Status (BIMS) score (i.e., a score above 10) and a resulting increased likelihood of capacity to consent [80]. Nursing home leadership teams will then arrange a telephone interview between study staff and these residents to ascertain the resident’s ability to consent using comprehension questions. If the resident is determined to have the ability to consent during this interview, the resident consent process formally commences at that time, with a nursing home staff person serving as an in-person witness.

Importantly, if a resident can respond appropriately to all capacity screening questions but does not wish to participate, they will not be enrolled in the study, regardless of their legally authorized representative’s consent. For nursing home residents without capacity to consent that become enrolled in the study, their assent to participate will be monitored during the study at each step, and their decision to withdraw at any time (whether expressed verbally or by resistance to participation) will be honored.

### Staff sample

We will enroll 60 frontline staff in the Phase 1, piloting and refinement year, of our clinical trial. In Phase 2, we will enroll 480 staff in our clinical trial across the 24 nursing homes. We will invite all frontline NH staff roles interested in the training and intervention to enroll (e.g., Nursing Assistants, Licensed Practical Nurses, Registered Nurses, and other interdisciplinary frontline staff members). We will recruit 20 staff per nursing home to participate in study interviews about their experiences with the LOCK sleep intervention and Fitbits at mid- and at six-months post-implementation.

To increase enrollment and retention of staff, we will communicate the compelling, evidence-based benefits of the LOCK sleep intervention on improved work environments and quality of care for nursing home residents. The regular phone mentoring of nursing home corporate coaches and leadership teams will also facilitate staff retention and intervention fidelity. Consent is not required from staff members directly participating in the LOCK sleep intervention as the program itself consists of clinical practices common in nursing home QI efforts that fall within the scope of staff duties. However, it will be made clear that participation is strictly voluntary and no adverse consequences to the person’s job status or any other adverse consequences will occur if the person declines to participate in the LOCK sleep intervention at their nursing home facility. At the end of Phase 1 and Phase 2, research staff will obtain consent from the sub-sample of nursing home staff participating in interviews.

### Measures, data collection, and analysis methodology

#### Study design

##### The LOCK sleep intervention

In Phase 1, the LOCK sleep intervention will initially use intervention materials (assistance for developing teams and implementing the intervention, checklists, role-play scenarios, and videos) developed from the pilot 6-site VA nursing home implementation [33]. Throughout this phase, we will make additional refinements based on our intervention team’s experiences from their previous work. We will also carefully adapt our materials to the non-VA nursing home setting, which differs in terms of resident population (VA is mostly male), staffing (VA has higher professional nurse staffing ratios), and leadership (VA medical director is usually on site).

**Fig. 2:**
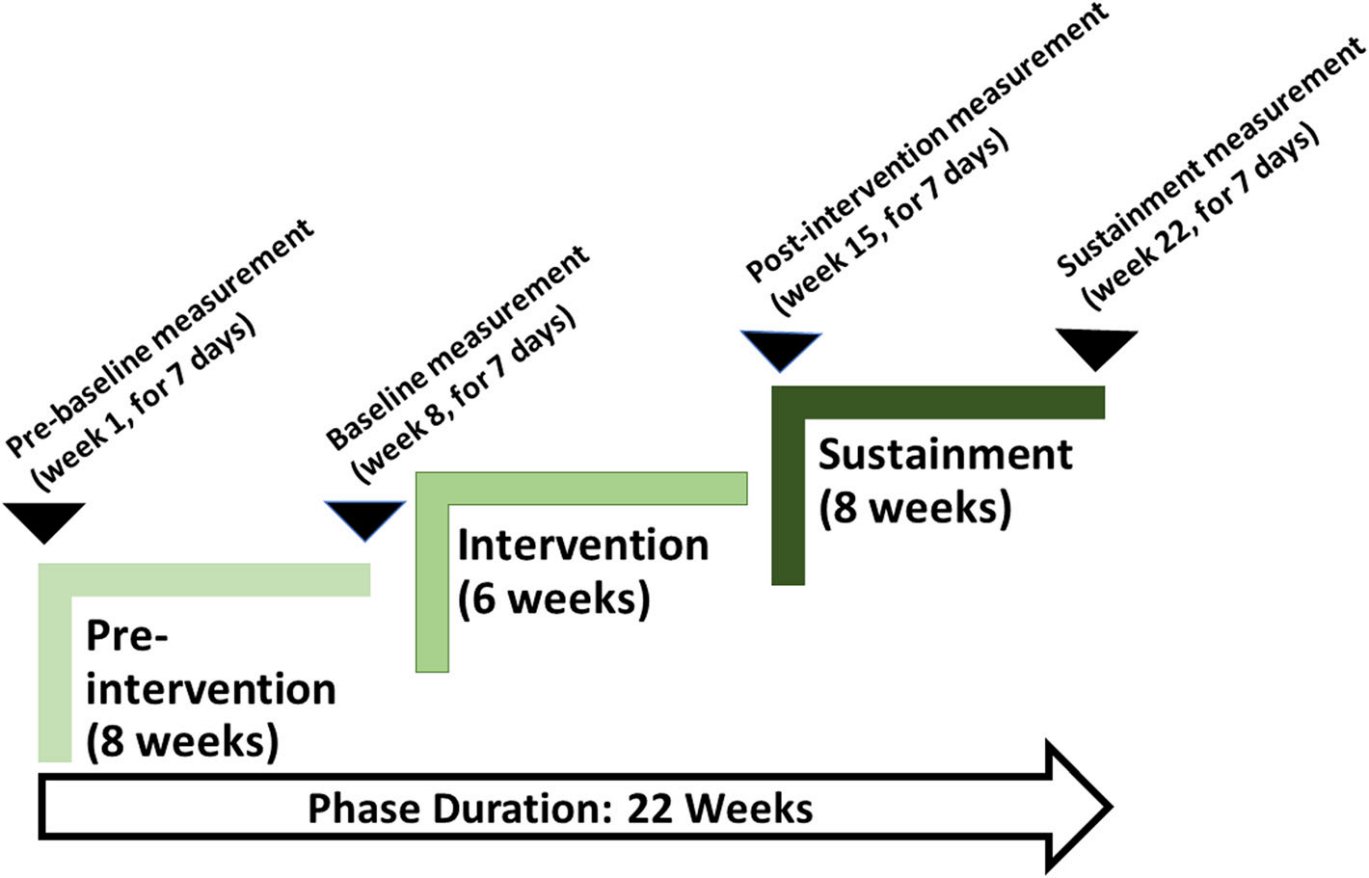
Timing and spacing of intervention implementation and sleep outcomes measures

Phase 2 will test the refined LOCK sleep intervention through an incomplete, stepped, wedge cluster randomized controlled trial (RCT) design [81] with 24 additional nursing homes, each acting as its own control (6 staggered steps, with 4 nursing homes per step) (See Table 1: Stepped Wedge Design with Measurement Periods). During Phase 2, each cluster of 4 nursing homes will participate in its own 22-week intervention cycle (see Fig. 2: Timing and Spacing of Intervention Implementation and Sleep Outcomes Measures) after being assigned first to the control condition and then phased into the intervention at 7-week intervals. We will randomize nursing homes within corporations to steps after matching on bed size and number of long-stay residents with dementia diagnoses. Residents within each nursing home are followed longitudinally, i.e., from pre- through post-intervention measurement periods.

**Table 1:**
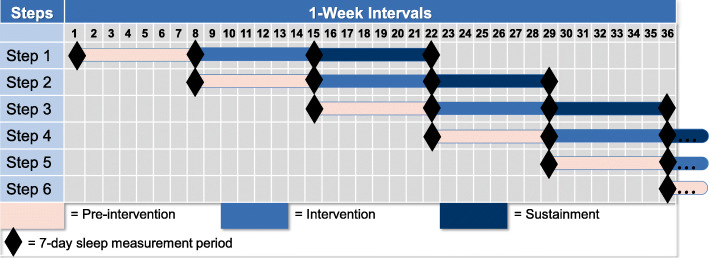
Stepped wedge design with measurement periods

##### Outcome measures

The primary outcome for the study is actigraph-measured resident sleep. Total sleep time will be measured using data from Micro-Mini Motionlogger Actigraphs worn by residents at night (Ambulatory Monitoring Inc., Ardsley, NY) [82]. We will define nighttime as 10 pm to 6 am and will compute total sleep time as the total number of minutes asleep during a nighttime period. To further evaluate the impact of the LOCK sleep intervention for resident sleep quality, we will also examine the following: wake after sleep onset (total number of minutes awake during nighttime), how often the resident awoke during the nighttime, sleep efficiency (the ratio of minutes asleep to minutes awake over the period), and sleep fragmentation (an index of restlessness computed as the percentage of one-minute epochs scored as awake).

Due to imperfect correlations between actigraphic sleep data and self- or other ratings of resident sleep [83, 84], four supplementary sleep measurements will be collected from staff to triangulate the primary sleep outcome data: (a) Sleep via staff rating: a clinical, global impression of resident sleep change at the end of each 7-night actigraph sleep measurement period and at the end of each week of the 6-week sleep intervention period [85–92]; (b) Staff-identified sleep-related concerns: Huddle facilitators lead the huddle team to identify and track up to 2 additional symptoms or behaviors of concern potentially related to sleep (e.g., nighttime agitation, pain), measured on the same schedule as the ‘sleep via staff rating’; (c) Inter-resident sleep variability: Medical record data to be collected at the end of the 22-week trial measurement period on the following: (1) changes in any sedating medications and/or dosages; (2) incidents of delirium; (3) any urinary tract infections; and (4) doses of any sedating medications, including as needed ones; (d) Sleep from the Minimum Data Set (MDS): Two MDS items pertaining to sleep will be included: (1) “trouble falling or staying asleep or sleeping too much.”; (2) “over the past 5 days, has pain made it hard for you to sleep at night?”

Our secondary outcomes will come from the nursing homes’ MDS 3.0. Every Medicare-certified nursing home is required to complete an MDS assessment for every resident at admission, quarterly, and at discharge, in addition to whenever the resident’s status changes. We will be using the MDS to collect details about the residents’: (1) psychotropic medication use in the last seven days; (2) self-reported pain index [93, 94]; (3) adherence to a regimen or isolated use of analgesic medication in the last five days, noting use of additional non-medication pain interventions; and (4) indication of decline in activities of daily living (ADLs) when considering past MDS assessments and overall functional ability [95–98]. (See Table 2: Primary, Secondary, and Other Outcome Measures).

**Table 2:** Primary, secondary, and other outcome measures

Category	Name	Time Frame	Brief Description
Primary	Total Sleep Time via Actigraph	Baseline, Intervention, Post-Treatment	Actigraph measurement of the total number of minutes the subject is asleep between 7 pm and 7 am.
Secondary	Psychotropic Medication Use	Baseline, Intervention, Post-Treatment	Psychotropic medication use as recorded in the Minimum Data Set (MDS)
Secondary	Pain and Analgesic Medication Use	Baseline, Intervention, Post-Treatment	Psychotropic medication use as recorded in the Minimum Data Set (MDS)
Secondary	Activities of Daily Living Decline	Baseline, Intervention, Post-Treatment	Activities of daily living as recorded in the Minimum Data Set (MDS)
Other	Sleep Staff Rating	Intervention, Post-Treatment	Staff rating of sleep global impression of change
Other	Staff-identified Sleep-related Concerns	Intervention, Post-Treatment	Staff rating of sleep-related concerns global impression of change
Other	Inter-resident Sleep Variability	Baseline, Intervention, Post-Treatment	NH medical record data to indicate (a) changes in any sedating medications and changes in dosages; (2) incidents of delirium; (3) any urinary tract infections; (4) doses of any sedating medications, including as needed ones
Other	Sleep Information from MDS	Baseline, Intervention, Post-Treatment	The MDS contains only two items pertaining to sleep. Because MDS is the foundational nursing home administrative quality data set, these items will be examined for their utility. One item, part of the PHQ-9, is, “trouble falling or staying asleep or sleeping too much.” The pain section also includes one item for residents who can self-report, “over the past 5 days, has pain made it hard for you to sleep at night” (there is not a comparable item in the MDS staff interview section for residents unable to self-report).

Semi-structured interviews will also provide the qualitative data needed for our team understand the fit and effectiveness of the LOCK sleep intervention during both Phases 1 and 2.

#### Data collection

Data collection for the primary sleep outcome measures will follow the same timeline in Phases 1 and 2, incorporating three measurement periods: pre-intervention, intervention, and sustainment. We anticipate an initial 8-week period in which each nursing home builds its frontline staff huddle practice across all units (pre-implementation period). In the first and last week of this period, nursing home staff will conduct a week (7 days) of continuous actigraph measurement on all enrolled residents in each nursing home. These will be the pre-baseline and baseline measurements. After this, each nursing home will begin the LOCK sleep intervention on all its units (implementation period), which involves no actigraph measurement. An 8-week sustainment period will follow. In the first and last weeks of the sustainment period, nursing home staff will also conduct a week (7 days) of continuous actigraph measurement on all enrolled residents in each nursing home. These will be the post-intervention and sustainment measurements. (See Fig. 2: Timing and Spacing of Intervention Implementation and Sleep Outcomes Measurement).

Nursing home staff will assist residents to wear both an actigraph and a Fitbit (if applicable during Phase 2) side by side on a wrist for the 7 days during each of the 4 measurement periods (weeks 1, 8, 15, and 22—see Fig. 2) [99–101]. As part of their intervention training, staff will be instructed on behavioral signs of distress. If a participant exhibits significant behavioral distress due to sleep measurement methods, staff will be instructed on modifications and alternatives. If the distress does not subside, staff will be instructed to discontinue the assessment. There will therefore be no alternative treatments due to the stepped-wedge, cluster randomized, controlled clinical trial design.

To record collected sleep and secondary data, the nursing home leadership team will use a researcher-provided, tracked, express mail service to send the research team all weekly completed staff rating forms. These mailed packages will also include any actigraphs and Fitbits for which the assessment periods are complete. For supplementary sleep data collection, research staff will additionally extract relevant information from each enrolled resident’s medical record.

For qualitative data collection, we will recruit a sample of nursing home staff engaged in huddles to participate in mid- and postimplementation semi-structured interviews (phone or in-person, as situations permit) to explore staff perceptions of the LOCK sleep intervention’s effectiveness, feasibility, facilitators, and challenges. During Phase 1, reviews of the Fitbits and suggestions to improve the interview process for Phase 2 (i.e., change in length, location, etc.) will also be collected.

#### Data management

The principal investigator will be responsible for ensuring participants’ safety on a daily basis. An independent Data and Safety Monitoring Board (DSMB) will meet at least once a year by teleconference (and more often as needed), acting in an advisory capacity to the NIA Director by (1) monitoring participant safety; (2) evaluating the progress of the trial; and (3) reviewing procedures for maintaining the confidentiality of data and the quality of data collection, management, and analyses. The DSMB meetings will be guided by the NIA’s DSMB Report Template, including but not limited to: study status and stopping guidelines, recruitment status, data quality status, interim results, and safety information. This study’s DSMB members have been approved by the trials’ assigned NIA program officer as independent investigators with no scientific, financial, or other conflict of interest with the study.

Personally identifiable information from the trial will be only be stored in a secure and securely backed up University of Alabama data server and University of Texas Health Data Center in password protected files. Other collaborating sites (Boston University, Brown University, and University of Texas at Austin) will only work with collected data identified by subject number. All data, and referenced personal information therein, will be monitored by the DSMB during and after the trial. HIPAA-compliant virtual visits will assist in streamlined data collection during reduced in-person access to nursing homes due to the COVID-19 pandemic.

#### Analysis

**Fig. 3:**
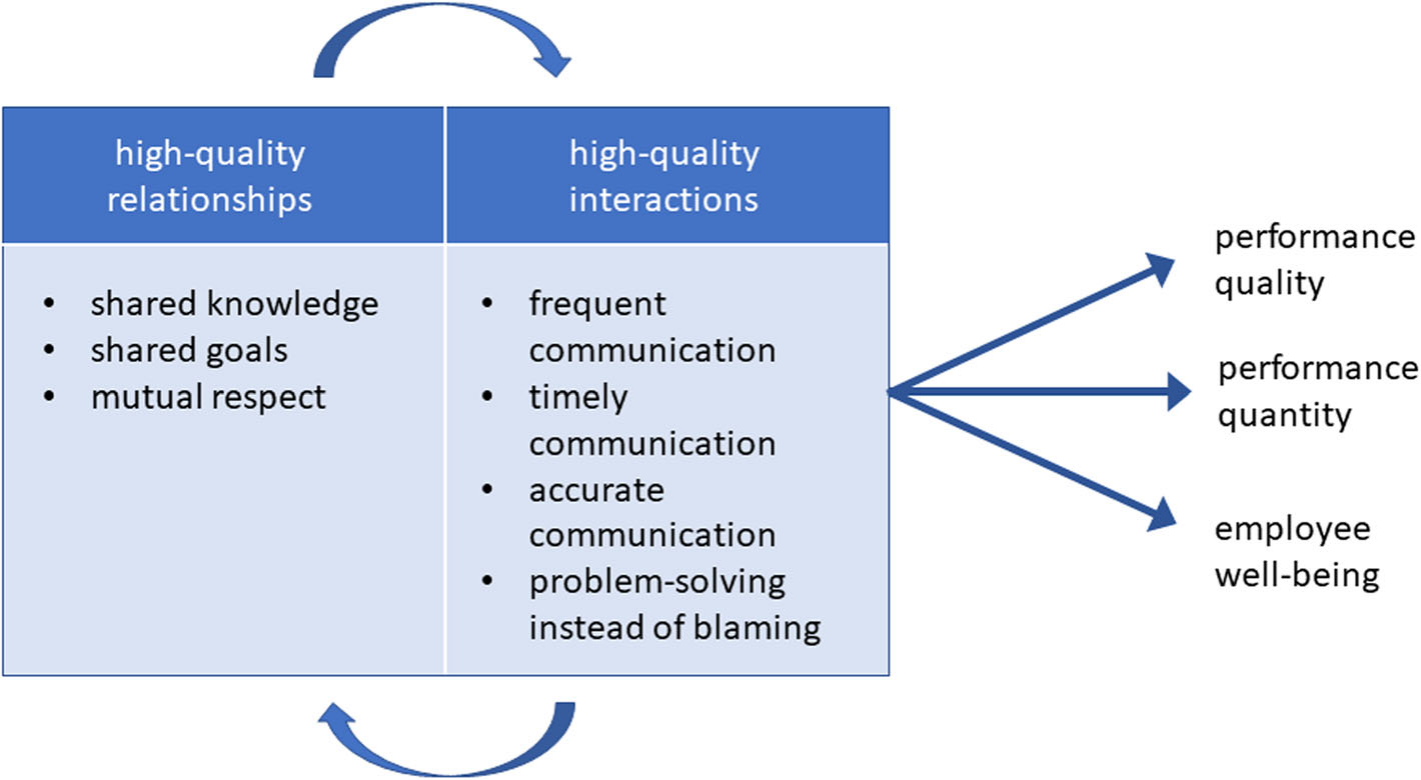
Relational Coordination Theory

In Phase 1 we will use standard microlongitudinal analysis methods to test the sensitivity of secondary outcomes and supplementary sleep data to individual differences and changes in residents’ total sleep time. For the qualitative interviews, all interviews will be audio recorded, transcribed, and processed through rapid appraisal template analysis [102, 103]. The Consolidated Framework for Implementation Research (CFIR) constructs, a widely used theory for evaluating intervention implementation [70, 104], and the Relational Coordination framework will form the foundations of the analysis template [105, 106] (See Fig. 3: Relational Coordination Theory). Results from this step will suggest if the value and feasibility of using the Fitbits in addition to the actigraphs supports their continued use in Phase 2.

After Phase 2 data collection is complete from all 24 nursing homes, we will examine differences in the primary outcome (total sleep time) across the pre-baseline through each nursing homes sustainment period (See Fig. 2). We will use the nursing home’s separate average total sleep times across each of the four measurements. We will use a multi-level mixed models approach and either generalized linear mixed models (GLMM) or generalized estimating equations (GEE). Both GLMM and GEE approaches enable the estimation of models with random and fixed effects and the use of outcome measures having the range of distribution types likely to be observed in primary and secondary outcomes [104].

The level of analysis will be the individual nursing home resident nested within nursing home corporations, with each nursing home serving as its own control and nursing homes stratified by corporation. Data available from the pre-baseline, baseline, postintervention, and sustainment measurements (See Fig. 2 and Table 1) will enable estimation of (a) within individual variation over time (pre-baseline [week 1] through sustainment [week 22]), (b) between individual variation incorporating individual characteristics (e.g., demographics, dementia severity level), and (c) facility level variation incorporating nursing home characteristics (e.g., corporate membership, facility bed-size, and staffing) [107, 108].

Since using actigraphs to measure sleep time will likely be a new practice in the nursing homes, the two pre-intervention measurement periods (pre-baseline [week 1] and baseline [week 8]) will enable us to examine the impact of measuring sleep time prior to the intervention separate from the effects of nursing home staff implementing the LOCK sleep intervention. The two post-implementation periods (post-intervention [week 15] and sustainment [week 22]) will enable us to test for longer-term intervention effects (sustainability).

Our primary analysis for trial evaluation will be a partial intention-to-treat analysis using total sleep time as the outcome. We will include all residents with both pre-baseline and baseline total sleep time assessments to properly estimate any effect of using the actigraph to measure sleep. To adjust for mid-trial attrition, we will use multiple imputation to estimate missing data, assuming no change from their last available total sleep time assessment and incorporating an estimate of random variation based on observed data. We will apply our model approach described above to the multiply-imputed datasets and results will be combined using Rubin’s Rule [109]. A complete case analysis (individuals with data from all measurement periods) will be conducted to test the sensitivity of our primary analysis results.

Participating nursing homes will be treated as random effects with residents clustered within nursing homes. By clustering within nursing home, we can control for site-level effects and plausibly treat the individual residents as independent [70]. We will estimate both unadjusted and adjusted models. We will include individual-level and nursing home-level characteristics in our adjusted models, as described above. A Type I error rate of 5% (α < .05) will be used to identify statistically significant associations. Raw and adjusted total sleep times will be reported for each period. We will use a similar approach for testing the impact of the LOCK sleep intervention on our secondary outcomes and our supplementary sleep data. For our secondary outcomes from the MDS, we will use data from each resident’s last available MDS assessment pre-intervention and the first available MDS post intervention. Additional MDS data may be included to extend the pre- and post-implementation assessment windows, increase our ability to distinguish individual variability from intervention-related change, and provide greater statistical power for these analyses. The form of the models tested will depend on the completeness of MDS data available for each participant.

We will triangulate the quantitative and qualitative data using thematic analysis according to responses associated with variation in the program’s implementation and sustainability [102].

#### Implementation assessment

For Phase 2, we will assess implementation success using CFIR [58, 59] according to the entire sample of 24 nursing homes. Our analysis will (1) characterize nursing home variation in implementation measures based on selected constructs within 4 CFIR domains [60] and (2) use multiple data sources to document and evaluate the implementation sustainment (Table 3: CFIR Implementation Constructs and Data Sources). In addition, we will use methods outlined by Keith et al. to create a measure of variability [110] using both qualitative and quantitative data. This measurement will reflect how the intervention was used in practice, how it was sustained, and identify influences for our results. The analysis methods will apply to both the post-intervention and the sustainment analyses. We will triangulate perspectives on the CFIR constructs, to the extent possible, using the different data sources. For the qualitative data, triangulation requires first using the taxonomy of themes to assign quantitative ratings and averaging them to create a ranking. For the quantitative measures, we will empirically test for correlation and create a rating by weighting the correlated measures and creating a summary ranking.

**Table 3:** CFIR implementation constructs and data sources

CFIR Domains & Constructs	Assessments
**Intervention Characteristics** - Relative Advantage [of intervention]	- Staff Interviews - Frequency of actigraph use per resident
**Process** - Planning - Engaging - Executing - Reflecting and Evaluating	- Staff interviews - Number of actigraphs used with residents - Amount of supplementary sleep data sent to researchers
**Inner setting [of NHs]** - Structural characteristics - Networks and Communications - Culture - Implementation climate	- Staff interviews - NH characteristics (payment mix, staffing size, chain and ownership status) from CASPER - Staff attendance at trainings and coaching calls - Leadership team turnover during intervention - Frequency of STOP-Bang use
**Outer Setting** - Peer Pressure [to implement]	- Staff interviews - Number of non-trial NHs within a corporation implementing the LOCK sleep intervention

Summary rankings will enable us to describe the variability in implementation among the trial’s nursing homes, both at the overall nursing home level and at the individual CFIR construct level. We will average the rankings across all constructs for each nursing home and then rank all nursing homes based on the average scores. This ranking process, crossed with staff impressions of the Fitbits, and rich qualitative data to provide context, will provide an overall summary of variability across nursing homes [111–113]. As a final step, we will examine how this study’s implementation metrics align with quantitative results for our primary, secondary, and supplementary sleep data outcomes, helping us understand how the nursing homes implemented the LOCK sleep intervention and how the program was sustained. This will inform expansion of the intervention as well as inform other nursing home QI initiatives (See Additional Document 2: Data Resource and Sharing Plan).

## Discussion

This trial’s rigorous evaluation of the LOCK sleep intervention will establish its effectiveness for improving sleep for nursing home residents with dementia and will characterize factors associated with effective intervention implementation and sustainment. Innovative, real-world studies like this are critical to assist nursing home staff in improving the quality of care for this vulnerable and growing population.

Our design and implementation plan are strengthened by an emphasis on implementation and intervention sustainment. Sustained use of the LOCK intervention will suggest the likelihood of its viable expansion within nursing homes to focus on other health and behavioral issues that nursing home staff and residents face, such as safety and infection control. It will also point to potential applications in settings other than nursing homes, such as assisted living and inpatient mental health units. In terms of other possible positive outcomes, the LOCK intervention’s emphasis on improved communication and teamwork may enable improved work experiences and therefore may lead to reduced staff turnover [114]. Successful implementation is also likely to benefit residents by facilitating better and earlier detection and treatment of sleep problems, as well as increasing staff’s attention to residents’ physical and mental health conditions.

Our innovative study also includes the use of a novel technology, the Fitbit, that has yet to be evaluated for use in nursing home sleep trial. Results will determine Fitbits’ potential value in terms of both design and access (financially, bodily) for this population and the nursing home setting.

Regarding study limitations, the emphasis on real-world implementation necessarily means some loosening of intervention implementation fidelity requirements. For example, depending upon the state of COVID-19 protection procedures during the course of the study, research staff may have little to no opportunity to visit participating nursing homes. In a pragmatic design, identification of specific active ingredients for change is not always possible, as is true in our case, where the intervention consists of several practices (sleep hygiene instruction, team huddling procedures, sleep measurement procedures).

## Conclusion

Our interdisciplinary team of researchers and consultants designed this study to improve clinical outcomes for nursing home residents with dementia. This study’s central innovation, the LOCK sleep intervention, holds promise for being an effective, real-world mechanism to improve sleep for nursing home residents and significantly move forward goals of the National Plan to Address Alzheimer’s Disease 2018 Update [115]. Upon implementation completion, this intervention has future potential to address other important issues faced by residents and expand to settings. Our strong research team, careful consideration of design challenges, and rigorous, pragmatic approach will work together to ensure success of this intervention. Efficacious implementation of our evidence-based intervention to improve residents’ sleep will significantly increase research understanding of how to implement and sustain nursing home interventions and improve quality of life and sleep for this important, growing, and vulnerable population.

## Supplementary Information

**Additional file 1.** Manual of Procedures. Includes description of training, personnel, intervention, measures, IRB-approved protocol, and informed consent documents.

**Additional file 2.** Data Resource and Sharing Plan.

## Data Availability

The datasets generated and/or analyzed during the current study are not publicly available because the current protocol has yet to begin. They will be available from the corresponding author on reasonable request after trial data collection has completed.

## Abbreviations

ADL: Activities of daily living
AHCA: American Health Care Association
CASPER: Certification and Survey Provider Enhanced Reporting
CFIR: Consolidated Framework for Implementation Research
DSMB: Data Safety Monitoring Board
GEE: Generalized estimating equations.
GLMM: Generalized linear mixed models
LOCK: “Learn from bright spots”, “Observe”, “Collaborate in huddles”, and “Keep it bite-size”
MDS: Minimum Data Set
NIA: National Institute on Aging
RCT: Randomized Controlled Trial
