# Supplementary material for: Enhancing sleep quality for nursing home residents with dementia: a pragmatic randomized controlled trial of an evidence-based frontline huddling program
Source: BMC Geriatr. 2021 Apr 27;21:281. doi: 10.1186/s12877-021-02189-8 (PMC8076882; doi:10.1186/s12877-021-02189-8)
Supplement: Supplementary file 1 — Additional file 1. Manual of Procedures. Includes description of training, personnel, intervention, measures, IRB-approved protocol, and informed consent documents. [file 12877_2021_2189_MOESM1_ESM.pdf]

Enhancing Sleep Quality for Nursing Home Residents with Dementia: Pragmatic Trial of an Evidence-Based Frontline Huddling Program (Acronym: 40Winks)

Manual of Operations and Procedures (MOP) for Multi-Site Clinical study

August 2020

Table of Contents

1.0 INTRODUCTION 4
2.0 OVERVIEW 4
3.0 MOP CONTENTS AND ORGANIZATION 4
4.0 ABSTRACT AND STUDY PROTOCOL 4
5.0 STUDY ORGANIZATION AND RESPONSIBILITIES 4
5.1 Roster 4
5.2 Coordinating Center 6
5.3 Study Sites 6
5.4 Steering Committee 7
5.5 Other Study Committees 8
5.6 NIA's Role and Responsibility 9
6.0 TRAINING PLAN 9
7.0 COMMUNICATIONS PLAN 12
8.0 STUDY FLOW 12
9.0 RECRUITMENT AND RETENTION 14
9.1 Screening and Eligibility Criteria 14
9.2 Screening Log 15
9.3 Eligibility Criteria 15
10.0 INFORMED CONSENT 15
10.1 HIPAA Authorization 15
11.0 STUDY INTERVENTION 16
12.0 RANDOMIZATION 16
13.0 BLINDING AND UNBLINDING (MASKING AND UNMASKING) 18
14.0 STUDY MEASUREMENTS AND PROCEDURES 19
14.1 Timeline and visit schedul*e* 19
14.2 Scope/Schema 23
14.3 Final Study/Early Discontinuation Evaluations 23
15.0 CONCOMITANT MEDICATIONS 23
16.0 SAFETY REPORTING 23
17.0 STUDY COMPLIANCE 23
18.0 DATA COLLECTION AND STUDY FORMS 29
18.1 Participant Binder 29

18.2 Study Forms 29
18.3 General Instructions for Completing Forms 30
18.4 Data Flow 30
18.5 Administrative Forms 30
18.6 Retention of Study Documentation 31

19.0 DATA MANAGEMENT 31
19.1 External Data 33
19.2 Quality Control Procedures 33
19.2.1 Standard Operating Procedures 34
19.2.2 Data and Form Checks 34
19.2.3 Site Monitoring 35

20.0 DATA AND SAFETY MONITORING ACTIVITIES 35
20.1 Reports 36
20.2 Study Completion and Close-Out Procedures 36
20.2.1 Participant Notification 36
20.2.2 Site Procedures 37
20.2.3 Confidentiality Procedures 37
20.2.4 Publications 40

21.0 MOP MAINTENANCE 40

BIBLIOGRAPHY 41

RELEVANT WEB SITES 45

APPENDIX A - ACRONYM GLOSSARY 46

APPENDIX B - SAMPLE SCREEN LOG 49

APPENDIX C - SAMPLE SCHEDULE OF EVENTS 50

APPENDIX D - ADMINISTRATIVE FORMS 51

APPENDIX E - MOP MODIFICATION LOG 52

APPENDIX F - STUDY ABSTRACT……………………………………………………...53

APPENDIX G – APPROVED IRB PROTOCOL …………………………………………...54

APPENDIX H – APPROVED CONSENT AND HIPAA AUTHORIZATION FORMS…..125

APPENDIX I - STUDY MEASURES AND INTERVIEWS………………………………136

1.0 Introduction

This study will improve clinical outcomes for an important, growing, and vulnerable population— nursing home resident with Alzheimer's disease or related dementias—by implementing an evidence-based intervention to improve these residents' sleep. It will also significantly increase our understanding of how to implement and sustain nursing home interventions.

The grant number is 1R61AG065619-01. The acronym for this study is Forty Winks (abbreviated 40Winks) which stands for: Frontline-staff Open, Relationally-coordinated Teams Yielding Working Initiatives to Nurture and Kindle Sleep.

2.0 Overview

The intervention being studied is a 6-week sleep-improvement intervention for nursing home residents with dementia. The intervention is examined within a 22-week investigation phase (8-week pre-intervention with two 7-day measurement periods; 6-week intervention phase; 8-week sustainment with two 7-day measurement periods and post-intervention interviews) and followed at 6 months with sustainment interviews. The team-based intervention is delivered by the nursing home clinical staff, coached by the corporate trainer.

3.0 MOP Contents and Organization

The table of contents (pp. 2-3) details the organization of this Manual of Procedures (MOP). This MOP contains all of the relevant sections and its appendices contain all of the relevant forms as described in the "NIA Guidelines for Developing a Manual of Operations and Procedures (MOP) for Multi-Site Clinical Studies" .

4.0 Abstract and Study Protocol

An abstract of the study is attached as Appendix F. The officially approved UA IRB protocol is attached as Appendix G. This document will be updated upon any approved changes.

5.0 Study Organization and Responsibilities

This section lays out personnel roster, roles and responsibilities, study organization, and study sites.

Members of the Coordinating Center (and other centers as relevant), study sites, study committees, laboratories, etc. are identified along with their roles and responsibilities. Large studies are generally depicted by an organizational chart.

5.1 Roster

a. Key Participating Institutions, Key Personnel

- **University of Alabama: Administrative Coordinating Site**
    - 1) Lynn Snow, PhD – expertise in LOCK sleep intervention, dementia, nursing home health services research; clinical geroneuropsychologist
    - 2) Patricia Parmelee, PhD – expertise in MDS, sleep assessment in older adults; social psychologist
    - 3) Project Coordinator, Research Assistant, Administrator
- **University of Texas – Houston: Data Coordinating Site**
    - 4) Robert Morgan, PhD – methodologist; expertise in CMS secondary data analysis and management including MDS data. Will oversee data coordinating center including directing data management/analysis team in all data cleaning, merging, and analyses for both primary and secondary data including obtaining secondary data sets (e.g., MDS, CASPER).
    - Data analyst, Data manager
- **University of Massachusetts at Lowell**
    - 5) Christine Hartmann, PhD – expertise in LOCK sleep intervention, implementation science, mixed methods implementation evaluation, nursing home health services research; social worker
    - 6) Megan McCullough, PhD – expertise in qualitative research; medical anthropologist
- **Brown University**
    - 7) Rosa Baier, MPH – director of Brown Center for Quality Innovation (co-funded by AHCA), expertise in quality improvement implementation, assistance with recruitment. She is currently serving in a similar role in a currently funded NIA pragmatic trial (NIA-funded METRICAL Music & Memory pragmatic trial R21/R33 AG057451; V. Mor, PI)
    - 8) Ellen McCreedy, PhD – expertise in nursing home pragmatic clinical trial implementation. She is currently serving in a similar role in a currently funded NIA pragmatic trial (NIA-funded METRICAL Music & Memory pragmatic trial R21/R33 AG057451; V. Mor, PI)
- **University of Texas – Austin**
    - 9) Kathy Richards PhD, RN – expertise in sleep, nursing home sleep trials, sleep measurement in dementia; nurse
- **Liam Fry, Austin Geriatric Specialists**
    - 10) Geriatrician; CEO and Owner of Austin Geriatric Specialists which provides the medical services for over 30 nursing homes across 6 corporations in the Austin metroplex, Chair of Geriatrics Division, University of Texas Medical School. Will provide assistance with recruitment, clinical consultation.
- **Barbara Frank & Cathie Brady, B&F Consulting**
    - National experts in NH quality improvement implementation, NH intervention refinement, train the trainer development, and frontline huddling approaches. Will refine training materials, develop implementation materials including

video development in collaboration with video producer, train and supervise corporate coaches, and support dissemination material development.

b. Contact for Special Situations

The administrative coordinating site should be contacted for most study-related questions, including Protocol requirements, Reporting an adverse event (AE) and Serious Adverse Event (SAE), Request for additional supplies, Randomizing a participant, Unblinding of data, data shipping procedures, and data transmission procedures. The data coordinating site should be contacted for questions related to CMS/MDS data acquisition/storage, dataset creation/cleaning/storage, and data analysis. The quantitative team (see 5.5) should be contacted for questions regarding data forms and quantitative data collection procedures. The intervention team should be contacted for questions regarding intervention training and delivery. The qualitative team (see section 5.5) should be contacted for questions related to qualitative data collection procedures, transmission, and analysis.

5.2 Coordinating Center

The Coordinating Center for this project is located within the Alabama Research Institute on Aging (ARIA), at the University of Alabama, located at Capitol Hall, 270 Kilgore Ln, Tuscaloosa AL 35487.

The responsibilities of the coordinating center include:

- Development and maintenance of study materials including the MOP and study forms
- Communications with all study sites, scheduling of meetings and training sessions, responding to and documenting ad hoc communications
- Developing and implementing quality control procedures
- Overseeing procedures of data coordinating site and intervention sites
- Development of the randomization scheme and procedures
- AE and SAE monitoring and reporting
- Site visits to ensure adherence to the protocol and procedures
- Reports (e.g. enrollment, adverse events, participant status, site performance, quality control, DSMB)
- Distribution of all changes, updates and policies of reports and documents to all participating study sites, NIA and to the DSMB as necessary.
- All human subject enrollment and consenting: recruitment, screening and enrollment of participants
- Protection of participants' rights
-

5.3 Study Sites

- The University of Alabama serves as the <u>administrative site</u> for the project. Drs. Snow and Parmelee, and the UA study staff have offices co-located in the Alabama Research Institute on Aging.
    - Only the University of Alabama site will enroll human subjects and the University of Alabama IRB will serve as the single IRB.
    - Dr. Snow at the University of Alabama serves as corresponding PI and will be responsible for oversight of the entire project.
    - **<u>Responsibilities</u>**: see 5.2 above
- The University of Texas at Houston serves as the <u>data coordinating site</u> for the project. Dr. Morgan and the UTSPH study staff have offices co-located at the School of Public Health.
    - **<u>Responsibilities</u>**:
    - Development of the data flow and data management procedures including data entry, error identification and correction.
    - Receiving data from intervention sites
    - Obtaining MDS data from CMS
    - Creating linked, merged, and cleaned data sets and code books
    - Conducting quantitative analyses and creating data and analysis reports provided to coordinating center
- The <u>intervention sites</u> for the study will consist of 27 nursing homes (3 in the R61 pilot year, 24 in the R33 clinical trial years) equally distributed across three NH corporations (Vivage Senior Living, Caraday Healthcare, and White Oak Management).
    - **Responsibilities:**
    - Maintenance of study binder
    - Data collection and participant follow-up through study completion
    - Transfer of data to coordinating center and resolution of all queries
    - Compliance with and accountability of administration of study intervention
    - Retention of specific records, (e.g., copies of informed consent documents in medical record)
    - Communication of questions, concerns, and/or observations to the Coordinating Center
    - Timely reporting of potential AE/SAEs to Coordinating Center
- The research will be performed by the research study team located at the University of Alabama, University of Massachusetts-Lowell (site of Co-Principal Investigator Dr. Hartmann), Brown University, University of Texas at Austin (School of Nursing and Dell Medical School), the University of Texas at Houston (School of Public Health), and B&F Consulting (Charlestown, Rhode Island).
    - **<u>Responsibilities</u>**:
    - Membership in a Steering Committee and other committees (see 5.4)
    - Compliance with protocol, MOP, IRB, Federal and state regulations

5.4 Steering Committee

- The **leadership team**, comprising Drs. Snow and Hartmann and UA research staff, will meet weekly.
    - **Responsibilities**:
    - Maintenance of study binder
    - Creation and disbanding of study subcommittees
    - Allocation of resources based on priorities of competing study demands
    - Preparation of the essential study documents, including the protocol, protocol amendments, and MOP
    - Oversight of publications and presentations
- The **steering committee (i.e., investigator team)**, led by Drs. Snow and Hartmann, includes the UA research staff, all co-investigators, and the implementation consultants: Drs. Hartmann, McCullough, Morgan, Parmelee, Richards and McCreedy, and Ms. Baier, Brady, and Frank. The team will meet monthly.
    - **Responsibilities**:
    - Design and conduct of the study
    - Participation in protocol finalization and preparation of study materials
    - Preparation of data collection forms
    - Review of data collection practices and procedures
    - Monitoring recruitment and retention of study participants
    - Changes in study procedures as appropriate
    - Review of study progress in achieving goals and taking necessary steps to ensuring the likelihood of achieving those goals
    - Review and implementation of recommendations from the DSMB
    - Review and response to other general advice and/or recommendations (e.g., from the NIA Program Director or Project Officer)
    - Participation in development of presentations and publications

5.5 Other Study Committees

The following teams will meet throughout the study period

- The **data team**, co-led by Drs. Morgan and Hartmann, includes Drs.Parmelee, and Richards, with Dr. McCreedy and Ms. Baier included for specific topics. The team will meet bi-weekly.
    - The **data core** team will serve as a sub-team led by Drs. Morgan and Hartmann and will also include UT and UA research staff.
    - The **qualitative team** will serve as a sub-team led by Dr. McCullough and will include Drs. Hartmann and McCreedy and UML and UA research staff
    - The **implementation evaluation team** will serve as a sub-team led by Dr. Hartmann
- The **intervention team**, co-led by Dr. McCreedy and Snow, includes Ms. Brady and Frank, with Ms. Baier and Drs. Richards and Fry included for specific topics. The team will meet bi-weekly.
    - The **actigraph team** will serve as a sub-team led by Dr. Snow and including Dr. Parmelee and Dr. Richards

    - The **consultant coordination team** will be led by Dr. Snow and will interface with consultants as needed (Sarah Brown-Empira, Ben McGinley-videographer, Alabama Nursing Home Association)
- The data core team, lead by Dr. Morgan,
- The **DSMB support team team (safety)**, co-led by Drs. Snow and Morgan, includes Brian Cox and Drs. Richards and Fry
    - Morgan, Cox, and Snow will oversee development of materials and reports for DSMB review and will support the open portion of DSMB meetings
    - Richards, Fry, and Snow will review all nursing home staff reports of potential AEs and SAEs for initial determination of severity, possible study relatedness, and reportability status.
- The **DSMB**, chaired by Dr. Kennedy (University of Alabama-Birmingham), includes Drs. Whyte (University of Pittsburgh) and Hodgson (Uni

5.6 NIA's Role and Responsibility

The NIA Director approves the DSMB and provides oversight, through the NIA Program Officer, of study scientific and ethical conduct and participant safety. The NIA Program Officer (or designees and contractors thereof) receives reports from the coordinating center and from the DSMB and provides oversight for DSMB meetings.

6.0 Training Plan

STUDY STAFF TRAINING

This study does involve the vulnerable population of nursing home residents with dementia. This study also involves NH employees, who are vulnerable to the extent that they may feel coerced to participate in research activities by their supervisors given that the NH leaders and parent corporation are supportive of overall study participation. To protect all vulnerable subjects, in addition to the NIH-required computer-based trainings (on the protection of human research participants, HIPAA, and Good Clinical Practice), the UA study staff will attend an in-person 2-day training session specific to the proposed project led by Dr. Snow. The PI will review the overall goals of the study, study policies and procedures, data collection manuals, adverse event identification and reporting, subject confidentiality, communication techniques and principles for working with persons with dementia, and appropriate procedures for working by telephone with LARs for consent, for working by telephone with people with dementia for consent and for determining capacity to consent. Procedures will be reviewed for protecting employees from possible coercion for participation from supervisors including maintaining confidentiality of those employees who do and do not consent to research interviews. During these training sessions, professionally produced videotapes of staff interactions with persons with dementia will be shown and the PI and study staff will role play all informed consent, capacity assessment, and data collection processes. Drs. McCullough and Hartmann will provide training on appropriate procedures for NH staff interviews. Dr. Morgan (will provide training on appropriate procedures for data safety and security.

Dr. Richards (via skype) and Parmelee (in person) will work together to provide training on appropriate assessment equipment (e.g., actigraph, fitbit) maintenance and data downloading. For example, staff will be instructed that all portions of the bands and backs of the actigraph/fitbits should be carefully and thoroughly disinfected with a Sani-Cloth prime germicidal disposable wipe (or similar product that is appropriate for healthcare equipment that comes into patient contact such as stethoscopes) and left on the device for 1 minute as per product instructions to reach maximum bactericidal/fungicidal/virucidal/tubercolocidal effectiveness. The watch front of the actigraph/fitbits should be disinfected with a Sani-Cloth Easy Screen Cleaning Wipe (or similar product that is a 70% isopropyl alcohol solution and is designed for use on touch screens and other electronic devices that are degraded by more intensive chemicals).

NH LEADERSHIP TEAM TRAINING:
Study staff will provide a 2-hour training on procedures to protect vulnerable subjects, as follows: proper procedure for, and importance of only using IRB approved procedures and letters when sending study opt-out letters to LARs;
proper procedures for maintaining security and privacy of data packages mailed to the University of Alabama project office; proper procedures for avoiding coercion or appearance of coercion of research participation when working with NH staff (e.g., not asking staff about research interview participation); proper procedures for assisting in telephone appointment arrangements and providing logistical support when study team members conduct telephone-based NH resident consenting and capacity to consent assessment; importance of only study team members conducting actual consent procedures of anyone (LARs, NH residents, NH staff); importance of only properly trained NH leadership team members participating in study procedures.

Dr. Richards and Parmelee (via skype teleconference) will work together to provide training on the appropriate application of actigraphs and Fitbits to promote comfort and prevent adverse experiences, as well as appropriate assessment equipment maintenance and data mailing. For example, the NH leadership team will be trained on the appropriate actigraph/fitbit cleaning as described in the previous subsection (STAFF TRAINING). They will also be taught that actigraphs/fitbits should only be applied to dry, clean skin. The skin should be gently washed with the skin cleanser approved for facility use for the nursing home resident (typically a non-soap cleanser such as cetaphil, but this may differ depending upon the specific resident), gently rinsed with a clean damp washcloth, and then carefully dried to avoid trapping moisture between the actigraph/fitbit and skin. Similarly, when the actigraph/fitbit is removed in the morning, the skin should be again cleansed and dried.

NH STAFF INTERVENTION TRAINING
NH Staff Training will follow train-the-trainer principles, an established, effective

mechanism for training NH staff. [37-39,81] To prepare for the training, B&F Consulting (Barbara Frank & Cathie Brady, research study team members, national NH quality improvement implementation experts, authors of two books on their method which highly influenced the development of the LOCK Sleep Program [25,35]) will guide each NH in the establishment of LOCK sleep program teams: (a) a NH Leadership team consisting of the Director of Nursing, the NH administrator, the staff educator, and the QI designee and (b) an implementation team consisting, at minimum, of the Minimum Data Set (MDS) coordinator, unit managers, and the medical director. B&F Consulting will visit each NH 5 times over the course of the R61 phase to train and support the NHs; Dr. Snow and other research staff will also attend several visits and will attend all phone calls with the sites. The corporate coaches will attend all B&F consulting visits to their corporation's NH to prepare for the subsequent randomized controlled trial (the R33 phase), when the corporate coaches will assume leadership of the training with B&F support. B&F Consulting's will conduct visits to train leadership and staff and begin the implementation, in and then conduct visits to support implementation, and then finally a visit to support sustainment.

The training will include the following components. B&F Consulting will train the leadership and implementation teams to guide staff to use huddles to identify residents with ADRD whose sleep is disrupted, explore residents' personal histories with help from family, and develop action plans to pilot test individualized, person-centered sleep improvement approaches. B&F Consulting will also guide the teams to teach staff to use observation and data collection to monitor the impact of their action plans and change the plans as appropriate. The training will also instruct staff in actigraph/fitbit use. It will include information on why good sleep is important for residents with ADRD, the etiology of poor sleep, evidence-based sleep improvement interventions (e.g., good sleep hygiene, minimizing noise and light and resident disturbance at night, maximizing engaging activity during the day, good nutrition and hydration practices to promote sleep). As an example of training in techniques to reduce night-time interruption, nursing staff will be trained on procedures for checking on the resident without disturbing them if asleep, such as the use of small flashlights pointed toward the floor using use amber illumination (rather than turning on overhead sleep disruptive blue lights) to allow the nursing staff to check the resident and room without awakening a sleeping resident. This increases safety because sleeping residents are residents who are not getting up. Whereas waking a resident to use the restroom can result in the resident not being able to easily go back to sleep, which then puts them at risk of getting up due to restlessness or agitation after the nurse leaves.

The training will emphasize the importance of customized individualized care, incremental approaches to treatment changes, and how to use huddles to integrate such interventions into individualized treatment plans. For example, "decrease in nighttime interruption" does not mean never checking on the resident during the

night. Staff will be trained on the importance of using small PDSA (plan-do-study-act) cycles to assure that all care changes are pursued incrementally in an individualized, customized manner. This is not about one-size-fits-all care. There is no blanket edict to not interrupt the patient for the entire night. Rather, the instruction is for the team to consider and explore ways of promoting longer periods of uninterrupted sleep, and to test these innovations in incremental trials over a period of days or weeks.

NH STAFF TRAINED TRAINERS PROVIDING TRAINING:
After B&F Consulting has trained the NH Leadership and implementation teams, those teams will then be responsible for training others in the NH. As part of the intervention training, NH staff will be trained by their NH Leadership or implementation teams or their trained designees on proper use and placement and cleaning of actigraphs and Fitbits, verbal and behavioral signs of distress that might indicate actigraph/Fitbit-related discomfort/distress, and appropriate modification techniques to try to relieve such distress. NH staff will be informed that the devices should be removed in the unlikely event that distress behaviors are not relieved by modification techniques. NH staff will be trained on importance of NH resident autonomy, and will be engaged in a discussion of how to assess for and honor NH resident assent or lack thereof with regard to the devices.

7.0 Communications Plan

The study team organization and meeting schedule provides an infrastructure for regular communication. The Coordinating Center will take primary responsibility oversight of documenting communications. Each meeting shall be documented with an agenda and minutes. All meeting agendas and minutes will be shared with the Coordinating Center. The weekly leadership team meeting and monthly steering committee meeting will serve as communications hubs in which a standing agenda item will be review of the list of teams and the study timeline to assure that regular communication is being received from all teams and expected progress in each area is being accomplished. The Coordinating Center will take primary responsibility for regular communication with NIA through the NIA Program Officer and/or designees, including communication about reportable SAEs/AEs, DSMB reports, update reports, and all other required reports and updates.

8.0 Study Flow

Study recruitment and data collection flow are described below separately for the two types of participants – nursing home (NH) residents and NH staff participating in interviews. As study preparation proceeds, a study flow diagram will be developed.

1. NH Residents
a. Study staff will successfully obtain all necessary IRB authorizations for protocols and materials

b. After training in eligibility criteria by study staff, NH leadership team (administrator, director of nursing, quality representative, nurse manager(s), or their designees will **identify all potentially eligible participants** [NH residents with an Alzheimer's disease or related diagnosis (ADRD diagnosis)] and the NH leadership team will mail to those residents' legally authorized representatives (LARs) an IRB-approved study introduction letter (including option to opt out via voicemail to a 1-800 number within one month if they do not wish to be contacted by study staff). Contact information of those LARs **not opting out** will be shared with University of Alabama (UA) study staff.

c. UA study staff will contact LARs and attempt to gain **informed consent** for NH resident participation. Study staff will weekly provide an updated list of consented residents with study identification numbers to the NH leadership team along with copies of all completed informed consent forms so that a copy can be filed in the resident's medical records and a copy can be stored along with the complete list and other IRB approvals and relevant study information in an essential documents binder at the NH. These processes will assure that NH staff will be able to easily verify that study staff only have access to personally identifiable data of residents are involved in the study.

d. NH staff will work together in frontline huddles to **complete screening** of all eligible residents (see 9.1 for eligibility criteria), make final determination regarding which residents with sleep problems to **enroll in the LOCK sleep program**, and **collect assessment data** for enrolled residents.

e. Every week, the NH leadership team will use researcher-provided tracked express mail service envelopes to send the UA study staff the following: 1) all completed staff rating forms, identified by NH resident subject number; and 2) any actigraphs and Fitbits for which the assessment periods are complete, identified by subject number. Research staff will mail back actigraphs and Fitbits after data download, deletion of data from the devices, and maintenance.

f. UA study staff work with each NH to extract information from the medical records of consented NH residents (this process and the information to be extracted will be clearly described in IRB-approved protocols and consent forms).

g. UA study staff will upload all data at UA and then transfer to the University of Texas School of Public Health (UTHealth SPH) Data Center via secure file transfer procedures as established by Dr. Morgan and the Data Center.

h. MDS data will be obtained by Dr. Morgan and the UTHealth SPH Data Center through data use agreement for use of personally identifiable information from CMS. MDS data for consented NH residents will be linked and merged with the other data described above.

i. When data are reported in publication and presentation they will only be reported in

aggregate form, and neither NH nor participant will be identifiable.

<u>2. NH Staff for interviews</u>
a. Study staff will successfully obtain all necessary IRB authorizations for protocols and materials described below.

b. NH leadership team will provide to all NH staff an IRB-approved study introduction letter (including option to opt out within one month if they do not wish to be contacted by study staff). Contact information of those NH staff not opting out will be shared with UA study staff.

c. UA Study staff will contact NH staff to invite them to participate in mid-implementation phone interviews. Study staff will also contact NH staff to invite them to participate in post-implementation interviews. The list of NH staff who agree and decline interview participation will remain confidential to protect NH staff from any possible coercion to participate (see 1.3.b. Protection Against Risks).

d. UA study staff will consent participating NH staff and conduct interviews. Interviews will be audio recorded. Recordings will be uploaded at UA, identified only by subject number, and shared with a university-approved transcription service via secure file transfer procedures as established by Dr. Morgan and the UTHealth SPH Data Center. Transcribed files, identified only by subject number, will be returned via the same secure file transfer procedures to UA for tracked storage and analysis and to Drs. McCullough and Hartmann at UML for analysis.

e. When data are reported in publication and presentation they will only be reported in aggregate form, and neither NH nor participant will be identifiable.

9.0 Recruitment and Retention

For recruitment of three nursing home corporations, we will work with and have letters of support from (see Letters of Support) the Alabama Nursing Home Association; Austin Geriatric Specialists in Austin, Texas; and the American Health Care Association (AHCA; representing more than 11,000 NH facilities). The Brown Q&I Center was established with funding from the AHCA and will also work with us in recruitment (also see Letters of Support). This support ensures we will be able to recruit 3 NH corporations to participate in this trial. Participating corporations will have a minimum of 12 NH sites to facilitate randomization. Participating corporate leaders will agree to (a) a random, phased introduction of the intervention, (b) devote centralized corporate training staff to implement the intervention (corporate coaches), and (c) work with study staff to execute secure data transfer protocols.

For our R61 phase, we will work closely with the 3 recruited corporations to identify 3 NHs (1 per corporation) to participate. Stakeholder engagement will occur at both the

corporate and the NH levels. At the corporate level, each corporation will assign a corporate coach who will attend all B&F Consulting visits to their corporation's NH to learn how to prepare NHs for and support NHs in the R33 implementation of the LOCK sleep program. At the NH level, each NH will create LOCK sleep program leadership and implementation teams and will support frontline staff involvement in the LOCK sleep program.

Screening and recruitment of NH residents and NH staff is described in sections 8.0 and 9.1

9.1 Screening and Eligibility Criteria

See section 8.0 for screening criteria and flow.

9.2 Screening Log

The screening log is included in Appendix B.

9.3 Eligibility Criteria

NH RESIDENT INCLUSION AND EXCLUSION CRITERIA:
In the potential participant pool, we will include all NH residents aged >=50 years with an ADRD diagnosis. We include residents across the range of ADRD severity because this is consistent with the LOCK sleep program, in which staff will focus on residents with ADRD who have the greatest sleep problems without differentiating by ADRD severity. To identify participants, NH staff will use frontline staff huddles. NH staff will be trained to use the STOP-Bang screening tool to identify NH residents with high risk of obstructive sleep apnea (OSA) [82-84] and will be trained on appropriate procedures for referring any positively screened residents for medical evaluation. We will exclude residents with a high risk of OSA who are not being treated for OSA because actigraph measurements are inaccurate in that population.[85] Staff will also exclude residents who have a persistent bilateral resting tremor or paralysis in both arms (a subset of persons with Parkinson's disease and related significant tremor-causing diagnoses), due to actigraph measurement inaccuracies.[86]

NH STAFF INCLUSION AND EXCLUSION CRITERIA:
Staff will not be excluded on the basis of race, ethnicity, gender, or age. As described above, a subset of NH staff will be recruited for interview participation based upon job type and intervention engagement (e.g., frontline staff versus leadership/interdisciplinary team members).

<u>10.0 Informed Consent</u>

NH RESIDENT CONSENT: We will ask the administration department at each NH to compile a list of all NH residents with an ADRD diagnosis. The administration department will send a letter to each resident's legally authorized representative (LAR) informing them of the study and inviting the LAR to opt out if they do not wish to be contacted by research staff. Opt-out procedures have been demonstrated to

yield higher response rates and lower rates of non-response bias compared to opt-in procedures, and to be acceptable to participants, with no difference in rates of reported distress or complaints compared to opt-in procedures. [105-108] In our past federally funded research involving people with dementia, opt-out consent has been approved by multiple IRBs and worked well. Contact information of those LARs not opting out will be shared with University of Alabama (UA) study staff. We are requesting of this IRB a waiver of consent and HIPAA authorization for screening purposes regarding the sharing of these contact information. Research staff will contact all LARs who do not opt out, inviting the LAR to consent to the NH resident's participation. The consent process will be described in more detail in the consent portion of this IRB proposal.

NH STAFF CONSENT: The NH leadership team or their designees will provide to all NH staff an IRB-approved study introduction letter (including option to opt out within two weeks if they do not wish to be contacted by study staff). Contact information of those NH staff not opting out will be shared with UA study staff. We are requesting of this IRB a waiver of consent and HIPAA authorization for screening purposes regarding the sharing of these contact information. Research study staff will contact NH staff (via email and/or phone) to invite them to participate in mid-implementation and/or post-implementation interviews about their experiences with the LOCK sleep program and Fitbits. The list of NH staff who agree and decline interview participation will remain confidential to protect NH staff from any possible coercion to participate. The consent process will be described in more detail in the consent portion of this IRB proposal.

NOTE: The LOCK sleep program itself comprises clinical practices common in NH QI efforts. These clinical practices that will be taught via the LOCK sleep program training thus fall within the scope of staff positions and require no consent.

10.1 HIPAA Authorization

The Health Insurance Portability and Accountability Act authorization form may be a separate document from the informed consent, and must be reviewed and signed by the study participant in addition to reviewing and signing the consent form. The format of the HIPAA authorization is established by the local IRB.

The UA IRB approved HIPAA Authorization and Waiver of HIPAA Authorization are attached in Appendix H.

11.0 Study Intervention

NH STAFF INTERVENTION IMPLEMENTATION

The 3 NHs will implement the program starting at approximately the same time. Based on prior experience (both in VA and in B&F's experience in non-VA NHs), we anticipate an initial 8-week period in which each NH builds its frontline staff huddle practice across all units (Figure 2: pre-implementation). After this, each NH will begin the LOCK sleep intervention on all its units (Figure 2: implementation). An 8-week

sustainment period follows (Figure 2). The leadership team will communicate broadly about the program, ensuring all staff (including new hires) are introduced to and maintain implementation of the LOCK sleep program and perform the periodic sleep measurements. See section 6 (training) for information about specific intervention content.

Figure 2: Example Step's Measurement and Intervention Timing and Spacing

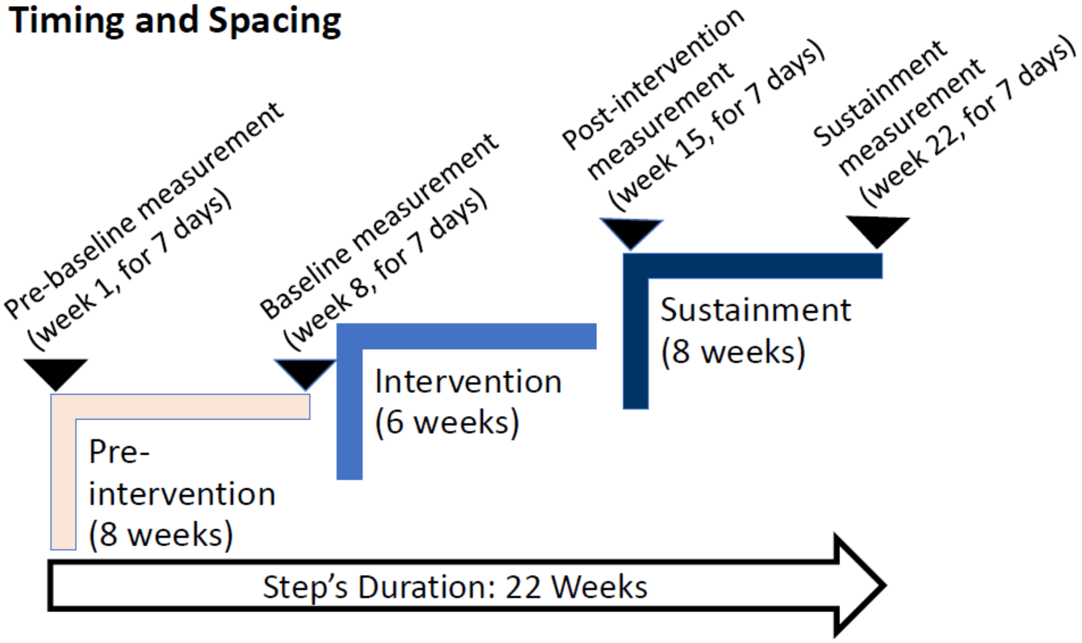

12.0 Randomization

The unit of random assignment is the NH. We will assign all NHs to the control group and phase them into the intervention group at 7-week intervals (steps). See Table 1. Our stepped wedge design has 6 steps with 4 NHs per step. Each step involves a 22-week intervention period (Table 1). We will randomly assign the order in which the NHs are phased into the intervention group. 114 To include 4 NHs per step in a balanced manner, corporations A, B, and C will have, respectively, 2, 1, and 1 NHs participating in step 1; 1, 2, and 1 NHs in step 2; 1, 1, and 2 NHs in step 3; 2, 1, and 1 in step 4, etc. NHs will be stratified by corporation for assignment to implementation step. We will randomize NHs within corporations to steps after matching on bed size and number of long-stay residents with ADRD diagnoses. Total estimated time to complete all 6 steps is 57 weeks (1 year and 5 weeks).

Our design is an incomplete stepped-wedge cohort design, 115 where each NH serves as its own control and residents within NHs are followed longitudinally (from pre- through post-intervention measurement periods). It is well-suited for the proposed study as it is effective for evaluating interventions implemented at the NH level with strategies that target groups rather than individuals.116,117 The greatest disadvantage to the

stepped-wedge design is that it is not a parallel group experimental design that has randomization of groups to intervention or control. We initially considered using a traditional cluster randomized parallel group design, with a group of NHs randomized to receive attention control and another group randomized to receive the intervention. But with this approach, our sample size calculations required approximately twice the number of NHs, presenting both financial and logistical constraints due to the complexity of delivering both the LOCK sleep intervention and the attention control across a large number of NHs. Since the design effect of the stepped wedge approach requires a substantially smaller sample than a parallel group design, 118 and with actigraphs costing approximately $500 per device, this is a consideration. Our incomplete stepped-wedge design thus offers a pragmatic alternative. It enables all sites to receive the intervention and enables data analysis options of between- and within-NH comparisons and assessment of time-based variations in intervention effects. We therefore believe our design to be best-suited for this pragmatic trial.

Table 1: Stepped Wedge Design with Measurement Periods

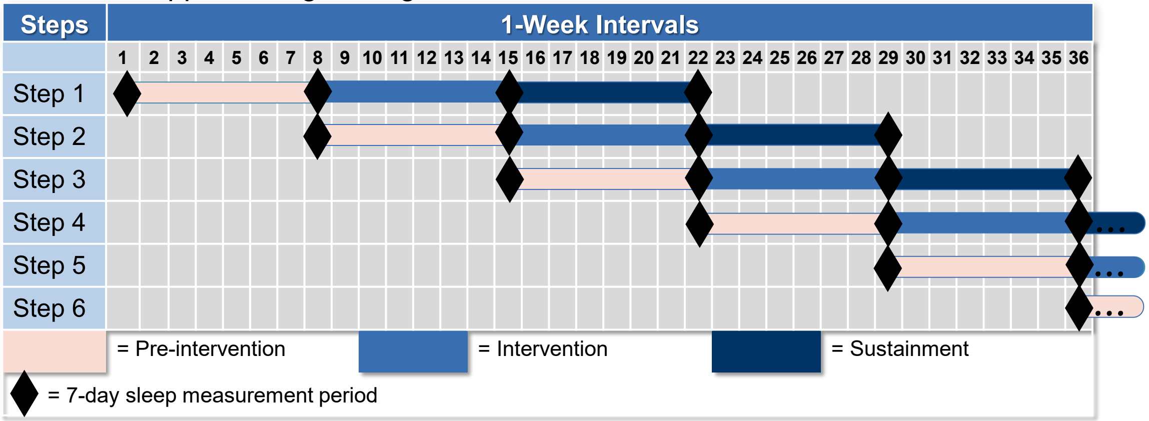

13.0 Blinding and Unblinding (Masking and Unmasking)

This study is not blinded. It is a randomized controlled trial using an incomplete stepped-wedge design.

14.0 Study Measurements and Procedures

This section addresses how assessments are conducted to assure consistent performance of assessments. The actual measures are addressed in section 18 (data collection).

NH RESIDENT DATA will be collected for consented NH residents from NH staff ratings (clinical global assessments of change in overall sleep quality and sleeprelated conditions - see Appendix I); through primary data collection (actigraph/Fitbit measurements; these are wristwatch-sized devices worn on the wrist); through medical records (age, diagnoses, medical history, and medications); and through NH secondary data from the Minimum Data Set (MDS; all Medicare-reimbursed NHs are required to collect MDS assessments on all residents; the MDS contains information on resident medical conditions, functioning, cognition, psychotropic and pain/analgesic medication use, activities of daily living decline, and sleep-, mood-, and activity-related items). Table 2 summarizes the primary and secondary outcomes and data sources.

Table 2: Primary and Secondary Outcome Measurements, Supplementary Sleep Data, and Data Sources

Outcomes & Supplementary Data	Data Sources	Relevant MDS Sections
**Primary outcome:** Sleep	- Actigraph	
**Secondary outcome:** Psychotropic medication use	- MDS	N0410A-C, N0450A, V0200A17A
**Secondary outcome:** Pain and analgesic medication use	- MDS	J0100A-C; J0300-J0600; J0800A-D
**Secondary outcome:** Activities of daily living decline	- MDS	G0110A-J and G0120A-B; QM N028.01
**Supplementary sleep data:** Sleep via staff rating	- Staff rating	
**Supplementary sleep data:** Staff-identified sleep-related concerns	- Staff rating	
**Supplementary sleep data:** Inter-resident sleep variability	- NH's medical record	
**Supplementary sleep data:** Sleep from MDS	- MDS	D0200C, J0500A

NH STAFF DATA staff interview data will be collected via audiorecorded qualitative interview (see Appendix I).

DATA COLLECTION PROCEDURES:
NH RESIDENT PRIMARY RESEARCH DATA COLLECTION will consist only of

actigraph/fitbit data.
We will measure total sleep time (TST) with data from Micro-Mini Motionlogger Actigraphs (Ambulatory Monitoring Inc., Ardsley, NY).[87] We will define nighttime as 10pm to 6am and will compute TST as the total number of minutes asleep during a nighttime period. We will measure sleep for 7 days at each measurement period (see Figure 2) to obtain reliable measurements and will use the average TST across this period. We will also examine the following: wake after sleep onset (total number of minutes awake during nighttime), how often the resident awoke during the nighttime, sleep efficiency (the ratio of minutes asleep to minutes awake over the period), and sleep fragmentation (an index of restlessness computed as the percentage of one-minute epochs scored as awake). Sensitivity of actigraph measurement for sleep is very good (actigraphy = sleep when polysomnography = sleep was 0.97). [88]

We will also explore measuring sleep time using Fitbits. Fitbits are known to be inferior to actigraphs for sleep research measurement purposes because Fitbits tend to overestimate total sleep time, among other limitations.[70,71] But Fitbits are considerably less expensive than actigraphs, offer an easy user interface, and collect data on sleep plus multiple other dimensions, making them potentially useful to NH staff for non-research purposes. Studies are lacking on NH staff impressions of the perceived potential usefulness of Fitbits for informal measurement. We will therefore explore staff impressions of Fitbits through semi-structured interviews after the R61's 22-week measurement period. If staff find Fitbit information of added use and staff and residents consistently find it acceptable to use 2 devices at the same time (actigraph plus Fitbit), we will consider use Fitbits beyond this pilot study into the randomized controlled trial (R33 phase).

Dr. Kathy Richards, PhD MSN (nurse co-investigator with extensive NIA and VA principal investigator experience using objective sleep measurements including actigraphs in studies with NH residents and people with dementia, who tolerated the devices well without removing them.[109-111] NH staff will assist residents to wear both an actigraph and a Fitbit side by side on a wrist for 7 days during each of the 4 measurement periods (weeks 1, 8, 15, and 22—see Figure 2). We will use Dr. Richards' effective procedures for assuring that NH staff maintain charge and do not lose actigraphs or Fitbits (e.g., Nighttime Agitation and Restless Legs Syndrome in People with Alzheimer's disease, R01 AG051588-01A1, NIH-funded).

NH STAFF DATA ABOUT NH RESIDENTS: The NH Leadership and NH Implementation teams will be trained by our research study team on the LOCK Sleep Program methods (see Figure 1), including how to facilitate and implement front-line staff huddles to discuss resident sleep issues. Once this training is complete, we will ask NH huddle facilitators to use a huddle to establish a team consensus rating of each enrolled NH resident's overall sleep quality and up to 2 additional symptoms or behaviors of concern potentially related to sleep quality

(e.g., agitation, pain). Ratings will be obtained using the using the Clinical Global Impression of Change rating scale (CGIC; see Appendix I); CGIC ratings evidence high reliability and responsivity under a wide range of circumstances and are frequently used as outcomes in clinical trials. [97-104] Each LOCK sleep program huddle facilitator will lead their huddle team to complete clinical global impression of change ratings of enrolled residents' overall sleep quality at the end of each 7-night sleep measurement period and at the end of each week of the 6-week sleep intervention period. These rating forms will be part of the standard care practices being taught to the NH staff -- such assessments are within the typical scope of staff responsibilities. The NH clinical team will file these assessments in the NH residents' medical chart. The NH Leadership team will copy these rating forms and share with the research team.

NH STAFF INTERVIEWS: We will recruit and consent a sample of NH staff engaged in huddles to participate in mid- (phone) and post-implementation semi-structured interviews to explore staff perceptions of the LOCK sleep intervention's effectiveness, feasibility, facilitators, and challenges, as well as their impressions of the interview process itself to inform for the upcoming randomized controlled trial (R33 phase) (e.g., length, location, etc. for busy NH staff) and use of Actigraphs and Fitbits (see Appendices for interview guides). For the post-implementation interviews, we will send a research staff member trained in qualitative interviewing to each site to conduct these interviews. For both interviews, we will recruit the LOCK sleep program leadership and implementation team members, as well as a sample of frontline staff, with an approximate sample size of 20 staff per NH.

MEDICAL RECORD/SECONDARY DATA ABOUT NH RESIDENTS: To assess inter-resident variability in sleep, a research assistant will work with the NH at the end of the 22-week measurement period to extract data from the NH's medical record on the following for the entire period: (a) changes in any sedating medications and changes in dosages; (2) incidents of delirium; (3) any urinary tract infections; (4) doses of any sedating medications, including as needed ones. Also collected from medical records will be information to characterize the NH residents (age, diagnoses, medical history, and medications). NH secondary data will be collected also from the NH Minimum Data Set (MDS; all Medicare-reimbursed NHs are required to collect MDS assessments on all residents; the MDS contains information on resident medical conditions, functioning, cognition, psychotropic and pain/analgesic medication use, activities of daily living decline, and sleep-, mood-, and activity-related items).

Figure 1: LOCK Elements, Corresponding Evidence-Based Concepts, and Examples

LOCK Element	Evidence-Based Concept & Explanation	Fictional Example Relating to Sleep
Learn from bright spots	Strengths-based learning: When searching for solutions to an issue, look for positive outliers (i.e., positive deviants) to identify instances of success from which to learn.	Ms. Smith doesn't sleep well. But last weekend she slept straight through two nights in a row. What happened during that time? Staff investigate and discover Ms. Smith's roommate's bed alarm broke and wasn't replaced until after the weekend. And Ms. Smith's daughter was in town and took her for long walks outside.
Observe	Observation: Have staff briefly step back from their regular routines and conduct specific observations to collect data, using structured tools or not. These observations provide the fodder for huddle dialogues.	A main contributor to Ms. Smith's poor sleep is her low daytime activity level and noise at night. Nursing uses an actigraph to observe the quantity and quality of her sleep at night. Nursing and recreation work together to observe and informally track Ms. Smith's daytime activity level. All staff in the neighborhood observe indications of Ms. Smith's activity and behavior during the day and night, particularly in response to noise.
Collaborate in huddles	Relationship-based teams: Conduct brief, collaborative, strengths-based frontline staff huddles to discuss risk factors for an issue, bright spots, results of observations, and action planning.	In a frontline staff huddle, the facilitator moves the conversation about Ms. Smith through the steps of bright spot exploration, hypothesis generation based on observation, and action planning, making sure everyone is heard. Staff decide to offer Ms. Smith a variety of activities to increase her daytime activity level. On Monday, the group determines to pilot 3 new options. The group agrees to huddle Thursday to discuss what worked and what other activity options to pilot.
Keep it bite sized	Efficiency: Keep all LOCK components to 5-15 minutes. Incremental changes, rather than systemic overhauls, are easier to integrate.	Staff shorten existing meetings by 5-10 minutes to make room for frontline staff huddles. Staff huddle more frequently for shorter intervals to facilitate timely information exchange. Huddle facilitators ensure pilot changes resulting from action plans are small enough to fit into routines but meaningful.

14.1 Timeline and visit schedule

Each site will serve as its own control.

Control data will be collected for 8 weeks, then the intervention will begin and intervention data will be collected for 6 weeks, then the sustainment phase will begin and sustainment data will be collected for 8 weeks.

The duration of the intervention period for any NH is 22 weeks, which includes four 7-day sleep measurement periods (pre-baseline measurement before week 1, baseline measurement at week 8, post-intervention measurement at week 15, and sustainment measurement at week 22).

As study preparation proceeds, a schedule of visits and evaluations and a visit and evaluation tracking sheet will be developed to allow for tracking of what is done in each study phase and at each contact with the study participant. Appendix C will be refined at this time.

14.2 Scope/Schema

Study visits are described in sections 6 and 19.

14.3 Final Study/Early Discontinuation Evaluations

Participants will be actively followed through all study visits through the final visit. If a study participant is discontinued from treatment, he/she should still be followed to the end of the study.

Early discontinuation will occur if the participant evidences verbal and behavioral signs of distress due to study inclusion, in particular behaviors that might indicate actigraph/Fitbit-related discomfort/distress. NH staff will be trained in appropriate modification techniques to try to relieve such distress. NH staff will be informed that the devices should be removed in the unlikely event that distress behaviors are not relieved by modification techniques. NH staff will be trained on importance of NH resident autonomy, and will be engaged in a discussion of how to assess for and honor NH resident assent or lack thereof with regard to the devices. NH staff will working with study staff to document early discontinuation and reason for discontinuation. There will be no final study/early discontinuation visit beyond the staff huddling intervention procedures as described in the intervention and timeline sections.

15.0 Concomitant Medications

This study does not require any concomitant medications and does not restrict any medications.

16.0 Safety Reporting

This section of the MOP details the definitions of and procedures for reporting adverse events and serious adverse events, as applicable, following the Adverse Event (AE) and Serious Adverse Event (SAE) Reporting Guidelines and Events Process Flow.

DEFINITIONS

We will follow OHRP (https://www.hhs.gov/ohrp/regulations-and-policy/guidance/reviewing-unanticipated-problems/index.html#Q2) and UA IRB guidance and policies regarding definitions, collection, and reporting of Adverse Events (AEs) and Serious Adverse Events (SAEs).

Adverse Event: any untoward or unfavorable medical occurrence in a human subject (encompassing both psychological and physical harms), including any abnormal sign (for example, abnormal physical exam or laboratory finding), symptom, or disease, *temporally associated* with the subject's participation in the research, whether or not considered *related* to the subject's participation in the research (emphasis ours).

Serious Adverse Event: life-threatening (places a subject at immediate risk of death from the event) or results in death; results in hospitalization or prolongation of an existing hospitalization; results in a persistent or significant disability/incapacity; results in a congenital anomaly or birth defect; or based on appropriate medical judgment, may jeopardize the subject's health and may require medical or surgical intervention to prevent one of the other outcomes listed in this definition (e.g., allergic bronchospasm requiring intensive treatment in the emergency room or at home; development of drug dependency or drug abuse).

CLASSIFICATION OF SEVERITY AND STUDY RELATEDNESS

Severity Definition: Severity is defined as the intensity of an event. This is separate from the seriousness of the event and is one factor considered in determining the seriousness of the event.

Severity Classification:

- **Mild:** Awareness of signs or symptoms, but easily tolerated and are of minor irritant type causing no loss of time from normal activities. Symptoms do not require therapy or a medical evaluation; signs and symptoms are transient.
- **Moderate**: Events introduce a low level of inconvenience or concern to the participant and may interfere with daily activities, but are usually improved by simple therapeutic measures; moderate experiences may cause some interference with functioning
- **Severe**: Events interrupt the participant's normal daily activities and generally require systemic drug therapy or other treatment; they are usually incapacitating

Expectedness Definitions

- **Unexpected** - Nature or severity of the event is not consistent with information about the condition under study or intervention in the protocol, consent form, product brochure, or investigator brochure.
- **Expected** - Event is known to be associated with the intervention or condition under study.

Study Relatedness Definitions:

- **Definitely Related**: The adverse event is clearly related to the investigational agent/procedure – i.e. an event that follows a reasonable temporal sequence from administration of the study intervention, follows a known or expected response pattern to the suspected intervention, that is confirmed by improvement on stopping and reappearance of the event on repeated exposure and that could not be reasonably explained by the known characteristics of the subject's clinical state. As per OHRP guidance we will consider adverse events that are determined to be at least *partially* caused by the procedures involved in the research to be categorized as related to participation in the research.

- **Possibly Related**: An adverse event that follows a reasonable temporal sequence from administration of the study intervention follows a known or expected response pattern to the suspected intervention, but that could readily have been produced by a number of other factors.
- **Not Related**: The adverse event is clearly not related to the investigational agent/procedure - i.e. another cause of the event is most plausible; and/or a clinically plausible temporal sequence is inconsistent with the onset of the event and the study intervention and/or a causal relationship is considered biologically implausible.

Combined Expectedness and Relatedness Categories: we will use four categories [1) Expected AEs *unrelated* to the study/intervention, 2) Expected AEs *related* to the study/intervention, 3) Unexpected AEs *unrelated* to the study/intervention, and 4) Unexpected AEs *related* to the study/intervention] and give study-specific anticipated examples for the first three categories below.

- Expected AEs *unrelated* to the intervention: NH residents enrolled in the trial will have dementia and likely will have other pre-existing significant health issues that require NH placement. It is anticipated that some participants will experience health events (new conditions or worsening of previous conditions), hospitalization, or death during the trial that will be due to the dementia and/or other pre-existing significant health issues and unrelated to the intervention.

- Expected AEs *related* to the intervention: We do not expect SAEs related to the intervention. Intervention-related AEs for NH residents might include: a) actigraph/fitbit-related skin reactions such as redness, itching, or other skin conditions or discomfort; b) distress or discomfort associated with actigraph/fitbit-related wear or NH staff attention; c) distress or discomfort associated with other LOCK sleep intervention NH staff attention or requests. Intervention-related AEs for NH staff might include distress related to interview questions. Such events may be reportable depending upon the severity and seriousness of the event and increase of risk to participant. The NH's treating clinical team's opinion of the seriousness of the event will be solicited and the PI, co-investigator nurse scientist Dr. Richards and study consultant geriatrician Dr. Fry will be consulted to determine seriousness and reportability of the event.

- Unexpected AEs and unanticipated problems *related* and *unrelated* to the intervention: Unanticipated AEs and unanticipated problems will be evaluated for severity and relatedness with input from the NH's treating clinical team, the PI and clinical study team members Drs. Richards and Fry and data-related study team members including data coordinating center leader and study statistician Dr. Morgan (if applicable). See paragraph 3, section 1.2.3.

Guiding Principle: As per OHRP guidance, serious AEs that are unanticipated and related/possibly related to participation in research will be considered the most important subset of adverse events because they always suggest that research increases risks to subjects and routinely warrant consideration of substantive changes in the research protocol, informed consent document, or other corrective actions.

AE/SAE REPORTING

- Serious AEs that are unanticipated (i.e., have not been previously reported for the study's intervention) will be reported within 48 hours of the study staff's knowledge to the NIA Program Officer, DSMB Chair, and UA IRB.
- Non-serious AEs that are unanticipated and study-related or possibly related, and may suggest that participants or others are at placed at a greater risk of physical or psychological harm than was previously known or recognized, will be reported to the NIA Program Officer, DSMB Chair, and UA IRB within 2 weeks of event.
- A summary of all other SAEs and AEs will be reported quarterly to the NIA Program Officer and the DSMB Chair and annually to the UA IRB.

Corporate coaches and NH leadership teams will receive training from study staff about AEs and SAEs and the importance of reporting all AEs and SAEs to study staff within 24 hours. Training will include definitions and examples and how to communicate AE and SAE information to the study team. The Corporate coaches and NH leadership teams will then train all participating NH clinical team members on these procedures.

When contacted by NH staff about a possible AE or SAE, study team members will collect and record information on all AEs and SAEs using AE and SAE study forms (including treating team's impression of severity and seriousness) and immediately share with Dr. Snow, Dr. Richards (co-investigator nurse scientist), and Dr. Fry (study consultant geriatrician) for review and initial determination of severity and possible study relatedness. Data-related study team members including data coordinating center leader and study statistician Dr. Morgan will be also consulted if applicable.

Study staff will continue to follow up with the participant and their treating clinical team until any clinical events have resolved. The PI or designee will also immediately communicate with the NH leadership team and corporate coach at the NH for immediate investigation by the relevant treating team regarding any SAEs that are unanticipated or AEs that are determined to suggest participants or others are placed at a greater risk of physical or psychological harm than was previously known or recognized. Any insights about any possible iatrogenic effects of any components of the intervention will be immediately shared with all active NH intervention sites.

As study preparation proceeds, reporting forms for Adverse Events and Serious Adverse Events will be refined and finalized.

RESPONSIBILITIES

DSMB responsibilities are to:

At Initial meeting:

- Review the entire IRB-approved study protocol and the Manual of Procedures (MOP), with regard to participant safety, recruitment, randomization, intervention, data management, quality control and analysis and the informed consent document.

- Identify the relevant data parameters and the format of the information to be regularly reported.

- Recommend stopping rules to guide unexpected causes of termination, as applicable.

- Recommend participant recruitment be initiated after receipt of a satisfactory protocol. If the need for modifications to the protocol, the MOP, consent form, DSMP or any other study document is indicated by the DSMB and/or the NIA Program Officer (PO), the DSMB will postpone its recommendation for the initiation of participant recruitment until after the receipt of a satisfactory revised protocol(s) or other study documents.

During the study meetings:

- Review masked and unmasked data. These data can be related to safety, recruitment, randomization, retention, protocol adherence, trial operations, data completeness, form completion, intervention effects, gender and minority inclusion.

- Identify needs for additional data relevant to safety issues and request these data from the study investigators.

- Propose additional analyses and periodically review developing data on safety and endpoints.

- At each meeting, consider the rationale for continuation of the study, with respect to progress of randomization, retention, protocol adherence, data management, safety issues, and outcome data (if relevant) and make a recommendation for or against the trial's continuation.

- Review and make recommendations on proposed protocol changes, and/or new protocols proposed during the trial. When the DSMBs are unblinded, the Boards may recommend to NIA to appoint a blinded working group of the DSMB to review the proposed protocol changes and make recommendations to NIA on whether to approve the requests.

- Provide advice on issues regarding data discrepancies found by the data auditing system or other sources.

- Review manuscripts of trial results if requested by the Board or the NIA PO who may seek DSMB review of manuscripts reporting major outcomes prior to their submission for publication.

Individual members' and associated personnel responsibilities include:

- The Chair of the DSMB will be responsible for the following: 1) developing the meeting agenda; 2) requesting information for the meetings from the PI and the study statistician; 3) overseeing the meetings; 4) verifying that the reports and recommendations prepared are an accurate and complete record of the DSMB's deliberations; 5) serving as contact person for unanticipated problem and serious adverse event reporting.

- The DSMB members will 1) familiarize themselves with the research protocol and consent forms, 2) review interim reports of adverse events, 3) review interim analyses of outcome data as it relates to safety, 4) review interim reports of trial participant accrual, and 5) make recommendations to the investigators concerning continuation, termination, or modification of the trial.

- The study biostatistician, Dr. Robert Morgan, will serve as the liaison to the DSMB to provide them with all necessary data and reports and be available (in conjunction with the PI) to answer any DSMB questions and requests.

- Together the PI and Dr. Morgan will be responsible for coordinating activities of the DSMB including the following: 1) Arranging DSMB meetings and communications. 2) Identifying and reviewing materials to be presented to the DSMB, in conjunction with the DSMB Chair and NIA PO.

17.0 Study Compliance

Protocol deviations/violations include, but are not limited to, the following:

- Failure to obtain Informed Consent
- Failure to keep IRB approval up to date
- Failure to report AEs/SAEs as per reporting schedule
- Failure to document treatment administration
- Participant in treatment condition does not receive treatment as per schedule
- Study assessment does not occur as per assessment schedule

The coordinating center will conduct quarterly audits and any protocol deviations affecting participant safety will be reported as per SAE/AE reporting schedule (below) or if not defined with that schedule then within 24 hours of occurrence, or as soon as they are discovered to the co-Principal Investigators, the IRB, DSMB, and NIA Program

Officer. Other protocol deviations will be reported as soon as they are discovered to the IRB and a log shall be maintained and reported routinely in the regular reporting schedule to the DSMB and NIA Program Officer.

SAE/AE REPORTING SCHEDULE
Serious AEs that are unanticipated (i.e., have not been previously reported for the study's intervention) will be reported within 48 hours of the study staff's knowledge to the NIA Program Officer, DSMB Chair, and UA IRB.

- Non-serious AEs that are unanticipated and study-related or possibly related, and may suggest that participants or others are at placed at a greater risk of physical or psychological harm than was previously known or recognized, will be reported to the NIA Program Officer, DSMB Chair, and UA IRB within 2 weeks of event.
- A summary of all other SAEs and AEs will be reported quarterly to the NIA Program Officer and the DSMB Chair and annually to the UA IRB.

As study preparation proceeds, protocol deviation reporting forms (Protocol Deviations Form Template) will be refined and finalized.

18.0 Data Collection and Study Forms

This section describes the study's data collection procedures. Data management procedures are addressed below in section 19.0

18.1 Participant Binder

This section describes how participant data are maintained in the study. All essential study documents will be retained by the coordinating center in electronic participant binders and will include the following:

- Source documents (e.g., forms completed by NH enrollment sites)
- Signed consent and HIPAA authorization forms
- CRFs
- Data correction forms
- Workbooks

18.2 Study Forms

Sources of research data are summarized in Table 2:

Table 2: Primary and Secondary Outcome Measurements, Supplementary Sleep Data, and Data Sources

Outcomes & Supplementary Data	Data Sources	Relevant MDS Sections
**Primary outcome:** Sleep	- Actigraph	
**Secondary outcome:** Psychotropic medication use	- MDS	N0410A-C, N0450A, V0200A17A
**Secondary outcome:** Pain and analgesic medication use	- MDS	J0100A-C; J0300-J0600; J0800A-D
**Secondary outcome:** Activities of daily living decline	- MDS	G0110A-J and G0120A-B; QM N028.01
**Supplementary sleep data:** Sleep via staff rating	- Staff rating	
**Supplementary sleep data:** Staff-identified sleep-related concerns	- Staff rating	
**Supplementary sleep data:** Inter-resident sleep variability	- NH's medical record	
**Supplementary sleep data:** Sleep from MDS	- MDS	D0200C, J0500A

Primary outcomes requiring a CRF are included in Appendix I.

As study preparation proceeds, this section will be elaborated and refined to include:

- List and description of study forms and their collection schedule
- Forms maintenance

18.3 General Instructions for Completing Forms

Both paper and electronic CRFs are used in this study. As the study proceeds and procedures are refined and expanded, this section will include a set of instructions for completing the CRFs to ensure quality and consistency in data collection, including a set of guidelines for incomplete or illegible forms.

18.4 Data Flow

Data flow, data entry, and data correction procedures are addressed below in section 19.0

18.5 Administrative Forms

Administrative forms will provide documentation of study processes and assist with study operations (e.g., screening log). As administrative forms are developed, they will be documented here and in relevant appendices.

18.6 Retention of Study Documentation

NIH policy requires that studies conducted under a grant retain participant forms for three years, while studies conducted under contract must retain participant forms for seven years. These are the most rigorous requirements (compared to UA IRB, etc.) and thus will guide study conduct.

19.0 Data Management

Dr. Morgan and the UTHealth SPH Data Center (data coordinating center) will create protocols for overseeing the security and protection of all data collected for this study. Personally identifiable information will be only be stored in the secure and securely backed up UA data server and UTHealth SPH Data Center in password protected files. Other collaborating sites (University Massachusetts Lowell, Brown University, and University of Texas at Austin) will only work with data identified by subject number.

STUDY SITE->ENROLLMENT SITE
The coordinating center and data coordinating center will work together with the NH corporations to assure that each NH enrollment site is provided with all data entry forms both electronically and hard copies. All parties will work together to develop the protocol that is most convenient and least likely to result in errors – this will likely be a combination of electronic and paper data entry.

ENROLLMENT SITES->COORDINATING CENTER: Every week, the NH Leadership team will use a researcher-provided tracked insured express mail service (e.g., UPS) to send the research team at the University of Alabama all completed Clinical Global Impressions of Change staff rating forms and any actigraphs and Fitbits for which the assessment periods are complete. Research staff will mail back actigraphs and Fitbits after download and maintenance. We considered electronic data transmission options but opted for mail for the following reasons: (a) We will be able to assure equipment maintenance and appropriate data downloads, reducing missing data errors. (b) For busy NH staff, copying and packaging will take significantly less time and technological skill than scanning, uploading, and transmitting electronic data.

COORDINATING CENTER->DATA CENTER After research staff upload the data at the University of Alabama, they will transfer the data to the University of Texas Health Science Center at Houston School of Public Health (UTHealth SPH) Data Center via secure file transfer procedures established by the Data Center.

CMS->DATA CENTER
The data center will oversee the process of obtaining secondary MDS data from CMS, developing and merging that data with the primary datasets, and completing any necessary documentation and reporting back to CMS. This section will elaborate these processes.

DATABASE DEVELOPMENT AND MANAGEMENT

As procedures are elaborated and developed over time, this section will be expanded to include additional information regarding how data are to be collected, entered (e.g., if eCRFs are used), edited, and corrected.

This section will include systems or procedures that encompass the following functions:

- **Data Completeness**: procedures to ensure all forms are complete, intact, and entered into the electronic CRF (eCRF)
- **Data Tracking** - to provide the status of enrollment, number of forms completed at the sites and number of forms transmitted to a Coordinating Center or lead site, as appropriate.
- **Data Entry** - that is easy to use and minimizes errors,
- **Data Editing** - that identifies out-of-range and missing entries, errors in dates and logical inconsistencies (e.g., first treatment date precedes protocol start date or protocol specifies an examination before randomization, but the examination form is missing).
- **Updating** - to correct data and maintain an audit trail of all data changes.
- **Reporting** - to describe and account for accrual, forms entered and completed, etc.
- **Finalization** of a frozen, analytic database from edited or "clean" records.

DATA ANALYSIS

Data analysis for this pilot study (R61 phase) will inform revisions to our upcoming randomized controlled trial (R33 phase). We will conduct a preliminary assessment of our outcome measures, particularly our primary outcomes from the actigraphy TST measurements and fitbit sleep time measurements, and secondary outcomes from the MDS and our supplementary sleep data (see Table 2). We will examine ranges, indicators of variability (change over time), and rates of missing data. We will also examine indicators by demographic characteristics (e.g., age, gender) and level of dementia severity (mild, moderate, severe). To test the sensitivity of our secondary outcomes and supplementary sleep data to individual differences in TST and change in TST, we will preliminarily examine the relationships of these with average TST and variability in TST.

As the study proceeds and procedures are refined and expanded, this section will be elaborated to include specific statistical analysis packages that will be used (e.g., SAS), and more information about analyses.

For the qualitative interviews, all interviews will be audio recorded and transcribed and Dr. McCullough will guide the experienced qualitative team in using a rapid appraisal template analysis,[112,113] a rigorous technique for thematically organizing and analyzing data. The analysis will identify, in particular, areas for modifications to the LOCK sleep program training. Interviews will also inform our decision of whether or not to include Fitbits as part of the subsequent randomized

controlled trial (R33 phase), based on staff members' impressions of the value of Fitbits and their impressions of the feasibility of using both devices. We will also modify interview guides (see Appendix I) for the next phase, as necessary.

19.1 External Data

Definition: External data refers to data sent to or collected at a study organizational component other than a clinical site (e.g., central laboratory, imaging facility, etc.).

Study staff will train NH Leadership teams on proper procedures for collecting and mailing outcome data (e.g., actigraphs at completion of a 7-day measurement period, completed staff ratings of resident sleep global change) to the University of Alabama project office using UA-supplied self-addressed postage paid mailers from a tracked express mailing service (e.g., UPS).

NH staff are provided with subject numbering system including peel-and-stick subject number labels to assure that only subject numbers are included on study forms. In addition, study forms do not contain fields for name or any of the other 15 specific individual identifiers mentioned as those that should not be used (as per the comprehensive list in "Protecting Personal Health Information in Research: Understanding the HIPAA Privacy Rule, NIH Publication 03-5388").

NH staff will email study staff when a package is put in the mail, completing an inventory of what is included in the package, so study staff can begin tracking tracking it and can reconcile the data upon receiving the package. Study staff will complete the tracking log indicating the date each piece of received data is received, entered into the electronic databases, and shared with the data coordinating center.

The actigraph tracking log will be completed indicating that the actigraphs have been received, data downloaded, actigraph storage wiped clean, actigraphy physically cleaned, batteries replaced as indicated by battery replacement schedule, and actigraph re-packaged and re-sent to NH for next use.

19.2 Quality Control Procedures

The leadership team holds quarterly quality assurance meetings in which study binders are reviewed using the IRB audit checklist, tracking logs are reviewed, and data is spot checked.

Tracking logs are maintained for in each of the following areas:
--NH data reconciliation
--Actigraph tracking
--IRB continuing review preparation
--Investigator annual trainings and COI reports
--SAE/AE reports

--NIA quarterly and annual review preparation
--DSMB reports
--Data coordinating center quarterly QA report
--NH enrollment site quarterly QA virtual site visit
--Corporation quarterly QA virtual site visit

19.2.1 Standard Operating Procedures

The following SOPs from the 40Winks Single IRB (The University of Alabama IRB) are significant to the conduct of this clinical trial:
Data and Safety Monitoring in Proposed Research
Federalwide Assurance (FWA) for Protection of Human Subjects
Investigator Assessment of Participant Comprehension
Investigator Responsibility for Informed Consent Process and Documentation
IRB and Investigator Responsibilities for Applications Involving Declared or Undeclared
Investigator Conflicts of Interest
IRB Record Keeping and Management
Modifying Approved IRB Protocols
Protection of Human Research Participants' Privacy and Confidentiality
Protection of the Cognitively Impaired
Qualifications and Responsibilities of Investigators
Reportable Events: Protocol Deviations, Unanticipated Problems, and Adverse Events
Research Utilizing Records, Chart Reviews, and Case Studies/Reports
Review and Oversight of Research Conducted at Multiple Sites
Routine Post-Approval Monitoring of Protocols (PAM)
Sponsored Projects Agreements for Human Subjects Research and Human Research Protections
Subject Recruitment and Compensation
University Expectations for Persons Involved with HRPP
University of Alabama Policy on Conflict of Interest/Financial Disclosures in Research and Other Sponsored Programs
Waivers, Alterations, and Exceptions to Informed Consent or its Written Documentation

19.2.2 Data and Form Checks

The data coordinating center will develop standard operating procedures that include the following data quality control checks to identify potential data anomalies including:

- Missing data or forms
- Out-of-range or erroneous data
- Inconsistent and illogical dates over time
- Data inconsistency across forms and visits
- Not completing all fields of fields on a "completed form" or no reason for missing data is provided

19.2.3 Site Monitoring

The coordinating center will hold quarterly virtual site visits with the data coordinating center, the corporations (i.e., corporate coach) and each active NH enrollment site to assure compliance with study action and documentation procedures. Each site visit will be guided by a checklist. The study staff representative and the site representative will go through the checklist together.

The purposes of monitoring visits are to:

- Ensure the rights and safety of participants
- Confirm that the study is conducted in accordance with GCP guidelines
- Ensure maintenance of required documents
- Verify adherence to the protocol
- Monitor the quality of data collected
- Ensure accurate reporting and documentation of all AEs and unanticipated problems

During monitoring visits, the data recorded on CRFs are reviewed and verified against source documents to ensure:

- Informed consent has been obtained and documented in accordance with IRB/ FDA regulations
- The information recorded on the forms is complete and accurate
- There are no omissions in the reports of specific data elements
- Missing examinations are indicated on the forms
- Participant disposition when exiting the study is accurately recorded

Site representatives will ensure that the monitor has access to all study documents, including informed consent forms, intervention accountability records, and source documents.

Once the site visit is complete, a site monitoring report is drafted to provide feedback regarding any problems or issues that may have been uncovered during the visit. The report should, state the problems uncovered during the visit and describing recommendations to correct them. A timeline will be agreed upon and included in the report to ensure that follow-up of the issues is completed and implemented into the study's procedures.

20.0 Data and Safety Monitoring Activities

This study has a Data and Safety Monitoring Board (DSMB) that is advisory to the NIA Director. The roles and responsibilities of the DSMB are outlined in the DSMB Charter and the Data Safety and Monitoring Plan.

20.1 Reports

The routine reports prepared by the Coordinating Center and Data Center are important quality control tools. Monthly recruitment/intervention reports are produced to describe target and actual enrollment by site and in aggregate, individuals screened with reasons for screen failure, and participant disposition (enrolled, active, completed, discontinued treatment, and lost to follow-up). These reports also list or summarize AEs and SAEs. Monthly administrative reports list the forms completed, entered, and missing and/or erroneous data and forms. In addition to these reports, the DSMB and NIA Program Officer may request additional reports they wish to receive.

An annual continuing review to the UA IRB is required. As the study proceeds and procedures are refined and expanded, this section will be elaborated to include any other reporting requirements

20.2 Study Completion and Close-Out Procedures

As the study proceeds and procedures are refined and expanded this section of the MOP will be updated to briefly outline the study completion and close-out procedures. Details will include the following closeout activities:

- Verification that study procedures have been completed, data have been collected, and study intervention(s) and supplies are returned to the responsible party or prepared for destruction.
- Comparison of the Data Center’s and any individual investigator’s correspondence and study files against the Coordinating Center's records for completeness.
- Assurance that all data queries have been completed.
- Assurance that correspondence and study files are accessible for external audits.
- Reminder to investigators of their ongoing responsibility to maintain study records and to report any relevant study information to the NIA.
- Assurance that the investigator will notify the IRB of the study’s completion and store a copy of the notification.
- Preparation of a report summarizing the study’s conduct.
- Participant notification of the study completion.

As the study proceeds and procedures are refined and expanded a tracking form will be developed to ensure that all of the above receive notification as specified.

20.2.1 Participant Notification

The Coordinating Center will notify each of the three participating NH corporations about completion of the study so they can notify all of their participating NHs and staff. The Coordinating Center will develop a letter to notify participants that the study is completed, ask whether they would like to be informed of the results, and thank them for their participation.

As the study proceeds and procedures are refined and expanded a tracking form will be developed to ensure that all of the above receive notification as specified.

20.2.2 Site Procedures

The coordinating center will provide certificates of appreciation to each enrollment site that completes the study intervention, with additional recognition for sites that exceed recruitment goals, provide high quality data, and ensure excellence in participant retention.

20.2.3 Confidentiality Procedures

SOURCES OF MATERIALS
NH resident data will be collected for consented NH residents from NH staff ratings (global assessments of change in overall sleep quality and sleep-related conditions) and actigraph/Fitbit measurements (wristwatch-sized devices worn on the wrist); medical records (age, diagnoses, medical history, and medications); and MDS assessments (Brief Interview for Mental Status score, psychotropic medication use, pain and analgesic medication use, activities of daily living decline, sleep-related items). NH staff interview data will be collected via audiorecorded qualitative interview. See Table 1 for a listing of all study outcomes and data sources.

PERSONALLY IDENTIFIABLE INFORMATION: ACCESS, MANAGEMENT, AND PROTECTION
The study staff will have access to individually identifiable data about participants. The NH leadership team will have access to individually identifiable data about participants. The flow of individually identifiable data and steps to assure its appropriate access, management, and protection, are outlined below.

1. NH Residents
a. Study staff will successfully obtain all necessary IRB authorizations for protocols and materials described below
b. NH leadership team or their designees will identify all NH residents with an ADRD diagnosis and the NH leadership team will mail to those residents' legally authorized representatives (LARs) an IRB-approved study introduction letter (including option to opt out within one month if they do not wish to be contacted by study staff). Contact information of those LARs not opting out will be shared with University of Alabama (UA) study staff.
c. UA study staff will contact LARs and attempt to gain informed consent for NH resident participation (see 2.a. Informed Consent and Assent). Study staff will weekly provide an updated list of consented residents with study identification numbers to the NH leadership team along with copies of all completed informed consent forms so that a copy can be filed in the resident's medical records and a copy can be stored along with the complete list and other IRB approvals and relevant study information in an essential

documents binder at the NH. These processes will assure that NH staff will be able to easily verify that study staff only have access to personally identifiable data of residents are involved in the study.
d. NH staff will work together in frontline huddles to complete screening of all eligible residents, identify residents with sleep problems to enroll in the LOCK sleep program, and collect assessment data for enrolled residents.
e. Every week, the NH leadership team will use researcher-provided tracked express mail service envelopes to send the UA study staff the following: 1) all completed staff rating forms, identified by NH resident subject number; and 2) any actigraphs and Fitbits for which the assessment periods are complete, identified by subject number. Research staff will mail back actigraphs and Fitbits after data download, deletion of data from the devices, and maintenance.
f. UA study staff will travel to each NH once during the post-intervention period to extract
information from the medical records of consented NH residents (this process and the information to be extracted will be clearly described in IRB-approved protocols and consent forms).
g. UA study staff will upload all data at UA and then transfer to the University of Texas School of Public Health (UTHealth SPH) Data Center via secure file transfer procedures as established by Dr. Morgan and the Data Center.
h. MDS data will be obtained by Dr. Morgan and the UTHealth SPH Data Center through
data use agreement for use of personally identifiable information from CMS. MDS data for consented NH residents will be linked and merged with the other data described above.
i. When data are reported in publication and presentation they will only be reported in aggregate form, and neither NH nor participant will be identifiable.
2. NH Staff for interviews
a. Study staff will successfully obtain all necessary IRB authorizations for protocols and materials described below.
b. NH leadership team will provide to all NH staff an IRB-approved study introduction letter (including option to opt out within one month if they do not wish to be contacted by study staff). Contact information of those NH staff not opting out will be shared with UA study staff.
c. UA Study staff will contact NH staff to invite them to participate in mid-implementation phone interviews. Study staff will also contact NH staff to invite them to participate in post-implementation interviews. The list of NH staff who agree and decline interview participation will remain confidential to protect NH staff from any possible coercion to participate.
d. UA study staff will conduct interviews with consented NH staff. Interviews will be audio
recorded. Recordings will be uploaded at UA, identified only by subject number, and shared with a university-approved transcription service via secure file transfer procedures as established by Dr. Morgan and the UTHealth SPH Data Center.

Transcribed files, identified only by subject number, will be returned via the same secure
file transfer procedures to UA for tracked storage and analysis and to Dr. Hartmann at UMass Lowell for analysis.
e. When data are reported in publication and presentation they will only be reported in aggregate form, and neither NH nor participant will be identifiable.
3. Data Management and Protection. Dr. Morgan and the UTHealth SPH Data Center will create protocols for overseeing the security and protection of all data collected for this study. Personally identifiable information will be only be stored in the secure and securely backed up UA data server and UTHealth SPH Data Center in password protected files. Other collaborating sites (UMass Lowell, Brown University, and University of Texas at Austin) will only work with data identified by subject number.

As the study proceeds and procedures are refined and expanded, this section will be elaborated to include all safeguards which have been put in place by the Steering Committee to ensure participant confidentiality and data security. The leadership committee and steering committee will outline and enforce participant and study data confidentiality policies. Study staff will be instructed in their responsibilities regarding data safeguards and cautioned against the release of data to any unauthorized individuals, unless such a release is approved by the study leadership and NIA and is not in violation of applicable Federal and state laws.

When complete this section will include all of these study participant confidentiality safeguards:

- ***Data flow procedures*** – data identifying participants should not be transmitted from study sites to the Coordinating Center.
- ***Electronic files*** – data identifying participants that are stored electronically should be maintained in an encrypted form or in a separate file.
- ***Forms*** - forms or pages containing personal identifying information should be separated from other pages of the data forms and retained in a secure location.
- ***Data listings*** - participant name, name code, hospital chart, record number, Social Security Number, or other unique identifiers should not be included in any published data listing.
- ***Data distribution*** - data listings that contain participant name, name code, or other identifiers easily associated with a specific participant should not be distributed.
- ***Data disposal*** - computer listings that contain participant-identifying information should be disposed of in an appropriate manner.
- ***Access*** - participant records should not be accessible to persons outside the study without the express written consent of the participant.
- ***Storage*** - study forms and related documents retained both during and after study completion should be stored in a secure location.

Computers are used to store and/or analyze clinical data. The Coordinating Center and Data Center will address elements of computer security in this section to ensure that the data remain confidential. These elements include but are not limited to: utilization of computer and system passwords, user security training, system training and verification, and routine system backups to prevent any loss of electronic data.

20.2.4 Publications

As the study proceeds and procedures are refined and expanded a publication policy will be developed that ensures that data are not released inappropriately, authorship is predetermined, and manuscripts are subjected to rigorous review before they are submitted for publication.

The coordinating center will assure that basic results of the trial are reported in ClinicalTrials.gov within 12 months of trial completion, as required by federal policies.

21.0 MOP Maintenance

This MOP will be continuously updated by study staff under the oversight of the Coordinating Center and reviewed during its quarterly quality assurance review to ensure that the operating procedures described are accurate. This MOP is maintained electronically in a shared drive accessible to all study investigators and staff across all study sites. Each page of the MOP is numbered, dated, and contains a version number to facilitate any changes and/or additions. The MOP serves as a history of the project, documenting the time and nature of any changes in procedures and policies.

The MOP template for changes is included in Appendix E.

Bibliography

For additional information, please refer to the resources listed below. Numbering corresponds to numbering used in this document.

General Clinical Trial
Blumenstein BA, James KE, Lind BK, Mitchell HE. Functions and Organization of Coordinating Centers for Multicenter Studies. Controlled Clinical Trials 1995;16:4S-29S.
Bucher HC, Guyatt GH, Cook, DJ, Holbrook A, McAlister FA. Users Guide to the Medical Literature. JAMA 1999;282(8):771-778.

Friedman LM, Furberg CD, DeMets DL. Fundamentals of Clinical Trials. Mosby, Baltimore: 1996.

Senturia YD, Mortimer KM, Baker D, Gergen P, Mitchell H, Joseph C, Wedner J. Successful Techniques for Retention of Study Participants in an Inner-City Population. Controlled Clinical Trials 1998;19:544-554.

Statistical Analysis
Huster W, Shah A, Kaiser G, Dere W, DiMarchi R. Statistical and Operational Issues Arising in an Interim Analysis When the Study Will Continue. Drug Information Journal 1999;33:869-875.

Meinert CL. Clinical Trials: Design, Conduct, and Analysis. Oxford University Press, New York: 1986.

112.King N. Using templates in the thematic analysis of text. In: Essential Guide to Qualitative Methods in Organizational Research. London: SAGE Publications Ltd; 2004.

113.Beebe J. Basic Concepts and Techniques of Rapid Appraisal. Human Organization. 1995;54(1):42-51.

Monitoring, Quality Assurance and Adverse Event Reporting
Bohaychuk W, Ball G, Lawrence G, Sotirov K. Good Clinical Practice: Data Integrity Needs Upgrading. Applied Clinical Trials 1999(January):54-61.

Knatterud GL, Rockhold FW, George SL, Barton FB, Davis CE, Fairweather WR, Honohan T, Mowery R, O-Neill R. Guidelines for Quality Assurance in Multicenter Trials: A Position Paper. Controlled Clinical Trials 1998;19:477-493.van der Putten E, van der Velden JW, Siers A, Hamersma EAM, for the Cooperative Study Group of Dutch Datamanagers. A pilot Study on the Quality of Data Management in a Cancer Clinical Trial. Controlled Clinical Trials 1987;8:96-100.

Wittes J. Behind Closed Doors: The Data Monitoring Board in Randomized Clinical Trials. Statistics in Medicine 1993;12:419-424.

Intervention and Measurement

25.Brady C, Farrell D, Frank B, Elliot AE, Gittell JH. A long-term care leader's guide to high performance : doing better together. Baltimore: Health Professions Press;2018.
35.Farrell D, Brady C, Frank B. Meeting the leadership challenge in long-term care : what you do matters. Baltimore: Health Professions Press; 2011.
37.Franzmann J, Haberstroh J, Pantel J. Train the trainer in dementia care. A program to foster communication skills in nursing home staff caring for dementia patients. Z Gerontol Geriatr. 2016;49(3):209-215.
38.Mayrhofer A, Goodman C, Smeeton N, Handley M, Amador S, Davies S. The feasibility of a train-the-trainer approach to end of life care training in care homes: an evaluation. BMC Palliat Care. 2016;15:11.
39.Sampson EL, Vickerstaff V, Lietz S, Orrell M. Improving the care of people with dementia in general hospitals: evaluation of a whole-system train-the-trainer model. Int Psychogeriatr. 2017;29(4):605-614.
70.de Zambotti M, Goldstone A, Claudatos S, Colrain IM, Baker FC. A validation study of Fitbit Charge 2 compared with polysomnography in adults. Chronobiology international. 2018;35(4):465-476.
71.Kolla BP, Mansukhani S, Mansukhani MP. Consumer sleep tracking devices: a review of mechanisms, validity and utility. Expert review of medical devices. 2016;13(5):497-506.
81.Colon-Emeric C, Toles M, Cary MP, Jr., et al. Sustaining complex interventions in long-term care: a qualitative study of direct care staff and managers. Implementation science : IS. 2016;11:94.
82.Chung F, Subramanyam R, Liao P, Sasaki E, Shapiro C, Sun Y. High STOPBang score indicates a high probability of obstructive sleep apnoea. British journal of anaesthesia. 2012;108(5):768-775.
83.Chung F, Yang Y, Brown R, Liao P. Alternative scoring models of STOP-bang questionnaire improve specificity to detect undiagnosed obstructive sleep apnea. Journal of clinical sleep medicine : JCSM : official publication of the American Academy of Sleep Medicine. 2014;10(9):951-958.
84.Chung F, Yegneswaran B, Liao P, et al. STOP questionnaire: a tool to screen patients for obstructive sleep apnea. Anesthesiology. 2008;108(5):812-821.
85.Smith MT, McCrae CS, Cheung J, et al. Use of Actigraphy for the Evaluation of Sleep Disorders and Circadian Rhythm Sleep-Wake Disorders: An American Academy of Sleep Medicine Systematic Review, Meta-Analysis, and GRADE Assessment. Journal of clinical sleep medicine : JCSM : official publication of the American Academy of Sleep Medicine. 2018;14(7):1209-1230.
86.Maglione JE, Liu L, Neikrug AB, et al. Actigraphy for the assessment of sleep measures in Parkinson's disease. Sleep. 2013;36(8):1209-1217.
87.Bellone GJ, Plano SA, Cardinali DP, Chada DP, Vigo DE, Golombek DA. Comparative analysis of actigraphy performance in healthy young subjects. Sleep science (Sao Paulo, Brazil). 2016;9(4):272-279.

88.Marino M, Li Y, Rueschman MN, et al. Measuring sleep: accuracy, sensitivity, and specificity of wrist actigraphy compared to polysomnography. Sleep. 2013;36(11):1747-1755.
97.Berk M, Ng F, Dodd S, et al. The validity of the CGI severity and improvement scales as measures of clinical effectiveness suitable for routine clinical use. Journal of evaluation in clinical practice. 2008;14(6):979-983.
98.Black JE, Hull SG, Tiller J, Yang R, Harsh JR. The long-term tolerability and efficacy of armodafinil in patients with excessive sleepiness associated with treated obstructive sleep apnea, shift work disorder, or narcolepsy: an open-label extension study. Journal of clinical sleep medicine : JCSM : official publication of the American Academy of Sleep Medicine. 2010;6(5):458-466.
99.Busner J, Targum SD. The clinical global impressions scale: applying a research tool in clinical practice. Psychiatry (Edgmont (Pa : Township)). 2007;4(7):28-37.
100.Dunlop BW, Gray J, Rapaport MH. Transdiagnostic Clinical Global Impression Scoring for Routine Clinical Settings. Behavioral sciences (Basel, Switzerland). 2017;7(3).
101.Dworkin RH, Turk DC, Farrar JT, et al. Core outcome measures for chronic pain clinical trials: IMMPACT recommendations. Pain. 2005;113(1-2):9-19.
102.Farrar JT, Young JP, Jr., LaMoreaux L, Werth JL, Poole RM. Clinical importance of changes in chronic pain intensity measured on an 11-point numerical pain rating scale. Pain. 2001;94(2):149-158.
103.Schneider LS, Olin JT, Doody RS, et al. Validity and reliability of the Alzheimer's Disease Cooperative Study-Clinical Global Impression of Change. The Alzheimer's Disease Cooperative Study. Alzheimer disease and associated disorders. 1997;11 Suppl 2:S22-32.
104.Teri L, Logsdon RG, Peskind E, et al. Treatment of agitation in AD: a randomized, placebo-controlled clinical trial. Neurology. 2000;55(9):1271-1278.
105.Boland J, Currow DC, Wilcock A, et al. A systematic review of strategies used to increase recruitment of people with cancer or organ failure into clinical trials: implications for palliative care research. Journal of pain and symptom management. 2015;49(4):762-772.e765.
106.Hunt KJ, Shlomo N, Addington-Hall J. Participant recruitment in sensitive surveys: a comparative trial of 'opt in' versus 'opt out' approaches. BMC medical research methodology. 2013;13:3.
107.Miller CJ, Burgess JF, Jr., Fischer EP, et al. Practical application of opt-out recruitment methods in two health services research studies. BMC medical research methodology. 2017;17(1):57.
108.Vellinga A, Cormican M, Hanahoe B, Bennett K, Murphy AW. Opt-out as an acceptable method of obtaining consent in medical research: a short report. BMC medical research methodology. 2011;11:40.
109.Richards K, Shue VM, Beck CK, Lambert CW, Bliwise DL. Restless legs syndrome risk factors, behaviors, and diagnoses in persons with early to moderate dementia and sleep disturbance. Behavioral sleep medicine. 2010;8(1):48-61.
110.Richards KC, Lambert C, Beck CK, et al. Strength training, walking, and social

activity improve sleep in nursing home and assisted living residents: randomized controlled trial. J Am Geriatr Soc. 2011;59(2):214-223.
111.Rose KM, Beck C, Tsai PF, et al. Sleep disturbances and nocturnal agitation behaviors in older adults with dementia. Sleep. 2011;34(6):779-786.

RELEVANT WEB SITES

Food and Drug Administration:

http://www.fda.gov/cber/guidelines.htm

http://www.fda.gov/ora/compliance_ref/part11/

http://www.accessdata.fda.gov/scripts/cdrh/cfdocs/cfcfr/cfrsearch.cfm

Gene Therapy, Stem Cells and Fetal Tissue

http://grants.nih.gov/grants/policy/gene_therapy_20000307.htm

http://grants.nih.gov/grants/guide/notice-files/NOT-OD-00-050.html

http://grants.nih.gov/grants/guide/notice-files/NOT-OD-00-026.html

Information Required in NIH Grant Applications:

http://grants.nih.gov/grants/policy/policy.htm

NIH Policies for Monitoring Clinical Research:

http://grants.nih.gov/grants/guide/notice-files/not99-044.html

http://grants.nih.gov/grants/guide/notice-files/not98-084.html

http://grants.nih.gov/grants/guide/notice-files/NOT-OD-00-038.html

http://grants.nih.gov/grants/guide/notice-files/not99-107.html

http://grants.nih.gov/grants/guide/notice-files/NOT-OD-00-053.html

Implementation of NIA Policies for Human Intervention Studies
http://www.nia.nih.gov/research/dea/implementation-policies-human-intervention-studies

Guidelines for Writing Informed Consent Documents
http://www.hhs.gov/ohrp/policy/ictips.html

University of Alabama IRB
http://ovpred.ua.edu/research-compliance/institutional-review-board-irb/

NIA Clinical Research Study Investigator's Toolbox
https://www.nia.nih.gov/research/clinical-research-study-investigators-toolbox?utm_source=staticct&utm_medium=staticct&utm_campaign=staticct#startup

APPENDIX A - ACRONYM GLOSSARY

Adverse Event (AE) – Any untoward or unfavorable medical occurrence in a clinical research study participant, including any abnormal sign (e.g. abnormal physical exam or laboratory finding), symptom, or disease, temporally associated with the participants' involvement in the research, whether or not considered related to participation in the research.

Case Report Form (CRF) – A printed, optical, or electronic (eCRF) document designed to capture all protocol-required information for a study (i.e., data collection forms, study measure forms).

Code of Federal Regulations (CFR) - is an annual codification of the general and permanent rules published in the Federal Register by the executive departments and agencies of the Federal Government.

Coordinating Center (CC) – A group organized to coordinate the planning and operational aspects of a multi-center clinical trial. CCs may also be referred to as Data Coordinating Centers (DCCs) or Data Management Centers (DMCs).

Data and Safety Monitoring Board (DSMB) –A group of individuals independent of the study investigators that is appointed by the NIA to monitor participant safety, data quality and to assess clinical trial progress.

Data Coordinating Center (or Data Center) (DC) – The group organized to oversee data management and analysis.

Food and Drug Administration (FDA) – An agency within the U.S. Department of Health and Human Services (DHHS) responsible for protecting the public health by assuring the safety, efficacy, and security of human and veterinary drugs, biological products, medical devices, nation's food supply, cosmetics, and products that emit radiation.

Forty Winks (abbreviated 40Winks) – **F**rontline-staff **O**pen, **R**elationally-coordinated **T**eams **Y**ielding **W**orking **I**nitiatives to **N**urture and **K**indle **S**leep. Informal nickname for this study.

Good Clinical Practice (GCP) – A standard for the design, conduct, performance, monitoring, auditing, recording, analyses, and reporting of clinical trials that provides assurance that the data and reported results are credible and accurate, and that the rights, integrity, and confidentiality of trial participants are protected.

Health Insurance Portability and Accountability Act (HIPAA) Privacy Rule – The first comprehensive Federal protection for the privacy of personal health information. The Privacy Rule regulates the way certain health care groups, organizations, or

businesses, called covered entities under the Rule, handle the individually identifiable health information known as protected health information (PHI).

Institutional Review Board (IRB)/Independent Ethics Committee (IEC) – An independent body constituted of medical, scientific, and nonscientific members whose responsibility it is to ensure the protection of the rights, safety, and well-being of human subjects involved in a trial by, among other things, reviewing, approving, and providing continuing review of trials, protocols and amendments, and of the methods and material to be used to obtaining and documenting informed consent of the trial participant.

Manual of Procedures (MOP) – A “cook book” that translates the protocol into a set of operational procedures to guide study conduct. A MOP is developed to facilitate consistency in protocol implementation and data collection across study participants and clinical sites.

Nursing Home (NH) – the skilled nursing facilities serving as enrollment/intervention sites

Principal Investigator (PI) and co-Principal Investigators (coPIs)- The individuals with primary responsibility for achieving the technical success of the project, while also complying with the financial and administrative policies and regulations associated with the award. Although Principal Investigators may have administrative staff to assist them with the management of project funds, the ultimate responsibility for the management of the sponsored research award rests with the Principal Investigator.

Quality Control (QC) – The internal operational techniques and activities undertaken within the quality assurance system to verify that the requirements for quality of trial related activities have been fulfilled (e.g., data and form checks, monitoring by study staff, routine reports, correction actions, etc.)

Safety Officer (SO) - The Safety Officer is an independent individual, usually a clinician, who performs data and safety monitoring activities in low-risk, single site clinical studies. The Safety Officer advises NIA Program Director regarding participant safety, scientific integrity and ethical conduct of a study.

Serious Adverse Event (SAE) – Any adverse event that:

- Results in death
- Is life threatening, or places the participant at immediate risk of death from the event as it occurred
- Requires or prolongs hospitalization
- Causes persistent or significant disability or incapacity
- Results in congenital anomalies or birth defects
- Is another condition which investigators judge to represent significant hazards

Standard Operating Procedure (SOPs) – Detailed written instructions to achieve uniformity of the performance of a specific function across studies and patients at an individual site.

Appendix B - Screening Log

Study: 40 Winks *(Enhancing Sleep Quality for NH Residents w Dementia)*
Site: [Site Name]
Investigator: A. Lynn Snow, PhD - The University of Alabama

Screening Number	Date of Birth	Gender	Screening Date	Screening Status (use codes below)	Consent Obtained	Enrolled (if no, indicate reason from codes below)	Date Enrolled
☐☐☐☐	/ / mm/dd/yyyy	☐ M ☐ F	/ / mm/dd/yyyy		☐ Yes ☐ No	☐ Yes ☐ No	/ / mm/dd/yyyy
☐☐☐☐	/ / mm/dd/yyyy	☐ M ☐ F	/ / mm/dd/yyyy		☐ Yes ☐ No	☐ Yes ☐ No	/ / mm/dd/yyyy
☐☐☐☐	/ / mm/dd/yyyy	☐ M ☐ F	/ / mm/dd/yyyy		☐ Yes ☐ No	☐ Yes ☐ No	/ / mm/dd/yyyy
☐☐☐☐	/ / mm/dd/yyyy	☐ M ☐ F	/ / mm/dd/yyyy		☐ Yes ☐ No	☐ Yes ☐ No	/ / mm/dd/yyyy
☐☐☐☐	/ / mm/dd/yyyy	☐ M ☐ F	/ / mm/dd/yyyy		☐ Yes ☐ No	☐ Yes ☐ No	/ / mm/dd/yyyy

Sample Screen Status Codes:
1-Eligible
2-Eligible, declined participation
3-Not Eligible
4-Eligible, lost to follow-up
5-Other, specify in space provided

If not eligible, Reason:
1-Inclusion # (specify)
2-Exclusion# (specify)
3-Other (specify)

Appendix C - Sample Schedule of Events

Visit Description	Screening	*TP	*TP	*TP	*TP	*TP	*TP	*TP	**FU	**FU	**FU	**FU	**FU	**FU
Study Visits/ Study days (or weeks)	Visit-1 Day-14 to Day -1	Visit 1 Day 0	2 W1	3 W2	4 W3	5 W4	6 W8	Final Visit W10	8 W12	9 W14	10 W16	11 W18	12 W20	13 W22
Informed Consent	X													
12-lead EKG	X				X			X	X					X
Medical History	X													
Prior Medications	X													
Physical Exam	X							X						
Vital Signs	X							X						
Chemistries	X		X	X	X			X	X					X
Liver Function Tests	X		X	X	X			X	X					X
Hematology	X		X	X	X			X	X					X
Pregnancy Test	X				X			X	X					X
Investigational Agent Administration		X	X	X	X	X	X	X						
Concomitant Medications		X	X	X	X	X	X	X	X	X	X	X	X	X
Adverse Events		X	X	X	X	X	X	X	X	X	X	X	X	X
Study completion														X

*TP – Treatment Phase

**FU – Follow-up Phase

Appendix D - Administrative Forms

An Administrative Form constitutes any form that would not be included in the study database. The following is this study's list of administrative forms.

Participant Identification Code List - Used to document the participant's study identification number, name, and other identifying information. Must be stored securely and separate from research records since it is the link between a study ID and participant's name.

Screening and Enrollment Log - Used to list participants screened; includes those who fail screening and those who are enrolled.

Site-Signature Log /Delegation of Authority Log* - Used to list all study personnel and their specific responsibilities, signatures, and dates of obligation during the conduct of a clinical research study

Site Visit Log - Records individuals visiting the site. The most common reasons for visits are site initiation, monitoring, training, and close-out.

Telephone Contact Log - To record and track study-related telephone contact discussions with a study participant.

Training Log* - Documents study-specific training completed by staff exhibiting their qualifications to perform tasks involved in the clinical research study. Other training may also be listed on this log.

*This form is a regulatory document as well as an administrative form.

Appendix E - MOP Modification Log

MOP MODIFICATION LOG

Section #	Version #	Date Modified	Page #	Text Location	Brief Modification Summary

APPENDIX F - STUDY ABSTRACT

Disturbed sleep places older adults at higher risk for frailty, morbidity, and even mortality. Yet nursing home (NH) routines frequently disturb residents' sleep through use of noise and light or efforts, for example, to reduce incontinence. NH residents with Alzheimer's disease or related dementias (ADRD)—almost two-thirds of long-stay NH residents—are likely to be particularly affected by sleep disturbance. This study tackles these important issues and substantially moves forward goals of the National Plan to Address Alzheimer's Disease 2018 Update by proposing to implement an evidence-based intervention to improve sleep: a NH frontline staff huddling program known as LOCK. The LOCK program is derived from evidence supporting strengths-based learning, systematic observation, relationship-based teamwork, and efficiency. A LOCK program pilot targeting staff-resident interactions enabled 6 Veterans Health Administration (VA) NHs make meaningful quantitative and qualitative improvements. Preliminary qualitative data from a national VA roll out show that the program is effective when targeting resident clinical outcomes such as pain and pressure ulcers. The program's methods have also improved clinical care in non-VA NHs. This proposal is therefore an NIH Stage III, real world hybrid efficacy-effectiveness pragmatic trial of the LOCK program for sleep (LOCK sleep program).

In non-VA NHs from 3 NH corporations, the study will (1) refine the LOCK program to focus on sleep for residents with ADRD in an R61 phase, (2) test the impact of the LOCK sleep program for NH residents with ADRD in an R33 phase, and (3) evaluate the program's sustainability. **The R61 phase (1 year; n = 3 NHs; 1 NH per corporation) has the following specific aims**: (1) Refine the LOCK sleep program train-the-trainer protocol by implementing and pilot-testing it. (2) Refine the research methods to effectively identify eligible NHs and residents, obtain consent, collect primary data from residents and staff, explore staff impressions of additional sleep measurement devices (Fitbits), transfer primary and secondary data to our data center, and merge all data.

After successfully completing designated R61 milestones and refining the intervention methods, we will conduct a wedge-design randomized, controlled trial. **The R33 phase (4 years; n = 24 NHs; 8 NHs per corporation) has the following specific aims**: (1) Implement the LOCK-based sleep program for residents with ADRD using the train-the-trainer model. (2) Estimate impact of the LOCK sleep program on sleep (primary outcome) and on psychotropic medication use, pain and analgesic medication use, and activities of daily living decline (secondary outcomes). (3) Examine factors, using mixed methods, associated with variation in the program's implementation and its sustainability. This innovative program also has future potential to address other important issues (safety, infection control) and expand to other settings (assisted living, inpatient mental health). The study's strong team, careful consideration of design challenges, and resulting rigorous, pragmatic approach will ensure success of this promising intervention for NH residents with ADRD.

Appendix G – UA IRB Protocol

Personnel Information 1

Subject Population 4

Study Location 4

General Checklist 5

Funding 5

Expedited Review 5

Background , Purpose , Study Procedures 7

Radioisotopes or Radiation Machines 25

Drugs, Reagents, Chemicals, or Biologic Products 26

Devices 26

Subject Population(a-h) 26

Subject Population(i-l) 27

Subject Population(m) 28

Subject Population(n) 31

Subject Population(o) 37

Subject Population(p) 40

Risks 43

Benefits/Alternatives, Procedures to Maintain Confidentiality 55

Potential Conflict of Interest 58

Informed Consent 60

Assent 63

HIPAA 63

Attachments 65

PI Obligations 67

Event History 68

**Protocol Title:**	Enhancing Sleep Quality for Nursing Home Residents with Dementia: Pragmatic Trial of an Evidence-Based Frontline Huddling Program (Pilot Phase-R61)
**Protocol Status:**	Comments Received (Cycle 1)
**Date Submitted:**	10/16/2019
**Approval Period:**	Draft
**Important Note:**	This Print View may not reflect all comments and contingencies for approval. Please check the comments section of the online protocol. Questions that appear to not have been answered may not have been required for this submission. Please see the system application for more details.

* * * Personnel Information * * *

Study Personnel Roles:
-Principal Investigator: accepts responsibility for study, can edit protocol, must submit to IRB
-Administrative Contact: additional study contact, can edit/prepare protocol, may or may not also be member of research team
-Key Personnel (Research Team): University of Alabama member of research team, can view protocol (not edit)
-Non-Alabama Collaborator: member of research team from another institution or organization outside of University of Alabama, has no access to system, must be provided with PDF of protocol.
-Department Chair: Official Department Chair, may or may not also be a member of research team, can view the protocol (not edit). NOTE: a proxy may be listed if the Chair is the PI.

IMPORTANT NOTE: Human Participants Protection Training is mandatory for all research team personnel.

Principal Investigator Mandatory

PI must be University of Alabama affiliate.

**Name of Principal Investigator (Faculty, Staff or Student)**	**Degree (MD/PhD/Other)**	**Title**
Snow, Andrea	PhD	Professor
**Email**	**Phone**	**Fax**
LSNOW@ua.edu	+1 205 348 7518	+1 205 348 7520

Department Name
Psychology

Please indicate your status Faculty

Is the (Role) also a Department Chair? N

Human Subjects Training Completed? Y

If you have completed training that is not auto-populated below, upload a copy in the Attachments section.

Research Team Member Duties Picklist

1.	X	Recruitment	2.	X	Obtains consent
3.	X	Determine participant Eligibility for Accrual	4a.		Participant Physical Examinations
4b.		Follow-up Visits including physical assessments	5.	X	Perform study procedures or Specimen Collection

6a.		Administer and/or Dispense Study Drugs, Biologics or Devices	6b.		Receive, Store, Manipulate or Account for Study Drugs, Biologics or Devices
7.	X	Participant Randomization or Registry	8.	X	Collection of Participant Data
9.	X	Report Data (CRFs, e-CRFs, Spreadsheets)	10.	X	Data Analysis
11a.	X	Review Adverse Events	11b.		Treat and Classify Adverse Events
12.		Other (Please insert explanation below.)			

No training data is available.

Administrative Contact

Name of Administrative Contact.	Degree (MD/PhD/Other)	Title	Department Name
Cox, Brian	MS	Research Project Coord PT	Ctr for Mental Health & Aging-CMHA

Key Personnel (Research Team)

Name of Key Personnel (Research Team)	Degree (MD/PhD/Other)	Title	Department Name
Parmelee, Patricia	PhD	Director / Professor	Alabama Research Institute on Aging / Psychology

Non - Alabama Collaborator

Name of Non - Alabama Collaborator	Degree (MD/PhD/Other)	Title	Department Name
Christine Hartmann	PhD	Associate Professor, UMass Lowell	Other
Kathy Richards	PhD MSN BSN	Professor, University of Texas School of Nursing	Other
Liam Fry	MD	Professor, Dell Medical School, University of Texas	Other
Robert Morgan	PhD	Professor, University of Texas School of Public Health	Other
Megan McCullough	PhD	Research Assistant Professor, UMass Lowell	Other
Rosa Baier	PhD	Associate Director, Brown Center for Long-Term Care Quality & Innovation	Other
Ellen McCreedy	PhD	Assistant Professor, Brown University	Other
Barbara Frank	MPA	Co-Founder, B&F Consulting	Other
Cathie Brady	MS	Co-founder, B&F Consulting	Other

Department Chair Mandatory

The official Department Chair should be listed here. If the Department Chair is the PI, a proxy may be listed.

Name of Department Chair	Degree (MD/PhD/Other)	Title
Conners, Frances	PhD	Professor and Chair
**Email**	**Phone**	**Fax**
fconners@ua.edu	+1 205 348 7913	+1 205 348 8648

Department Name

Psychology

Human Subjects Training Completed? Y

If you have completed training that is not auto-populated below, upload a copy in the Attachments section.

Is Chair a member of the study team? N

Research Team Member Duties Picklist

1. **Recruitment**
2. **Obtains consent**

3.	Determine Participant Eligibility for Accrual	4a.	Participant Physical Examinations
4b.	Follow-up Visits including physical assessments	5.	Perform study procedures or Specimen Collection
6a.	Administer and/or Dispense Study Drugs, Biologics or Devices	6b.	Receive, Store, Manipulate or Account for Study Drugs, Biologics or Devices
7.	Participant Randomization or Registry	8.	Collection of Participant Data
9.	Report Data (CRFs, e-CRFs, Spreadsheets)	10.	Data Analysis
11a.	Review Adverse Events	11b.	Treat and Classify Adverse Events
12.	Other (Please insert explanation below.)		

No training data is available.

----

* * * Subject Population * * *

Subject Population(s) Checklist

Select All That Apply:

X Adult Volunteers

X Cognitively Impaired Participants

X Employees

Fetuses

Minors (under 18)

Pregnant Women

Prisoners

Students (Note: If students will be compensated extra-credit or course credit for participation in the research, they must be given a non-research alternative for obtaining the same amount of credit, which is of comparable time and effort as is required by the research activity.)

Terminally Ill Participants

Wards of the State (Note: Please consider whether the research population may also be considered "prisoners" or "cognitively impaired." If so, please mark the appropriate corresponding categories in the Subject Population Checklist)

Non-English Speakers (Note: Please provide copies of all correspondence that will be used as a part of the research in English as well as in the native language of participants. Please also attach a copy of the Translator's Declaration.)

Other (any population that is not specified above)

----

* * * Study Location * * *

Study Location(s) Checklist

Indicate where the study will be conducted. Select all that apply:

X The University of Alabama

Another University or College

VA Center

Hospital

X Other: Community Nursing Homes

X Community Nursing Homes (to be named)

---

* * * General Checklist * * *

General Checklist

Select All That Apply :

- Study Eligible for Exempt Review
- Non-human participants research
- Collection of Specimens
- Data collection via e-mail or the Internet
- FDA Approved Device
- FDA approved drugs, reagents, other chemicals administered to participants (even if they are not being studied), or biologic products
- Genetic Testing
- HIV Testing
- Human blood, cells, tissues, or body fluids
- Investigational drugs, reagents, chemicals, or biologic products
- Investigational Device
- X Investigator Initiated Study
- X Medical Records
- X Photography, Video, or Voice-Recording Participants
- X Questionnaires and/or tests
- Radioisotopes/radiation-producing machines, even if standard of care
- rDNA/Gene Transfer Therapy
- Registry or Repository Creation
- Specimens to be stored for future research projects (must be in consent form)
- Study of existing data or specimens
- Other (clarify in text box to the right)

---

* * * Funding * * *

Funding Checklist

NONE

Funding - Grants/Contracts

Funding Type	Funded By
Government	National Institute of Health

NOTE: Applicable grant application, contract or subcontract, investigator's brochure, and sponsor's protocol (for all industry sponsored clinical trials) must be attached. Click "Add" to attach the documents.

---

* * * Expedited Review * * *

To request an Expedited Review, check the appropriate category(ies) below. Provide justification for your request for Expedited Review.

To qualify for expedited review, research activities must (1) present no more than minimal risk to human subjects, and (2) involve only procedures listed in one or more of the categories below.

Select one or more of the following paragraph(s):

1. **Clinical studies of drugs and medical devices only when condition (a) or (b) is met.**
   a) **Research on drugs for which an investigational new drug application (21 CFR Part 31, 32) is not required. (Note: Research on marketed drugs that significantly increases the risks or decreases the acceptability of the risks associated with the use of the product is not eligible for expedited review.)**
   b) **Research on medical devices for which**
      i) **An investigational device exemption application (21 CFR Part 812) is not required; or**
      ii) **The medical device is cleared/approved for marketing and the medical device is being used in accordance with its cleared/approved labeling.**

2. **Collection of blood samples by finger stick, heel stick, ear stick, or venipuncture as follows:**
   a) **From healthy, nonpregnant adults who weigh at least 110 pounds. For these participants, the amounts drawn may not exceed 550 ml in an 8-week period and collection may not occur more frequently than 2 times per week; or**
   b) **From other adults and children, considering the age, weight, and health of the participants, the collection procedure, the amount of blood to be collected, and the frequency with which it will be collected. For these participants, the amount drawn may not exceed the lesser of 50 ml or 3 ml per kg in an 8-week period and collection may not occur more frequently than 2 times per week.**

   Children are "persons who have not attained the legal age for consent to treatments or procedures involved in the research, under the applicable law of the jurisdiction in which the research will be conducted."

3. **Prospective collection of biological specimens for research purposes by non-invasive means.**

   EXAMPLES: (a) hair and nail clippings in a nondisfiguring manner; (b) deciduous teeth at time of exfoliation or if routine patient care indicates a need for extraction; (c) permanent teeth if routine patient care indicates a need for extraction; (d) excreta and external secretions (including sweat); (e) uncannulated saliva collected either in an unstimulated fashion or stimulated by chewing gumbase or wax or by applying a dilute citric solution to the tongue; (f) placenta removed at delivery; (g) amniotic fluid obtained at the time of rupture of the membrane prior to or during labor; (h) supra-and subgingival dental plaque and calculus, provided the collection procedure is not more invasive than routine prophylactic scaling of the teeth and the process is accomplished in accordance with accepted prophylactic techniques; (i) mucosal and skin cells collected by buccal scraping or swab, skin swab, or mouth washings; (j) sputum collected after saline mist nebulization.

4. **Collection of data through non-invasive procedures (not involving general anesthesia or sedation) routinely employed in clinical practice, excluding procedures involving X-rays or microwaves. Where medical devices are employed, they must be cleared/approved for marketing. (Studies intended to evaluate the safety and effectiveness of the medical device are not generally eligible for expedited review, including studies of cleared medical devices for new indications.)**

   EXAMPLES: (a) physical sensors that are applied either to the surface of the body or at a distance and do not involve input of significant amounts of energy into the participant or an invasion of the participants' privacy; (b) weighing or testing sensory acuity; (c) magnetic resonance imaging; (d) electrocardiography, electroencephalography, thermography, detection of naturally occurring radioactivity, electroretinography, ultrasound, diagnostic infrared imaging, doppler blood flow, and echocardiology; (e) moderate exercise, muscular strength testing, body composition assessment, and flexibility testing where appropriate given the age, weight and health of the individual.

5. Research involving materials (data, documents, records, or specimens) that have been collected, or will be collected solely for nonresearch purposes (such as medical treatment or diagnosis). (NOTE: Some research in this category may be exempt from the HHS regulations for the protection of human participants. 45CFR 46.101(b)(4). This listing refers only to research that is not exempt.)

6. Collection of data from voice, video, digital, or image recordings made for research purposes. This category should only be selected if the present research will involve analysis of data that has been previously recorded.

7. Research on individual or group characteristics or behavior (including, but not limited to, research on perception, cognition, motivation, identity, language, communication, cultural beliefs or practices, and social behavior) or research employing survey, interview, oral history, focus group, program evaluation, human factors evaluation, or quality assurance methodologies. (NOTE: Some research in this category may be exempt from the HHS regulations for the protection of human participants. 45 CFR 46.101(b)(2) and (b)(3). This listing refers only to research that is not exempt.)

8. [FOR IRB use only]. Continuing review of research previously approved by a convened IRB only when condition (a), (b), or (c) is met.
   a) Previously approved research where
      (i) The research is permanently closed to the enrollment of new participants;
      (ii) All participants have completed all research-related interventions; and
      (iii) The research remains active only for the long term follow-up of participants.
   b) Previously approved research where no participants have been enrolled and no additional risks have been identified.
   c) Previously approved research where the remaining research activities are limited to data analysis.

9. [FOR IRB use only]. Continuing review or research not conducted under an investigational new drug application or investigational drug exemption where expedited categories two (2) through eight (8) do not apply but the IRB has determined and documented at a convened meeting that the research involves no greater than minimal risk and no additional risks have been identified.

----

* * * Background , Purpose , Study Procedures * * *

Study Title

Enhancing Sleep Quality for Nursing Home Residents with Dementia: Pragmatic Trial of an Evidence-Based Frontline Huddling Program (Pilot Phase-R61)

Complete Sections 1 - 15. Specify N/A as appropriate. Do not leave any required sections blank.

1) Background

a) **Describe past experimental and/or clinical findings leading to the formulation of the study, if applicable.**

A layman's case for the benefits of a good night's sleep is easy to make: imagine someone woke you last night every two hours. How do you feel? Now imagine that routine continues every night for the next month. For many residents of nursing homes (NHs), such awakening is standard practice.

NHs implement such routines to address various challenges (e.g, incontinence), even though evidence from both objective and subjective measures identifies disturbed sleep as a key contributor to many types of physical, emotional, and cognitive decline.[1-4] Disturbed sleep places individuals at higher risk for frailty,

cognitive decline.[1-4] Disturbed sleep places individuals at higher risk for frailty, morbidity, and even mortality.[5-10] And individuals with Alzheimer's disease or related dementias (ADRD)—almost two-thirds of long-stay NH residents[11]—are likely to be particularly affected by sleep disturbance.[12,13]

Ample evidence underscores the negative health effects of sleep disturbance (lower total sleep time, increased sleep fragmentation, etc.) in older adults. Poor sleep quality in this population is associated with increases in self-reported fatigue, difficulties with activities of daily living (ADLs), depression, and risk of falling, and with decreases in memory, mobility, morbidity, and even survival.[3,41-44] Yet nursing home (NH) residents with Alzheimer's disease and related dementias (ADRD) experience suboptimal sleeping conditions at best. They spend much of their day without engaging in physical activity.[45,46] In addition, stimuli inherent in most traditional NH environments, including frequent noise and light [47-49] as well as, for example, efforts to reduce incontinence,[50] may also exacerbate sleep disturbance. A 2018 systematic review of nonpharmacological sleep interventions in NHs indicated promise for increased daytime light exposure, nighttime use of melatonin, and acupressure.[51] But because sleep itself results from a complex interplay of resident, staff-generated, and environmental factors,[52] sleep regimens for residents may work best when individualized instead of applied generically.[53,54] Yet healthcare workers, including those working in NHs, have generally poor knowledge of the relationship between sleep disturbance and clinical conditions, the availability of evidence-based interventions and assessment tools, and the environment's impact on sleep,[55] thus limiting their ability to intervene.

NH residents with ADRD are more likely to receive antipsychotic or antidepressant medications than other residents.[56] Yet antipsychotic or antidepressant use may worsen nighttime sleep for residents with ADRD,[57] while withdrawal from antipsychotic medication may also, at least temporarily, worsen sleep.[58] Pain may also affect sleep. The American Geriatrics Society and National Institute on Aging 2016 conference on sleep called for more research into the relationship between sleep disturbance and pain.[59] A critical review examining prospective studies from 2005 to 2012 found significant support for—in what is a bidirectional relationship between sleep and pain—sleep disturbance as the stronger predictor.[60] The relationship between sleep quality and physical functioning (e.g., ADLs) is well-established. Poorer objective sleep is associated with poorer physical functioning in older adults [3,6,44,61] and those with ADRD.[62]

Despite the common knowledge that sleep disturbance has negative impacts on NH residents, and despite the availability of easy ways to measure (e.g., actigraphy) and intervene to increase sleep, little has been done to improve the situation. In NHs, barriers to quality improvement (QI) such as staffing problems and top-down approaches hamper efforts to enhance care quality.[14,15] Studies in NHs also underscore the importance of open communication and relationship-building to improve resident clinical outcomes.[14,16,17] A recent systematic review highlighted key components for successful NH QI: changing staff behavior, targeting specific care tasks, and using intervention theories.[18] One QI practice that capitalizes on this evidence and therefore has potential to meaningfully impact resident sleep is frontline staff huddling—brief, stand-up meetings to facilitate efficient, collaborative information exchange. Frontline staff huddling promotes communication across clinical roles[19-21] and improvements in clinical outcomes.[22-24] In NHs, it can improve quality of care and help sustain changes.[25] But use of frontline staff huddling in NHs remains limited.

Our research team standardized a NH frontline staff huddling program building on

the theory of relational coordination, which posits that high-quality communication and relationships improve outcomes.[26] The program, known as LOCK (see Figure 1), is derived from evidence supporting strengths-based learning,[27,28] systematic observation[29] relationship-based teamwork,[30,31] and efficiency.[32,33] In LOCK, staff (A) “Learn from bright spots” (focus on evidence of positive change); (B) “Observe” (collect data through systematic observation); (C) “Collaborate in huddles” (conduct frontline huddles); and (D) “Keep it bite-size” (limit activities to 5-15 minutes for efficiency). The program’s methods have improved clinical care in community NHs, even in those with a history of serious, intransigent quality issues. [35,36] The program guides frontline staff on how to address a particular resident outcome of concern, in the case of this pilot study, sleep (LOCK sleep program).

The LOCK program was implemented to target NH staff-resident interactions in a successful
6-site pilot study in VA NHs. [34] Our LOCK program pilot targeting staff-resident interactions enabled 6 Veterans Health Administration (VA) NHs to make meaningful quantitative and qualitative improvements in communication frequency, communication quality, staff QI mindset, and staff QI capabilities.[34]

In 2017, based on these results, VA rolled the LOCK program out to all its 134 VA NHs. We achieved this roll-out with the support of B&F Consulting (national experts in NH QI implementation, and consultants for this current pilot study). The initial rollout was performed using a train-the-trainer approach, [37-39,81] with 4 participants from each NH attending a regional training led by B&F Consulting. The program then expanded to focus on other clinical issues, e.g., pain, catheter use, and pressure ulcers. Because all NHs in the VA system were exposed, a matching analysis of exposed vs. control NHs was not possible. Semi-structured qualitative interviews with staff at 12 VA NHs that excelled in the program revealed a wide range of improvements including the following: increases in resident engagement, staff morale, and staff engagement; reductions in falls and catheter-associated urinary tract infections; and increases in use of appropriate infection control, appropriate analgesic documentation, nonpharmacologic pain management, and pressure ulcer prevention.

We next conducted a preliminary adaptation of the LOCK program methods to address
resident sleep in a group of 8 VA NHs. Qualitative data based on field notes from two 1.5-day site visits to each of the 8 NHs provide preliminary evidence of the program’s acceptability, feasibility, and positive outcomes. Across all 8 NHs, we found consistent uptake of sleep intervention efforts, including resident sleep assessments (sleep behavior tracking/actigraph use/sunlight exposure tracking), individualized reduction of night-time incontinence care practices, alterations of medication administration schedules, reduction of night-time blue light and noise, and alterations of daytime activity schedules and natural light exposure. Outcomes include reduced pain, reduced psychotropic use, increased daytime activity, and resident and family enthusiasm for sleep program participation.

In sum, this preliminary work (1) enabled us to standardize the LOCK program, (2) resulted in qualitative data on best LOCK program implementation practices, and (3) generated implementation materials, all of which we will use for this current pilot study. During this pilot study, we will refine the LOCK program to focus on sleep in non-VA NHs.

[SELECTED REFERENCES]:
1. McCrae CS, McNamara JP, Rowe MA, et al. Sleep and affect in older adults:

using multilevel modeling to examine daily associations. Journal of sleep research. 2008;17(1):42-53.
2.Song Y, Blackwell T, Yaffe K, Ancoli-Israel S, Redline S, Stone KL. Relationships between sleep stages and changes in cognitive function in older men: the MrOS Sleep Study. Sleep. 2015;38(3):411-421.
3.Song Y, Dzierzewski JM, Fung CH, et al. Association Between Sleep and Physical Function in Older Veterans in an Adult Day Healthcare Program. J Am Geriatr Soc. 2015;63(8):1622-1627.
4.Suh SW, Han JW, Lee JR, et al. Sleep and cognitive decline: A prospective nondemented elderly cohort study. Annals of neurology. 2018;83(3):472-482.
5.da Silva AA, de Mello RG, Schaan CW, Fuchs FD, Redline S, Fuchs SC. Sleep duration and mortality in the elderly: a systematic review with meta-analysis. BMJ open. 2016;6(2):e008119.
6.Dam TT, Ewing S, Ancoli-Israel S, Ensrud K, Redline S, Stone K. Association between sleep and physical function in older men: the osteoporotic fractures in men sleep study. J Am Geriatr Soc. 2008;56(9):1665-1673.
7.Livingston G, Blizard B, Mann A. Does sleep disturbance predict depression in elderly people? A study in inner London. The British journal of general practice : the journal of the Royal College of General Practitioners. 1993;43(376):445-448.
8.Miner B, Kryger MH. Sleep in the Aging Population. Sleep medicine clinics. 2017;12(1):31-38.
9.St George RJ, Delbaere K, Williams P, Lord SR. Sleep quality and falls in older people living in self- and assisted-care villages. Gerontology. 2009;55(2):162-168.
10.Stenholm S, Head J, Kivimaki M, et al. Sleep Duration and Sleep Disturbances as Predictors of Healthy and Chronic Disease-Free Life Expectancy Between Ages 50 and 75: A Pooled Analysis of Three Cohorts. J Gerontol A Biol Sci Med Sci. 2019;74(2):204-210.
11.Harris-Kojetin L SM, Park-Lee E, Valverde R. Long-term care services in the United States: 2013 overview. National Center for Health Statistics. Vital Health Stat 3(37). 2013.
12.Kume Y, Kodama A, Sato K, Kurosawa S, Ishikawa T, Ishikawa S. Sleep/awake status throughout the night and circadian motor activity patterns in older nursing-home residents with or without dementia, and older community-dwelling people without dementia. Int Psychogeriatr. 2016;28(12):2001-2008.
13.Wennberg AMV, Wu MN, Rosenberg PB, Spira AP. Sleep Disturbance, Cognitive Decline, and Dementia: A Review. Semin Neurol. 2017;37(4):395-406.
14.Arling G, Cooke V, Lewis T, Perkins A, Grabowski DC, Abrahamson K. Minnesota's provider-initiated approach yields care quality gains at participating nursing homes. Health Aff (Millwood). 2013;32(9):1631-1638.
15.Collier E, Harrington C. Staffing characteristics, turnover rates, and quality of resident care in nursing facilities. Res Gerontol Nurs. 2008;1(3):157-170.
16.Anderson RA, Issel LM, McDaniel RR, Jr. Nursing homes as complex adaptive systems: relationship between management practice and resident outcomes. Nurs Res. 2003;52(1):12-21.
17.Forbes-Thompson S, Leiker T, Bleich MR. High-performing and low-performing nursing homes: a view from complexity science. Health Care Manage Rev. 2007;32(4):341-351.
18.Low LF, Fletcher J, Goodenough B, et al. A Systematic Review of Interventions to Change Staff Care Practices in Order to Improve Resident Outcomes in Nursing Homes. PLoS One. 2015;10(11):e0140711.
19.Melton L, Lengerich A, Collins M, et al. Evaluation of Huddles: A Multisite Study. Health Care Manag (Frederick). 2017;36(3):282-287.
20.Stapley E, Sharples E, Lachman P, Lakhanpaul M, Wolpert M, Deighton J. Factors to consider in the introduction of huddles on clinical wards: perceptions of staff on the SAFE programme. Int J Qual Health Care. 2018;30(1):44-49.
21.Walsh A, Moore A, Everson J, DeCaire K. Gathering, strategizing, motivating

and celebrating: the team huddle in a teaching general practice. Educ Prim Care. 2018;29(2):94-99.
22.Brass SD, Olney G, Glimp R, Lemaire A, Kingston M. Using the Patient Safety Huddle as a Tool for High Reliability. Jt Comm J Qual Patient Saf. 2018;44(4):219-226.
23.Newman RE, Bingler MA, Bauer PN, Lee BR, Mann KJ. Rates of ICU Transfers After a Scheduled Night-Shift Interprofessional Huddle. Hosp Pediatr. 2016;6(4):234-242.
24.Tielbur BR, Rice Cella DE, Currie A, et al. Discharge huddle outfitted with mobile technology improves efficiency of transitioning stroke patients into follow-up care. Am J Med Qual. 2015;30(1):36-44.
25.Brady C, Farrell D, Frank B, Elliot AE, Gittell JH. A long-term care leader's guide to high performance : doing better together. Baltimore: Health Professions Press; 2018.
26.Gittell JH, Godfrey M, Thistlethwaite J. Interprofessional collaborative practice and relational coordination: improving healthcare through relationships. J Interprof Care. 2013;27(3):210-213.
27.Dewar B, MacBride T. Developing Caring Conversations in care homes: an appreciative inquiry. Health Soc Care Community. 2017;25(4):1375-1386.
28.Vogt K, Johnson F, Fraser V, et al. An Innovative, Strengths-Based, Peer Mentoring Approach to Professional Development for Registered Dietitians. Can J Diet Pract Res. 2015;76(4):185-189.
29.Yanes AF, McElroy LM, Abecassis ZA, Holl J, Woods D, Ladner DP. Observation for assessment of clinician performance: a narrative review. BMJ Qual Saf. 2016;25(1):46-55.
30.Dewar B, Barrie K, Sharp C, Meyer J. Implementation of a Complex Intervention to Support Leadership Development in Nursing Homes: A Multimethod Participatory Study. J Appl Gerontol. 2017:733464817705957.
31.Sakai M, Naruse T, Nagata S. Relational coordination among home healthcare professions and goal attainment in nursing care. Jpn J Nurs Sci. 2016;13(3):402-410.
32.Mills WL, Pimentel CB, Palmer JA, et al. Applying a Theory-Driven Framework to Guide Quality Improvement Efforts in Nursing Homes: The LOCK Model. Gerontologist. 2018;58(3):598-605.
33.Phillips J, Hebish LJ, Mann S, Ching JM, Blackmore CC. Engaging Frontline Leaders and Staff in Real-Time Improvement. Jt Comm J Qual Patient Saf. 2016;42(4):170-183.
34.Hartmann CW, Mills WL, Pimentel CB, et al. Impact of Intervention to Improve Nursing Home Resident-Staff Interactions and Engagement. Gerontologist. 2018;58(4):e291-e301.
35.Farrell D, Brady C, Frank B. Meeting the leadership challenge in long-term care : what you do matters. Baltimore: Health Professions Press; 2011.
36.Smith DB. Assessment of the Critical Access Nursing Home Pilot: Final Report. Vineyeard Haven MA: Advancing Excellence and Drexel University School of Public Health;2012.
37.Franzmann J, Haberstroh J, Pantel J. Train the trainer in dementia care. A program to foster communication skills in nursing home staff caring for dementia patients. Z Gerontol Geriatr. 2016;49(3):209-215.
38.Mayrhofer A, Goodman C, Smeeton N, Handley M, Amador S, Davies S. The feasibility of a train-the-trainer approach to end of life care training in care homes: an evaluation. BMC Palliat Care. 2016;15:11.
39.Sampson EL, Vickerstaff V, Lietz S, Orrell M. Improving the care of people with dementia in general hospitals: evaluation of a whole-system train-the-trainer model. Int Psychogeriatr. 2017;29(4):605-614.
41.Garcia-Alberca JM, Lara JP, Cruz B, Garrido V, Gris E, Barbancho MA. Sleep disturbances in Alzheimer's disease are associated with neuropsychiatric

symptoms and antidementia treatment. J Nerv Ment Dis. 2013;201(3):251-257.
42.Koschwanez HE, Kerse N, Darragh M, Jarrett P, Booth RJ, Broadbent E. Expressive writing and wound healing in older adults: a randomized controlled trial. Psychosom Med. 2013;75(6):581-590.
43.Kurdziel LBF, Mantua J, Spencer RMC. Novel word learning in older adults: A role for sleep? Brain Lang. 2017;167:106-113.
44.Valenza MC, Cabrera-Martos I, Martin-Martin L, Perez-Garzon VM, Velarde C, Valenza-Demet G. Nursing homes: impact of sleep disturbances on functionality. Arch Gerontol Geriatr. 2013;56(3):432-436.
45.Anderiesen H, Scherder EJ, Goossens RH, Sonneveld MH. A systematic review--physical activity in dementia: the influence of the nursing home environment. Appl Ergon. 2014;45(6):1678-1686.
46.Harper Ice G. Daily life in a nursing home: Has it changed in 25 years? Journal of Aging Studies. 2002;16(4):345-359.
47.Schnelle JF, Alessi CA, Al-Samarrai NR, Fricker RD, Jr., Ouslander JG. The nursing home at night: effects of an intervention on noise, light, and sleep. J Am Geriatr Soc. 1999;47(4):430-438.
48.Schnelle JF, Cruise PA, Alessi CA, Ludlow K, al-Samarrai NR, Ouslander JG. Sleep hygiene in physically dependent nursing home residents: behavioral and environmental intervention implications. Sleep. 1998;21(5):515-523.
49.Schnelle JF, Ouslander JG, Simmons SF, Alessi CA, Gravel MD. The nighttime environment, incontinence care, and sleep disruption in nursing homes. J Am Geriatr Soc. 1993;41(9):910-914.
50.Ouslander JG, Ai-Samarrai N, Schnelle JF. Prompted voiding for nighttime incontinence in nursing homes: is it effective? J Am Geriatr Soc. 2001;49(6):706-709.
51.Capezuti E, Sagha Zadeh R, Pain K, Basara A, Jiang NZ, Krieger AC. A systematic review of non-pharmacological interventions to improve nighttime sleep among residents of long-term care settings. BMC Geriatr. 2018;18(1):143.
52.Pillai JA, Leverenz JB. Sleep and Neurodegeneration: A Critical Appraisal. Chest. 2017;151(6):1375-1386.
53.Ellmers T, Arber S, Luff R, Eyers I, Young E. Factors affecting residents' sleep in care homes. Nurs Older People. 2013;25(8):29-32.
54.Harris M, Grando V. When is nighttime? A description of bedtime in persons with dementia in the nursing home. Geriatr Nurs. 2014;35(6):474-478.
55.Brown CA, Wielandt P, Wilson D, Jones A, Crick K. Healthcare providers' knowledge of disordered sleep, sleep assessment tools, and nonpharmacological sleep interventions for persons living with dementia: a national survey. Sleep Disord. 2014;2014:286274.
56.Brimelow RE, Wollin JA, Byrne GJ, Dissanayaka NN. Prescribing of psychotropic drugs and indicators for use in residential aged care and residents with dementia. Int Psychogeriatr. 2018:1-11.
57.Simpson KM, Richards KC, Enderlin CA, O'Sullivan PS, Koehn M. Medications and Sleep in Nursing Home Residents With Dementia. Journal of the American Psychiatric Nurses Association. 2006;12(5):279-285.
58.Ruths S, Straand J, Nygaard HA, Bjorvatn B, Pallesen S. Effect of antipsychotic withdrawal on behavior and sleep/wake activity in nursing home residents with dementia: a randomized, placebo-controlled, double-blinded study. The Bergen District Nursing Home Study. J Am Geriatr Soc. 2004;52(10):1737-1743.
59.Fung CH, Vitiello MV, Alessi CA, Kuchel GA. Report and Research Agenda of the American Geriatrics Society and National Institute on Aging Bedside-to-Bench Conference on Sleep, Circadian Rhythms, and Aging: New Avenues for Improving Brain Health, Physical Health, and Functioning. J Am Geriatr Soc. 2016;64(12):e238-e247.
60.Finan PH, Goodin BR, Smith MT. The association of sleep and pain: an update and a path forward. The journal of pain : official journal of the American Pain

Society. 2013;14(12):1539-1552.
61.Parmelee PA, Tighe CA, Dautovich ND. Sleep disturbance in osteoarthritis: linkages with pain, disability, and depressive symptoms. Arthritis care & research. 2015;67(3):358-365.
62.Tractenberg RE, Singer CM, Kaye JA. Symptoms of sleep disturbance in persons with Alzheimer's disease and normal elderly. Journal of sleep research. 2005;14(2):177-185.
81.Colon-Emeric C, Toles M, Cary MP, Jr., et al. Sustaining complex interventions in long-term care: a qualitative study of direct care staff and managers. Implementation science : IS. 2016;11:94.

b) Describe any animal experimentation and findings leading to the formulation of the study, if there is no supporting human data.

N/A

2) Purpose of the study

a) Provide a brief lay summary of the project in <200 words. The lay summary should be readily understandable to the general public.

This study will improve clinical outcomes for an important, growing, and vulnerable population—nursing home (NH) residents with Alzheimer's disease or related dementias (ADRDs)—by implementing an evidence-based intervention (LOCK) to improve these residents' sleep. It will also significantly increase our understanding of how to implement and sustain nursing home interventions.

b) List your research objectives (specific aims & hypotheses of the study).

In community (non-VA) NHs [one from each of 3 NH corporations, still to be recruited], our multi-disciplinary team proposes to (1) refine the LOCK sleep program to focus on sleep for residents with ADRD during this pilot (R61) phase, (2) which will prepare the team for the next phase of this NIH-funded grant (the R33 phase, to be described in a follow-up IRB protocol when this phase is completed, in approximately one year) to test the impact and sustainability of this intervention for NH residents with ADRD in an incomplete stepped-wedge randomized controlled trial.

(This study is funded by a National Institute of Aging pragmatic clinical trial RFA and grant mechanism called R61/R33, in which the R61 is a pilot grant for one year. The investigator team submits a report after the R61 phase indicating what was learned during the pilot phase and showing evidence that the pilot milestones are achieved. Then NIA funds the next four years of the randomized controlled trial with the R33 mechanism. The current proposal only describes the pilot study (R61 phase). A separate proposal will be submitted for the randomized controlled trial (R33) when the R61 phase is close to completion. We will wait to submit the R33 proposal because National Institute of Aging wishes for modifications to the R33 design and intervention to be made based on the R61 data collection/intervention experience and the R61 quantitative and qualitative data analyses.)

THIS CURRENT PILOT STUDY R61 PHASE (1 YEAR; N = 3 NHS; 1 NH PER CORPORATION) HAS THE FOLLOWING SPECIFIC AIMS:
1. Refine the LOCK sleep program train-the-trainer protocol by implementing and pilot-testing in three NHs.
2. Test and refine the research methods to: effectively identify eligible NHs and residents; obtain consent; collect primary data from residents and staff; explore staff impressions of additional sleep measurement devices (Fitbits); transfer primary and secondary data to our data center; and merge all data.

c) Describe the study design (e.g., single/double blind, parallel, crossover, control, experimental, observational, etc.)

N/A
Single blind
Double blind
Parallel
Crossover
Control group
Experimental group
Observational
X Other

This is a pilot study (R61 phase) to pilot the methods for an upcoming randomized controlled trial (see study funding mechanism explanation in previous section #2B above). The upcoming randomized controlled trial will be an incomplete stepped-wedge, cluster randomized controlled trial (RCT) design in which each nursing home (NH) serves as its own control. Our design can be classified in the NIH Model for Behavioral Intervention Development as Stage III, real world-based hybrid efficacy-effectiveness research.

This pilot study (R61 phase) pilots the methods for this design in 3 NHs. In this pilot we will use a convenience sample of three nursing homes (NHs), one recruited from each of three different NH corporations that are anticipated to participate in the eventual randomized controlled trial.

Each nursing home will serve as its own control. Control data will be collected for 8 weeks, then the intervention will begin and intervention data will be collected for 6 weeks, then the sustainment phase will begin and sustainment data will be collected for 8 weeks.

d) **Provide a timeline for individual participant recruitment and follow-up (analysis for the study is required).**

Each site will serve as its own control.

Control data will be collected for 8 weeks, then the intervention will begin and intervention data will be collected for 6 weeks, then the sustainment phase will begin and sustainment data will be collected for 8 weeks.

The duration of the intervention period for any NH is 22 weeks, which includes four 7-day sleep measurement periods (pre-baseline measurement before week 1, baseline measurement at week 8, post-intervention measurement at week 15, and sustainment measurement at week 22).

e) **Will participant be randomized?** N

f) **If participants will be given placebo, please justify placebo use, and describe contents of the placebo.**

N/A

3) Study Procedures

a) **Is this project a multicenter study (i.e., same project is conducted elsewhere by a different investigator)?** N

Is University of Alabama acting as a coordinating center for other sites?

Will the University of Alabama site be participating in all parts/procedures/arms of the study?

If No, explain what University of Alabama will NOT participate in:

b) **Describe all the procedures, from screening through end-of-study, that the human participant must**

undergo in the research project, including study visits, drug treatments, randomization and the procedures that are part of standard of care. Specify which procedures are for research and which are standard of care. If study involves only retrospective record review, describe that review process here, including how records will be selected for review. Please note: The box below is for text only. If you would like to add tables, charts, etc., Click "Add" to attach the documents.

RESEARCH STUDY TEAM: The study leadership team will be led by Dr. Snow (UA; LOCK sleep program co-developer, clinical geropsychologist, NH field expert) and will include Dr. Parmelee (UA; NH Minimum Data Set and sleep expert), Dr. Richards (Univ. TX School of Nursing; nurse, clinical NH and dementia sleep expert), Dr. Fry (Univ. TX Medical School, geriatrician, clinical geriatrics NH expert); Dr. Morgan (Univ TX School of Public Health, study statistician, data center lead), Dr. Hartmann (UMass Lowell; LOCK sleep program co-developer; social worker; implementation science expert), Dr. MCullough (UMass Lowell, medical anthropologist, qualitative expert), Ms. Baier
and Dr. McCreedy (Brown University's Long-Term Care Quality and Innovation Center; NH implementation experts, NH recruitment experts), and B&F Consulting (national experts in NH quality improvement implementation). The project team, comprising Drs. Snow and Hartmann and (To Be Named in Future Amendment) research staff, will meet weekly. The following teams will each meet bi-weekly: NH staff training team (Snow, B&F Consulting); Quantitative/Clinical Data Team (Hartmann, Richards, Morgan, Fry, Parmelee, research staff). This following team will meet monthly: Qualitative/Implementation Team (Hartmann, McCullough, Baier, McCreedy). The entire investigator team will meet monthly.

COLLABORATING SITES AND ROLES:
The research will be performed by the research study team located at the University of Alabama, University of Massachusetts-Lowell, Brown University, University of Texas at Austin (School of Nursing and Dell Medical School), and the University of Texas at Houston (School of Public Health). Only the University of Alabama site will enroll human subjects and the University of Alabama IRB will serve as the single IRB. Dr. Snow at the University of Alabama will be responsible for oversight of the entire project. Collaborating sites where human subjects research will be performed are the 3 NHs who will be participating (to be named in future amendment). These nursing homes will be drawn from three separate NH corporations.

NH RECRUITMENT:
We are in the process of recruiting NH corporations for this study and will submit an amendment to this protocol when we have identified the participating facilities.

We will work closely with the 3 recruited corporations to identify 3 NHs (1 per corporation) to participate in this pilot study. Stakeholder engagement will occur at both the corporate and the NH levels. At the corporate level, each corporation will assign a corporate coach who will attend all study staff training visits to their corporation's NH to learn how to prepare NHs for and support NHs in the implementation of the LOCK sleep program. At the NH level, each NH will create LOCK sleep program NH Leadership and NH Implementation teams and will support frontline staff involvement in the LOCK sleep program.

The study team will assist each of these three corporations and their participating NHs in applying for a Federalwide Assurance (FWA) to designate the University of Alabama IRB as the IRB authorized to review, approve, and oversee the proposed study at each NH.

•NH Leadership Team: Each NH will establish a NH leadership team consisting at least of the Director of Nursing, the NH administrator, the staff educator, and the QI designee (additional members may be added if needed to assist with limited study

support activities as described below). The NH leadership team will be trained by the study team in protection of human subjects and limited applicable study procedures, i.e., (1) mailing introductory letters to resident LARs; (2) mailing of actigraphs/Fitbits and global rating forms to research staff; and (3) helping researchers arrange telephone calls with NH residents who may have capacity to provide their own consent.

•NH Leadership Teams WILL ENGAGE in LIMITED STUDY SUPPORT ACTIVITIES, as follows:
(1) Mailing an IRB-approved study introduction letter to the legally authorized representative (LAR) of each NH resident with an ADRD diagnosis and providing all NH staff with a study introduction letter.
(2) Collecting and mailing outcome data (e.g., actigraphs at completion of a 7-day measurement period, completed staff ratings of resident sleep global change) to the University of Alabama project office using UA-supplied self-addressed postage-paid mailers from a tracked express mailing service (e.g., UPS).
(3) Assisting in telephone appointment arrangements and logistical support so that study team members can conduct telephone-based informed consent for NH residents who may have capacity to provide their own consent.

•NH leadership teams WILL NOT ENGAGE in RESEARCH STUDY TASKS which will be completed exclusively by research study team members with appropriate study protection training:
(1) Conducting informed consent with staff, LARs, and residents.
(2) Conducting staff interviews: staff decisions whether or not to participate in interviews will be kept confidential from NH leadership teams to protect staff from potential coercion to participate in research.

SUBJECT POPULATIONS AND ANTICIPATED NUMBERS:
(1) NH RESIDENTS. Primary outcomes are measured at the level of NH residents. We anticipate enrolling 57 residents (approximately 19 per each of 3 NHs) in this pilot study (R61 phase).
(2) NH STAFF.
a. Training and Intervention Participation.
We will invite all NH staff to participate in the training and intervention. In the 3 NHs in this pilot study (R61 phase) we anticipate enrolling 120 NAs, 45 LPNs, 5 RNs, and 30 interdisciplinary treatment team/leadership team members. There are no known risks to the nursing staff associated with the training, intervention, and resident assessment activities requested of the NH staff. These are resident care and quality improvement activities that are fully within their job descriptions. Consequently, consent for participation from the NH staff in these activities is not considered necessary as these activities are not human subjects research. In our past funded research involving NH staff, consent for these types of activities has also not been considered necessary. The nursing staff should benefit greatly from learning frontline huddling and sleep intervention skills. If successful, their application of these interventions will result in stronger NH staff teamwork and communication and healthier and less distressed NH residents and thus contribute to a less stressful and dangerous work environment.
b. Interview Participation.
We anticipate inviting 20 NH staff per NH (10 frontline staff engaged in the intervention, 10 LOCK sleep program leadership and intervention team members) to participate in interviews. In the 3 NHs in this pilot study (R61 phase) we anticipate enrolling 60 staff total. Because participation in qualitative interviews is outside of the usual work activities of the NH staff, we will obtain informed consent from NH staff before asking them to voluntarily participate in these activities. The NH staff will be informed of the purpose of all procedures: there is no element of

deception. They will not be identified personally in any data collection used for research purposes. There will be no adverse consequences for NH staff refusing to take part in these activities.

NH RESIDENT INCLUSION AND EXCLUSION CRITERIA: In the potential participant pool, we will include all NH residents aged >=50 years with an ADRD diagnosis. We include residents across the range of ADRD severity because this is consistent with the LOCK sleep program, in which staff will focus on residents with ADRD who have the greatest sleep problems without differentiating by ADRD severity. To identify participants, NH staff will use frontline staff huddles. NH staff will be trained to use the STOP-Bang screening tool to identify NH residents with high risk of obstructive sleep apnea (OSA) [82-84] and will be trained on appropriate procedures for referring any positively screened residents for medical evaluation. We will exclude residents with a high risk of OSA who are not being treated for OSA because actigraph measurements are inaccurate in that population.[85] Staff will also exclude residents who have a persistent bilateral resting tremor or paralysis in both arms (a subset of persons with Parkinson's disease and related significant tremor-causing diagnoses), due to actigraph measurement inaccuracies.[86]

NH STAFF INCLUSION AND EXCLUSION CRITERIA:
Staff will not be excluded on the basis of race, ethnicity, gender, or age. As described above, a subset of NH staff will be recruited for interview participation based upon job type and intervention engagement (e.g., frontline staff versus leadership/interdisciplinary team members).

OBTAINING NH RESIDENT AND STAFF CONSENT:
NH RESIDENT CONSENT: We will ask the administration department at each NH to compile a list of all NH residents with an ADRD diagnosis. The administration department will send a letter to each resident's legally authorized representative (LAR) informing them of the study and inviting the LAR to opt out if they do not wish to be contacted by research staff. Opt-out procedures have been demonstrated to yield higher response rates and lower rates of non-response bias compared to opt-in procedures, and to be acceptable to participants, with no difference in rates of reported distress or complaints compared to opt-in procedures.[105-108] In our past federally funded research involving people with dementia, opt-out
consent has been approved by multiple IRBs and worked well. Contact information of those LARs not
opting out will be shared with University of Alabama (UA) study staff. We are requesting of this IRB a waiver of consent and HIPAA authorization for screening purposes regarding the sharing of these contact information. Research staff will contact all LARs who do not opt out, inviting the LAR to consent to the NH resident's participation. The consent process will be described in more detail in the consent portion of this IRB proposal.

NH STAFF CONSENT: The NH leadership team or their designees will provide to all NH staff an IRB-approved study introduction letter (including option to opt out within two weeks if they do not wish to be contacted by study staff). Contact information of those NH staff not opting out will be shared with UA study staff. We are requesting of this IRB a waiver of consent and HIPAA authorization for screening purposes regarding the sharing of these contact information.
Research study staff will contact NH staff (via email and/or phone) to invite them to participate in mid-implementation and/or post-implementation interviews about their experiences with the LOCK sleep program and Fitbits. The list of NH staff who agree and decline interview participation will remain confidential to protect NH staff from any possible coercion to participate. The consent process will be described in

more detail in the consent portion of this IRB proposal.
NOTE: The LOCK sleep program itself comprises clinical practices common in NH QI efforts. These clinical practices that will be taught via the LOCK sleep program training thus fall within the scope of staff positions and require no consent.

SOURCES OF RESEARCH DATA (see Table 1):
NH RESIDENT DATA will be collected for consented NH residents from NH staff ratings (clinical global assessments of change in overall sleep quality and sleep-related conditions - see Appendix); through primary data collection (actigraph/Fitbit measurements; these are wristwatch-sized devices worn on the wrist); through medical records (age, diagnoses, medical history, and medications); and through NH secondary data from the Minimum Data Set (MDS; all Medicare-reimbursed NHs are required to collect MDS assessments on all residents; the MDS contains information on resident medical conditions, functioning, cognition, psychotropic and pain/analgesic medication use, activities of daily living decline, and sleep-, mood-, and activity-related items). Table 1 summarizes the primary and secondary outcomes and data sources.

NH STAFF DATA staff interview data will be collected via audiorecorded qualitative interview (see Appendix).

DATA COLLECTION PROCEDURES:
NH RESIDENT PRIMARY RESEARCH DATA COLLECTION will consist only of actigraph/fitbit data.

We will measure total sleep time (TST) with data from Micro-Mini Motionlogger Actigraphs (Ambulatory Monitoring Inc., Ardsley, NY).[87] We will define nighttime as 10pm to 6am and will compute TST as the total number of minutes asleep during a nighttime period. We will measure sleep for 7 days at each measurement period (see Figure 2) to obtain reliable measurements and will use the average TST across this period. We will also examine the following: wake after sleep onset (total number of minutes awake during nighttime), how often the resident awoke during the nighttime, sleep efficiency (the ratio of minutes asleep to minutes awake over the period), and sleep fragmentation (an index of restlessness computed as the percentage of one-minute epochs scored as awake). Sensitivity of actigraph measurement for sleep is very good (actigraphy = sleep when polysomnography = sleep was 0.97). [88]

We will also explore measuring sleep time using Fitbits. Fitbits are known to be inferior to actigraphs for sleep research measurement purposes because Fitbits tend to overestimate total sleep time, among other limitations.[70,71] But Fitbits are considerably less expensive than actigraphs, offer an easy user interface, and collect data on sleep plus multiple other dimensions, making them potentially useful to NH staff for non-research purposes. Studies are lacking on NH staff impressions of the perceived potential usefulness of Fitbits for informal measurement. We will therefore explore staff impressions of Fitbits through semi-structured interviews after the R61's 22-week measurement period. If staff find Fitbit information of added use and staff and residents consistently find it acceptable to use 2 devices at the same time (actigraph plus Fitbit), we will consider use Fitbits beyond this pilot study into the randomized controlled trial (R33 phase).

Dr. Kathy Richards, PhD MSN (nurse co-investigator with extensive NIA and VA principal investigator experience using objective sleep measurements including actigraphs in studies with NH residents and people with dementia, who tolerated the devices well without removing them.[109-111] NH staff will assist residents to

wear both an actigraph and a Fitbit side by side on a wrist for 7 days during each of the 4 measurement periods (weeks 1, 8, 15, and 22—see Figure 2). We will use Dr. Richards' effective procedures for assuring that NH staff maintain charge and do not lose actigraphs or Fitbits (e.g., Nighttime Agitation and Restless Legs Syndrome in People with Alzheimer's disease, R01 AG051588-01A1, NIH-funded).

NH STAFF DATA ABOUT NH RESIDENTS: The NH Leadership and NH Implementation teams will be trained by our research study team on the LOCK Sleep Program methods (see Figure 1), including how to facilitate and implement front-line staff huddles to discuss resident sleep issues. Once this training is complete, we will ask NH huddle facilitators to use a huddle to establish a team consensus rating of each enrolled NH resident's overall sleep quality and up to 2 additional symptoms or behaviors of concern potentially related to sleep quality (e.g., agitation, pain). Ratings will be obtained using the using the Clinical Global Impression of Change rating scale (CGIC; see Appendix); CGIC ratings evidence high reliability and responsivity under a wide range of circumstances and are frequently used as outcomes in clinical trials. [97-104] Each LOCK sleep program huddle facilitator will lead their huddle team to complete clinical global impression of change ratings of enrolled residents' overall sleep quality at the end of each 7-night sleep measurement period and at the end of each week of the 6-week sleep intervention period. These rating forms will be part of the standard care practices being taught to the NH staff -- such assessments are within the typical scope of staff responsibilities. The NH clinical team will file these assessments in the NH residents' medical chart. The NH Leadership team will copy these rating forms and share with the research team.

NH STAFF INTERVIEWS: We will recruit and consent a sample of NH staff engaged in huddles to participate in mid- (phone) and post-implementation (in-person or phone) semi-structured interviews to explore staff perceptions of the LOCK sleep intervention's effectiveness, feasibility, facilitators, and challenges, as well as their impressions of the interview process itself to inform for the upcoming randomized controlled trial (R33 phase) (e.g., length, location, etc. for busy NH staff) and use of Actigraphs and Fitbits (see Appendices for interview guides). For the post-implementation interviews, we will send a research staff member trained in qualitative interviewing to each site to conduct these interviews. For both interviews, we will recruit the LOCK sleep program leadership and implementation team members, as well as a sample of frontline staff, with an approximate sample size of 20 staff per NH.

MEDICAL RECORD/SECONDARY DATA ABOUT NH RESIDENTS: To assess inter-resident variability in sleep, a research assistant will visit the NH at the end of the 22-week measurement period to collect data from the NH's medical record on the following for the entire period: (a) changes in any sedating medications and changes in dosages; (2) incidents of delirium; (3) any urinary tract infections; (4) doses of any sedating medications, including as needed ones. Also collected from medical records will be information to characterize the NH residents (age, diagnoses, medical history, and medications). NH secondary data will be collected also from the NH Minimum Data Set (MDS; all Medicare-reimbursed NHs are required to collect MDS assessments on all residents; the MDS contains information on resident medical conditions, functioning, cognition, psychotropic and pain/analgesic medication use, activities of daily living decline, and sleep-, mood-, and activity-related items).

EXTRACTING, TRANSFERRING, AND MERGING DATA: Every week, the NH Leadership team will use a researcher-provided tracked insured express mail service (e.g., UPS) to send the research team at the University of Alabama all

completed Clinical Global Impressions of Change staff rating forms and any actigraphs and Fitbits for which the assessment periods are complete. Research staff will mail back actigraphs and Fitbits after download and maintenance. We considered electronic data transmission options but opted for mail for the following reasons: (a) We will be able to assure equipment maintenance and appropriate data downloads, reducing missing data errors. (b) For busy NH staff, copying and packaging will take significantly less time and technological skill than scanning, uploading, and transmitting electronic data. Thus, after research staff upload the data at the University of Alabama, they will transfer the data to the University of Texas Health Science Center at Houston School of Public Health (UTHealth SPH) Data Center via secure file transfer procedures established by the Data Center. The data transfer and data security processes will be discussed in more detail in Section #10 (Procedures To Maintain Confidentiality).

STUDY STAFF TRAINING
This study does involve the vulnerable population of nursing home residents with dementia. This study also involves NH employees, who are vulnerable to the extent that they may feel coerced to participate in research activities by their supervisors given that the NH leaders and parent corporation are supportive of overall study participation. To protect all vulnerable subjects, in addition to the NIH-required computer-based trainings (on the protection of human research participants, HIPAA, and Good Clinical Practice), the UA study staff will attend an in-person 2-day training session specific to the proposed project led by Dr. Snow. The PI will review the overall goals of the study, study policies and procedures, data collection manuals, adverse event identification and reporting, subject confidentiality, communication techniques and principles for working with persons with dementia, and appropriate procedures for working by telephone with LARs for consent, for working by telephone with people with dementia for consent and for determining capacity to consent. Procedures will be reviewed for protecting employees from possible coercion for participation from supervisors including maintaining confidentiality of those employees who do and do not consent to research interviews. During these training sessions, professionally produced videotapes of staff interactions with persons with dementia will be shown and the PI and study staff will role play all informed consent, capacity assessment, and data collection processes. Drs. McCullough and Hartmann (via skype) will provide training on appropriate procedures for NH staff interviews. Dr. Morgan (via skype) will provide training on appropriate procedures for data safety and security.

Dr. Richards (via skype) and Parmelee (in person) will work together to provide training on appropriate assessment equipment (e.g., actigraph, fitbit) maintenance and data downloading. For example, staff will be instructed that all portions of the bands and backs of the actigraph/fitbits should be carefully and thoroughly disinfected with a Sani-Cloth prime germicidal disposable wipe (or similar product that is appropriate for healthcare equipment that comes into patient contact such as stethoscopes) and left on the device for 1 minute as per product instructions to reach maximum bactericidal/fungicidal/virucidal/tubercolocidal effectiveness. The watch front of the actigraph/fitbits should be disinfected with a Sani-Cloth Easy Screen Cleaning Wipe (or similar product that is a 70% isopropyl alcohol solution and is designed for use on touch screens and other electronic devices that are degraded by more intensive chemicals).

NH LEADERSHIP TEAM TRAINING:
Study staff will provide a 2-hour training on procedures to protect vulnerable subjects, as follows: proper procedure for, and importance of only using IRB-approved procedures and letters when sending study opt-out letters to LARs; proper procedures for maintaining security and privacy of data packages mailed to

the University of Alabama project office; proper procedures for avoiding coercion or appearance of coercion of research participation when working with NH staff (e.g., not asking staff about research interview participation); proper procedures for assisting in telephone appointment arrangements and providing logistical support when study team members conduct telephone-based NH resident consenting and capacity to consent assessment; importance of only study team members conducting actual consent procedures of anyone (LARs, NH residents, NH staff); importance of only properly trained NH leadership team members participating in study procedures.

Dr. Richards and Parmelee (via skype teleconference) will work together to provide training on the appropriate application of actigraphs and Fitbits to promote comfort and prevent adverse experiences, as well as appropriate assessment equipment maintenance and data mailing. For example, the NH leadership team will be trained on the appropriate actigraph/fitbit cleaning as described in the previous subsection (STAFF TRAINING). They will also be taught that actigraphs/fitbits should only be applied to dry, clean skin. The skin should be gently washed with the skin cleanser approved for facility use for the nursing home resident (typically a non-soap cleanser such as cetaphil, but this may differ depending upon the specific resident), gently rinsed with a clean damp washcloth, and then carefully dried to avoid trapping moisture between the actigraph/fitbit and skin. Similarly, when the actigraph/fitbit is removed in the morning, the skin should be again cleansed and dried.

NH STAFF INTERVENTION TRAINING AND INTERVENTION IMPLEMENTATION

NH Staff Training will follow train-the-trainer principles, an established, effective mechanism for training NH staff.[37-39,81] To prepare for the training, B&F Consulting (Barbara Frank & Cathie Brady, research study team members, national NH quality improvement implementation experts, authors of two books on their method which highly influenced the development of the LOCK Sleep Program [25,35]) will guide each NH in the establishment of LOCK sleep program teams: (a) a NH Leadership team consisting of the Director of Nursing, the NH administrator, the staff educator, and the QI designee and (b) an implementation team consisting, at minimum, of the Minimum Data Set (MDS) coordinator, unit managers, and the medical director. B&F Consulting will visit each NH 5 times over the course of the R61 phase to train and support the NHs; Dr. Snow and other research staff will also attend several visits and will attend all phone calls with the sites. The corporate coaches will attend all B&F consulting visits to their corporation’s NH to prepare for the subsequent randomized controlled trial (the R33 phase), when the corporate coaches will assume leadership of the training with B&F support. B&F Consulting’s will conduct visits to train leadership and staff and begin the implementation, in and then conduct visits to support implementation, and then then finally a visit to support sustainment.

The 3 NHs will implement the program starting at approximately the same time. Based on prior
experience (both in VA and in B&F’s experience in non-VA NHs), we anticipate an initial 8-week period in which each NH builds its frontline staff huddle practice across all units (Figure 2: pre-implementation). After this, each NH will begin the LOCK sleep intervention on all its units (Figure 2: implementation). An 8-week sustainment period follows (Figure 2). The leadership team will communicate broadly about the program, ensuring all staff (including new hires) are introduced to and maintain implementation of the LOCK sleep program and perform the periodic sleep measurements.

The training will include the following components. B&F Consulting will train the leadership and
implementation teams to guide staff to use huddles to identify residents with ADRD whose sleep is
disrupted, explore residents' personal histories with help from family, and develop action plans to
pilot test individualized, person-centered sleep improvement approaches. B&F Consulting will also
guide the teams to teach staff to use observation and data collection to monitor the impact of
their action plans and change the plans as appropriate. The training will also instruct staff in
actigraph/fitbit use. It will include information on why good sleep is important for residents with ADRD,
the etiology of poor sleep, evidence-based sleep improvement interventions (e.g., good sleep hygiene, minimizing noise and light and resident disturbance at night, maximizing engaging activity during the day, good nutrition and hydration practices to promote sleep). As an example of training in techniques to reduce night-time interruption, nursing staff will be trained on procedures for checking on the resident without disturbing them if asleep, such as the use of small flashlights pointed toward the floor using use amber illumination (rather than turning on overhead sleep disruptive blue lights) to allow the nursing staff to check the resident and room without awakening a sleeping resident. This increases safety because sleeping residents are residents who are not getting up. Whereas waking a resident to use the restroom can result in the resident not being able to easily go back to sleep, which then puts them at risk of getting up due to restlessness or agitation after the nurse leaves.

The training will emphasize the importance of customized individualized care, incremental approaches to treatment changes, and how to use huddles to integrate such interventions into individualized treatment plans. For example, "decrease in nighttime interruption" does not mean never checking on the resident during the night. Staff will be trained on the importance of using small PDSA (plan-do-study-act) cycles to assure that all care changes are pursued incrementally in an individualized, customized manner. This is not about one-size-fits-all care. There is no blanket edict to not interrupt the patient for the entire night. Rather, the instruction is for the team to consider and explore ways of promoting longer periods of uninterrupted sleep, and to test these innovations in incremental trials over a period of days or weeks.

NH STAFF TRAINED TRAINERS PROVIDING TRAINING:
After B&F Consulting has trained the NH Leadership and implementation teams, those teams will then be responsible for training others in the NH. As part of the intervention training, NH staff will be trained by their NH Leadership or implementation teams or their trained designees on proper use and placement and cleaning of actigraphs and Fitbits, verbal and behavioral signs of distress that might indicate actigraph/Fitbit-related discomfort/distress, and appropriate modification techniques to try to relieve such distress. NH staff will be informed that the devices should be removed in the unlikely event that distress behaviors are not relieved by modification techniques. NH staff will be trained on importance of NH resident autonomy, and will be engaged in a discussion of how to assess for and honor NH resident assent or lack thereof with regard to the devices.

[SELECTED REFERENCES]
25.Brady C, Farrell D, Frank B, Elliot AE, Gittell JH. A long-term care leader's guide to high performance : doing better together. Baltimore: Health Professions Press;

2018.
35.Farrell D, Brady C, Frank B. Meeting the leadership challenge in long-term care : what you do matters. Baltimore: Health Professions Press; 2011.
37.Franzmann J, Haberstroh J, Pantel J. Train the trainer in dementia care. A program to foster communication skills in nursing home staff caring for dementia patients. Z Gerontol Geriatr. 2016;49(3):209-215.
38.Mayrhofer A, Goodman C, Smeeton N, Handley M, Amador S, Davies S. The feasibility of a train-the-trainer approach to end of life care training in care homes: an evaluation. BMC Palliat Care. 2016;15:11.
39.Sampson EL, Vickerstaff V, Lietz S, Orrell M. Improving the care of people with dementia in general hospitals: evaluation of a whole-system train-the-trainer model. Int Psychogeriatr. 2017;29(4):605-614.
70.de Zambotti M, Goldstone A, Claudatos S, Colrain IM, Baker FC. A validation study of Fitbit Charge 2 compared with polysomnography in adults. Chronobiology international. 2018;35(4):465-476.
71.Kolla BP, Mansukhani S, Mansukhani MP. Consumer sleep tracking devices: a review of mechanisms, validity and utility. Expert review of medical devices. 2016;13(5):497-506.
81.Colon-Emeric C, Toles M, Cary MP, Jr., et al. Sustaining complex interventions in long-term care: a qualitative study of direct care staff and managers. Implementation science : IS. 2016;11:94.
82.Chung F, Subramanyam R, Liao P, Sasaki E, Shapiro C, Sun Y. High STOP-Bang score indicates a high probability of obstructive sleep apnoea. British journal of anaesthesia. 2012;108(5):768-775.
83.Chung F, Yang Y, Brown R, Liao P. Alternative scoring models of STOP-bang questionnaire improve specificity to detect undiagnosed obstructive sleep apnea. Journal of clinical sleep medicine : JCSM : official publication of the American Academy of Sleep Medicine. 2014;10(9):951-958.
84.Chung F, Yegneswaran B, Liao P, et al. STOP questionnaire: a tool to screen patients for obstructive sleep apnea. Anesthesiology. 2008;108(5):812-821.
85.Smith MT, McCrae CS, Cheung J, et al. Use of Actigraphy for the Evaluation of Sleep Disorders and Circadian Rhythm Sleep-Wake Disorders: An American Academy of Sleep Medicine Systematic Review, Meta-Analysis, and GRADE Assessment. Journal of clinical sleep medicine : JCSM : official publication of the American Academy of Sleep Medicine. 2018;14(7):1209-1230.
86.Maglione JE, Liu L, Neikrug AB, et al. Actigraphy for the assessment of sleep measures in Parkinson's disease. Sleep. 2013;36(8):1209-1217.
87.Bellone GJ, Plano SA, Cardinali DP, Chada DP, Vigo DE, Golombek DA. Comparative analysis of actigraphy performance in healthy young subjects. Sleep science (Sao Paulo, Brazil). 2016;9(4):272-279.
88.Marino M, Li Y, Rueschman MN, et al. Measuring sleep: accuracy, sensitivity, and specificity of wrist actigraphy compared to polysomnography. Sleep. 2013;36(11):1747-1755.
97.Berk M, Ng F, Dodd S, et al. The validity of the CGI severity and improvement scales as measures of clinical effectiveness suitable for routine clinical use. Journal of evaluation in clinical practice. 2008;14(6):979-983.
98.Black JE, Hull SG, Tiller J, Yang R, Harsh JR. The long-term tolerability and efficacy of armodafinil in patients with excessive sleepiness associated with treated obstructive sleep apnea, shift work disorder, or narcolepsy: an open-label extension study. Journal of clinical sleep medicine : JCSM : official publication of the American Academy of Sleep Medicine. 2010;6(5):458-466.
99.Busner J, Targum SD. The clinical global impressions scale: applying a research tool in clinical practice. Psychiatry (Edgmont (Pa : Township)). 2007;4(7):28-37.
100.Dunlop BW, Gray J, Rapaport MH. Transdiagnostic Clinical Global Impression Scoring for Routine Clinical Settings. Behavioral sciences (Basel, Switzerland).

2017;7(3).
101.Dworkin RH, Turk DC, Farrar JT, et al. Core outcome measures for chronic pain clinical trials: IMMPACT recommendations. Pain. 2005;113(1-2):9-19.
102.Farrar JT, Young JP, Jr., LaMoreaux L, Werth JL, Poole RM. Clinical importance of changes in chronic pain intensity measured on an 11-point numerical pain rating scale. Pain. 2001;94(2):149-158.
103.Schneider LS, Olin JT, Doody RS, et al. Validity and reliability of the Alzheimer's Disease Cooperative Study-Clinical Global Impression of Change. The Alzheimer's Disease Cooperative Study. Alzheimer disease and associated disorders. 1997;11 Suppl 2:S22-32.
104.Teri L, Logsdon RG, Peskind E, et al. Treatment of agitation in AD: a randomized, placebo-controlled clinical trial. Neurology. 2000;55(9):1271-1278.
105.Boland J, Currow DC, Wilcock A, et al. A systematic review of strategies used to increase recruitment of people with cancer or organ failure into clinical trials: implications for palliative care research. Journal of pain and symptom management. 2015;49(4):762-772.e765.
106.Hunt KJ, Shlomo N, Addington-Hall J. Participant recruitment in sensitive surveys: a comparative trial of 'opt in' versus 'opt out' approaches. BMC medical research methodology. 2013;13:3.
107.Miller CJ, Burgess JF, Jr., Fischer EP, et al. Practical application of opt-out recruitment methods in two health services research studies. BMC medical research methodology. 2017;17(1):57.
108.Vellinga A, Cormican M, Hanahoe B, Bennett K, Murphy AW. Opt-out as an acceptable method of obtaining consent in medical research: a short report. BMC medical research methodology. 2011;11:40.
109.Richards K, Shue VM, Beck CK, Lambert CW, Bliwise DL. Restless legs syndrome risk factors, behaviors, and diagnoses in persons with early to moderate dementia and sleep disturbance. Behavioral sleep medicine. 2010;8(1):48-61.
110.Richards KC, Lambert C, Beck CK, et al. Strength training, walking, and social activity improve sleep in nursing home and assisted living residents: randomized controlled trial. J Am Geriatr Soc. 2011;59(2):214-223.
111.Rose KM, Beck C, Tsai PF, et al. Sleep disturbances and nocturnal agitation behaviors in older adults with dementia. Sleep. 2011;34(6):779-786.

c) **Provide stopping rules for the study, If the proposed study is a clinical trial where a drug, vaccine, device or other treatment is compared to a placebo group or comparison treatment group, what are the guidelines or endpoints by which early decisions regarding efficacy or lack of efficacy can be made? For example, it may be reasonable to stop enrollment on a study when efficacy has already been clearly demonstrated, to avoid unnecessary enrollments of additional participants. Alternatively, it may be reasonable to stop enrollment when it is clear that efficacy will never be demonstrated, given the statistical power of the study as designed. Describe the guidelines that are in place to assist in making these determinations, if relevant to the proposed study.**

We will exclude residents with a high risk of OSA who are not being treated for OSA because actigraph measurements are inaccurate in that population.85 Staff will also exclude residents who have a persistent bilateral resting tremor or paralysis in both arms (a subset of persons with Parkinson’s disease and related significant tremor-causing diagnoses), due to actigraph measurement inaccuracies.

d) **Describe how data analysis will be performed (statistical tests, methods of evaluating data) and indicate the smallest group/unit for which separate reporting will occur. For studies involving a questionnaire, if data and reliability information are available, please describe or provide references. (Page numbers from a sponsor's protocol/grant may be referenced in this section).**

Data analysis for this pilot study (R61 phase) will inform revisions to our upcoming randomized controlled trial (R33 phase). We will conduct a preliminary assessment of our outcome measures, particularly our primary outcomes from the actigraphy TST measurements and fitbit sleep time measurements, and secondary outcomes from the MDS and our supplementary sleep data (see Table 1). We will examine ranges, indicators of variability (change over time), and rates of missing data. We

will also examine indicators by demographic characteristics (e.g., age, gender) and level of dementia severity (mild, moderate, severe). To test the sensitivity of our secondary outcomes and supplementary sleep data to individual differences in TST and change in TST, we will preliminarily examine the relationships of these with average TST and variability in TST.

For the qualitative interviews, all interviews will be audio recorded and transcribed and Dr. McCullough will guide the experienced qualitative team in using a rapid appraisal template analysis,[112,113] a rigorous technique for thematically organizing and analyzing data. The analysis will identify, in particular, areas for modifications to the LOCK sleep program training. Interviews will also inform our decision of whether or not to include Fitbits as part of the subsequent randomized controlled trial (R33 phase), based on staff members' impressions of the value of Fitbits and their impressions of the feasibility of using both devices. We will also modify interview guides (see Appendix) for the next phase, as necessary.

[Selected References]
112.King N. Using templates in the thematic analysis of text. In: Essential Guide to Qualitative Methods in Organizational Research. London: SAGE Publications Ltd; 2004.
113.Beebe J. Basic Concepts and Techniques of Rapid Appraisal. Human Organization. 1995;54(1):42-51.

----

* * * Radioisotopes or Radiation Machines * * *

4) Radioisotopes or Radiation Machines

Please note: For projects requiring radiation procedures, please contact the UA Environmental Health and Safety Office at 348-6010

a) If applicable, summarize in lay language the radiographic diagnostic and therapeutic procedures associated with this protocol. (X-ray, fluoroscopy, CT, radioactive materials, nuclear medicine, PET-CT, radiation oncology, accelerator, Cyber Knife procedures, etc.).

b) Are the radiation procedures being performed a normal part of the clinical management for the medical condition that is under study (Standard of Care), or are the procedures being performed because the research participant is participating in this project (extra CT scans, more fluoroscopy time, additional Nuclear Medicine Studies, etc.) (Not Standard of Care)? If some procedures are Standard of Care and some are Not Standard of Care, check both boxes.

NOT STANDARD OF CARE	STANDARD OF CARE
If it is not standard of care,complete the rest of this section. Provide the University of Alabama RSC approval information below.	If it is only standard of care, skip the rest of this section.

c) Are research-related radiation procedures limited to X-rays only?

Yes (Complete X-ray table).

No (Skip X-ray table).

d) Total Radiation Exposure (in mRems) from x-ray procedures:

To calculate radiation exposure from x-rays only, University of Alabama allows use of the Duke University Radiation Safety Committee dose estimate calculator. University of Alabama does not allow use of this website to calculate any other type of radiation exposure.

To determine the dose estimate, click on the appropriate links, below (you will be taken to

the Duke University Radiation Safety Committee website). Enter the x-ray procedures into the appropriate fields of the website and click "create statement". Enter the dose estimate from the statement in the table above.

For studies involving adults, please click here.

For pediatric studies, please click here..

e) **Please list all radiation procedures (including x-ray) that are research-related (not standard of care). Include the anatomical location and specify the number of times that each procedure will be conducted throughout the entire study.**

NOTE: The IRB will determine if this study requires radiation safety review by the Radiation Safety Officer or the Radiation Safety Committee.

For more information on how to submit for radiation safety review, contact the Radiation Safety Officer.

----

* * * Drugs, Reagents, Chemicals, or Biologic Products * * *

5. Drugs, Reagents, Chemicals, or Biologic Products

Pilot	Phase I	Phase II
Phase III	Phase IV	Not Phased

a) Please list in the space below all investigational drugs, reagents or chemicals to be administered to participants during this study.

b) Please list in the space below all FDA approved drugs, reagents, chemicals to be administered to participants during this study.

Please read the IND Statement 1 and IND Statement 2.

----

* * * Devices * * *

6. Devices

a) Please list in the space below all investigational devices to be used on participants during this study.

b) Please list in the space below all FDA approved devices to be used on participants during this study.

----

* * * Subject Population(a-h) * * *

7. Subject Population - In the space below, please detail the participants that you are requesting to recruit (include description of each group requested)

a) **Expected age range of participants. (For example - 19 yrs to 90 yrs).**

In the potential participant pool, we will include all NH residents aged >=50 years with an ADRD diagnosis.

b)	**i) Number to be directly solicited for this research.**	N/A	all res. at 3 NHs
	**ii) Number to be consented (including withdrawals or screen failures)**	N/A	70
	**iii)Number expected to complete the study.**		57
c)	**If this is multi-center study, number of participants to complete the study study-wide**	N/A	57
d)	**If study involves review of medical or other records, number of records to be reviewed.**	N/A	70

e) **If women, minorities, or minors are excluded, a clear compelling rationale must be provided unless not applicable. Examples for not including minors: disease does not occur in children; drug or device would interfere with normal growth and development; etc.**

1. Inclusion of Women and Minorities
Participants will be selected according the inclusion/exclusion criteria described in the methods section. We aim to recruit a sample that will be representative of the national nursing home (NH) population. Based upon the 2015 Centers for Medicaid and Medicare Nursing Home Compendium,122 we will aim to enroll approximately 67% women and 33% men. The national figures for 2015 were: 79% White not Hispanic, 13.8% Black not Hispanic, 4.9% Hispanic, 1.6% Asian, .4% American Indian/Alaskan Native, .1% Native Hawaiian Pacific Islander, and .3% more than one race.
We will make every effort to include a representative sample of minorities. Men and members of minority groups will be actively recruited during this protocol.
We will use unbiased statistical analyses and proper methods of inference to estimate and compare intervention effects by sex/gender, race, and/or ethnicity. We will conduct exploratory analyses to detect any differences in intervention effect among these groups, because prior studies do not strongly indicate nor negate the existence of such effects.
2. Inclusion of Children
Children will not be included in this study. Children are unlikely to live or work in NHs and unlikely to have dementia.

f) **Describe how potential participants will be identified for recruitment (e.g., chart review, referral from individual's treating physician, those individuals answering an ad). How will potential participants learn about the research, and how will they be recruited (e.g., flyer, e-mail, web posting, telephone, etc.)? State where recruitment materials will be located. Click "Add" to upload recruitment materials document.**

Important to remember: Study Activities cannot begin until IRB approval is granted.

In the potential participant pool, we will include all NH residents aged >=50 years with an ADRD diagnosis. We include residents across the range of ADRD severity because this is consistent with the LOCK sleep program, in which staff will focus on residents with ADRD who have the greatest sleep problems without differentiating by ADRD severity. To identify participants, NH staff will use frontline staff huddles.

Research staff will recruit and consent a sample of NH staff at mid- and post-implementation for phone (mid-) and in-person (post-) interviews about their experiences with the LOCK sleep program and Fitbits. NOTE: The LOCK sleep program itself comprises clinical practices common in NH QI efforts. These practices thus fall within the scope of staff positions and require no consent.

---

* * * Subject Population(i-l) * * *

7. Subject Population (continued)

i) **Inclusion and Exclusion Criteria.**

Identify inclusion criteria.

In the potential participant pool, we will include all NH residents aged >=50 years with an ADRD diagnosis. We include residents across the range of ADRD severity because this is consistent with the LOCK sleep program, in which staff will focus on residents with ADRD who have the greatest sleep problems without differentiating by ADRD severity. To identify participants, NH staff will use frontline staff huddles. NH staff will be trained to use the STOP-Bang screening tool to identify NH residents with high risk of obstructive sleep apnea (OSA) 82-84 and will be trained on appropriate procedures for referring any positively screened residents for medical evaluation

Identify exclusion criteria.

We will exclude residents with a high risk of OSA who are not being treated for OSA because actigraph measurements are inaccurate in that population.85 Staff will also exclude residents who have a persistent bilateral resting tremor or paralysis in both

arms (a subset of persons with Parkinson's disease and related significant tremor-causing diagnoses), due to actigraph measurement inaccuracies.

j) **Compensation. Explain the amount and schedule of compensation, if any, that will be paid for participation in the study. Include provisions for prorating payment.**

N/A

k) **Describe who will cover study related costs. Explain any costs that will be charged to the participant.Include provisions for prorating payment.**

All study related costs will be covered by the NIA grant funding.

l) **Estimate the probable duration of the entire study including data analysis and publication. This estimate should include the total time each participant is to be involved and the duration the data about the participant is to be collected. If the study is Investigator-initiated, a timeline for individual participant recruitment, follow-up, total time for participant accrual, and data analysis for the study is required.**

The entire pilot study will last 1 year. Each NH resident participant will be involved in a 22 week data collection period.

----

* * * Subject Population(m) * * *

Research Involving Children

NOTE: Investigators, please include this information with the e-Protocol application if your research involves children. In Alabama a child is an individual less than 18 years of age unless the child is legally emancipated. If your research involves children with more than one vulnerability (e.g., children who are pregnant, incarcerated, or cognitively impaired) attach the supplementary information for that vulnerable population as well.

Minimal risk means that the probability and magnitude of the harm or discomfort anticipated in the research are not greater in and of themselves than those ordinarily encountered in daily life of a healthy child or during the performance of routine physical or psychological exams or tests.

Section 1.

Select and complete the category that applies to your research.

Category 1 (45 CFR 46.404; 21 CFR 50.51) My research does not involve greater than minimal risk.

a) My research falls under this category because:

b) Describe what provisions will be made for soliciting the assent of the children, and the permission of both parents, or the legal guardian. (Permission from both parents must be obtained unless one parent/guardian is deceased, unknown, incompetent, or not reasonably available, or when only one parent/guardian has legal responsibility for the care and custody of the child). Justify reason(s) if seeking permission from only one parent.

Category 2 (45 CFR 46.405; 21 CFR 50.52) My research involves greater than minimal risk but presents the prospect of direct benefit to the individual participants.

a) My research falls under this category because:

b) Justify the risk(s) by explaining the anticipated benefit to the participants:

c) Explain how the relation of the anticipated benefit to the risk is at least as favorable to the participants as that presented by available alternative approaches:

d) Describe what provisions will be made for soliciting the assent of the children, and the permission of at least one parent/guardian. (Permission from both parents must be obtained unless one parent/guardian is deceased, unknown, incompetent, or not reasonably available, or when only one parent/guardian has legal responsibility for the care and custody of the child). Justify reason(s) if seeking permission from only one parent.

Category 3 (45 CFR 46.406; 21 CFR 50.53) My research involves greater than minimal risk, and no prospect of direct benefit to individual participant, but likely to yield generalizable knowledge about the participant's disorder or condition.

a) My research falls under this category because:

b) Describe how the risks for participating in your research represent a minor increase over minimal risk (i.e., the children being recruited have a disorder or condition that would place them in a group other than an average healthy child; therefore, the research qualifies as a minor increment over minimal risk. This risk is slightly more than what the average healthy child would experience, but is reasonable for these participants because it is not more than they would experience or expect given their condition.).

c) Describe how the research intervention(s)/procedure(s) present experiences to participants that are reasonably commensurate to those inherent in their actual or expected medical, dental, psychological, social, or educational situations:

d) Explain why the intervention or procedure is likely to yield generalizable knowledge about the participants' disorder or condition, which is of vital importance for the understanding or amelioration of the participants' disorder or condition:

e) Describe what provisions will be made for soliciting the assent of the children, and the permission of both parents/guardians. (Permission from both parents must be obtained unless one parent/guardian is deceased, unknown, incompetent, or not reasonably available, or when only one parent/guardian has legal responsibility for the care and custody of the child). Justify reason(s) for seeking permission from only one parent.

Category 4 (45 CFR 46.407; 21 CFR 50.54) My research does not fall under Category 1, 2, or 3 listed above. However, the research presents a reasonable opportunity to understand, prevent, or alleviate a serious problem affecting the health or welfare of children.

(NOTE: If your research is funded by, or funding has been sought from the Department of Health and Human Services (DHHS), Department of Education, or is FDA regulated, a report must be sent for review to the DHHS Secretary, Secretary of the US Department of Education, or Commissioner of FDA. If this category is applicable, the Office of Research Compliance will prepare and submit a report of IRB review to the appropriate federal official(s)).

a) My research falls under this category because:

b) Describe what provisions will be made for soliciting the assent of the children, and the permission of both parents/guardians. (Permission from both parents must be obtained unless one parent/guardian is deceased, unknown, incompetent, or not reasonably available, or when only one parent/guardian has legal responsibility for the care and custody of the child). Justify reason(s) if seeking permission from only one parent.

Section 2.

In order to effectively assess and evaluate the risk of your proposed research to children, the IRB requires the following information. Respond to all items.

a) Provide justification for the participation of children as research participants in your study.

b) Has this research been conducted in adults?

If yes, is there any indication that the proposed research would benefit, or at least not be harmful to children?

c) Indicate how many children you propose to enroll in the study and justify this number (whenever possible, involve the fewest number of children necessary to obtain statistically significant data which will contribute to a meaningful analysis relative to the purpose of the study).

d) Describe how assent of a child will be obtained and documented (if applicable). If not applicable, explain why.

I am requesting waiver of the requirement for assent.

Justify:

OR

I have attached an assent form/assent script for IRB review.

e) Explain what methods will be used for evaluating dissent (i.e., description of behaviors that would indicate child does not want to participate (such as moving away, certain facial expressions, head movements, etc...).

f) Describe how parental permission will be obtained. [Note: If you propose to waive the requirement for parental permission (i.e., getting parental permission may be against the best interest of the child, i.e., a study of abused or neglected children), describe what measures will be taken to protect the rights and welfare of the children.]

I am requesting waiver of the requirement for parental permission.

Justify:

OR

I have attached a parental permission form for IRB review.

g) Describe measures that will be taken to ensure that a parent is present when the child participates in any research interventions or procedures. [Note: If the nature of the research is such that it is not appropriate to have a parent present (i.e., research into sensitive personal issues, physical examinations of teenagers, etc...) please explain why.]

h) Describe the expertise of the research staff/study personnel for dealing with children at the ages included and whether they are knowledgeable and sensitive to the physical and

psychological needs of the children and their families. Describe the appropriateness of facility in which the research will be conducted in relation to environment and/or equipment accommodating to children.

i) If applicable, provide any additional information that may support your request to involve children in this research.

*** Subject Population(n) ***

Research with cognitively impaired persons

NOTE: Investigators, please include this form with IRB application if your research involves cognitively impaired (decisionally impaired or decisionally challenged) persons. If your research involves people with more than one vulnerability, please complete the supplementary form for that population as well.

The IRB may ask you to designate an impartial observer to monitor the consent process or it may send its own representative to do so.

Section 1.

Note:Check the box next to the category that in your best judgment applies to your research, and provide the information requested in the space provided.

Note: Minimal risk means that the probability and magnitude of the harm or discomfort anticipated in the research are not greater in and of themselves than those ordinarily encountered in daily life or during the performance of routine physical or psychological exams or tests. (i.e., daily life of health persons)

X **Category 1 My research does not involve greater than minimal risk.**

Explain. If appropriate, describe what provisions are in place for allowing a Legally Authorized Representative (LAR) or other person with the participant's best interests at heart to assist the participant in navigating the research process:

As described in Section 2 sub-section #2 below, LARs will always be contacted and taken through a proxy consent process before NH residents with ADRD diagnoses are contacted.

Category 2 My research presents greater than minimal risk and prospect of direct benefit to the participants.

Explain. If appropriate, describe what provisions are in place for allowing a Legally Authorized Representative (LAR) or other person with the participant's best interests at heart to assist the participant in navigating the research process.

Category 3 My research presents greater than minimal risk and no prospect of direct benefit to the participants, but likely to yield generalizable knowledge about the participant's disorder or condition, because:

Explain. If appropriate, describe what provisions are in place for allowing a Legally Authorized Representative (LAR) or other person with the participant's best interests at heart to assist the participant in navigating the research process.

Category 4 My research does not fall under Category 1, 2, or 3, listed above.

If you check this category, the IRB determines additional safeguards on a case-by-case basis.

Section 2.

1. Explain why individuals with impaired decision-making capacity are suitable for this research. If the objective(s) of the study allow for inclusion of competent participants, provide compelling justification for inclusion of incompetent participants.

This study does involve the vulnerable population of nursing home residents with dementia. Sleep problems and related issues in NH residents with dementia is a well-

dementia. Sleep problems and related issues in NH residents with dementia is a well-documented problem. This study implements an intervention to improve the sleep experience for these residents. To evaluate the effects of the sleep program for people with dementia, people with dementia must be enrolled. If the LOCK sleep program is found to be effective in enhancing clinical care and outcomes, the approach can be shared with other NHs and incorporated into general NH practice. The risks in this study for NH residents are minimal, and the knowledge gained may result in improved quality of sleep, and ultimately improved quality of life for NH residents with dementia.

2. Describe who will determine individuals' competency to consent and the criteria to be used in determining competency (e.g., use of standardized measurements, consults with another qualified professional, etc...).

All consents as described below will be conducted by an IRB-approved study staff member who has been trained by Dr. Snow (principal investigator). The study staff member will be responsible for explaining the study, answering questions, and obtaining informed consent.

Cognitively Impaired NH Resident Consent – Step 1. Legally Authorized Representative Consent
NH leadership team [see Section #3B-Study Procedures] or their designees will identify all NH residents with an ADRD diagnosis and mail to their legally authorized representatives (LARs) an IRB-approved study introduction letter (including option to opt out within one month if they do not wish to be contacted by study staff). Opt-out procedures have been demonstrated to yield higher response rates and lower rates of non-response bias compared to opt-in procedures, and to be acceptable to participants, with no difference in rates of reported distress or complaints compared to opt-in procedures.[105-108] In our past federally funded research involving people with dementia, opt-out consent has been approved by multiple IRBs and worked well. Contact information of those LARs not opting out will be shared with University of Alabama (UA) study staff. We will request of the IRB a waiver of consent and HIPAA authorization for screening purposes regarding the sharing of these contact information.

Study staff will mail an IRB-approved study information packet via a tracked, express mailing service to all LARs who do not opt out. These materials will include a cover letter, an IRB-approved informed consent form (ICF), and an express mail return envelope so the LAR can send the signed ICF to the UA study office. LARs will be encouraged to contact study staff via phone or email for an appointment to discuss the study and go through the consent form together before signing the ICFs. Study staff will contact the LARs via phone and/or email within one week of sending the packet and will re-contact the LAR up to 6 times within the next month as needed to establish contact (if contact has not been established within one month, and NH staff are not able to determine that the LAR’s contact information has changed, then the lack of contact will be considered a soft refusal and no other contact will be attempted).

During a phone appointment, study staff will review all important elements of the ICF with the LAR, including the following: description of the study, information on how to contact the investigators and the NH administrator and director of nursing, a statement that there is no prejudice for refusal to participate, a statement that the subject may withdraw from the study at any time without prejudice, and statement regarding risk and benefit of participation. The LAR will be encouraged to ask questions. The LAR will be encouraged to call the PI if they have any remaining questions before signing the consent form.

Cognitively Impaired NH Resident Consent – Step 2. Screening for Capacity for NH Resident Consent/NH Resident Assent
Some NH residents with mild ADRD may have the capacity to provide their own consent. A two-step process will be used to address the ethical balance of the beneficence of

assuring that residents who do not have the capacity to consent are protected by LAR oversight with the promotion of autonomy for residents who do have capacity to consent. Once LAR consent is received for a resident, the NH leadership team will be asked to identify that resident's most recent MDS Brief Interview for Mental Status score[120] (BIMS; see Appendix for measure), a cognitive screening measure with good reliability and validity. For residents with BIMS scores in the mild range (above 10), the NH leadership team will be asked to arrange and support a telephone appointment between that resident and a study staff person (if the resident turns out to NOT have the cognitive ability to participate in the following telephone-based procedure, then they would also NOT have the cognitive capacity to provide informed consent, which requires higher level cognitive abilities).

In the telephone appointment, the study staff member will provide information about the study and will screen for capacity to consent by assessing the following. Does the resident respond appropriately to a social greeting? Is the resident oriented to place? Does the resident understand who the study team member is after the team member explains their identity? During the course of this introductory conversation, was the resident able to respond fluently and socially appropriately? If so, the team member will go through the consent form with them. Residents will then be asked a series of questions to assure their understanding. These questions are as follows:
Describe in your own words what this study is about?
Describe what I am asking you to do to participate in this study?
Are you required to do this interview with me?
What will happen if you don't talk to me?
What happens if you decide you don't want to participate once we get started?
What will I do with the information you give me?

The NH leadership team member will support this telephone conversation by assuring that the resident has a quiet environment to have the telephone conversation and that the resident has in front of them a printed large-font version of the ICF with the signature area flagged. If the resident is able to respond appropriately to all the capacity screening questions, and they do wish to participate, then the resident will be instructed to sign the ICF. The study staff person will contact the NH leadership team member and ask them to make a copy of the ICF for the resident to keep, place a copy in the medical chart, and express mail the original to the UA study office using a provided pre-paid envelope. If the resident is able to respond appropriately to all the capacity screening questions and does not wish to participate then they will not be enrolled in the study, regardless of the LAR's consent. In this case, the study team member will let the NH leadership team and the LAR know that the resident has declined participation.

Under all circumstances, the NH resident's autonomy will be respected. For NH residents without capacity to consent, their assent to participate will be obtained whenever possible, and their decision to withdraw at any time (whether expressed verbally or by resistance to participation) will be honored (see also staff training in Section #3B-Study Procedures).

All above procedures apply to securing consent for the research study team to have access to NH resident personally identifiable data.

NOTE: the LOCK sleep program does not include any activities that are outside of standard NH practice, therefore NH staff will be free to enroll any NH residents that they believe will benefit into the LOCK sleep program regardless of resident consent status; however the NH leadership team will only be allowed to share with the research study team data for residents who are consented for the study.

3. It should be recognized that decision-making capacity may fluctuate, requiring ongoing assessment during the course of the research. Is it reasonable to expect that during Y

the course of the research, subjects may lose their capacity to consent or their ability to withdraw?

a) Describe what provisions are in place for periodic re-consent. Include the rationale and procedure, the proposed interval, any changes in behavior that might signal the need to re-consent whether or not the proposed interval has elapsed, and any consultative resources that are available for these decisions. Describe the process for re-consent or re-assent, or reassessment of willingness to continue participation.

Re-consenting provisions will not be included because the course of the study is a relatively short 22 weeks (5.5 months), an amount of time in which it is unlikely, although possible, that cognitive capacity will significantly change. Further, the main focus of the intervention is with staff behavior. Staff will be implementing interventions that are considered good standard clinical practice, such as providing meaningful activities during the day to help reduce resident daytime napping and reducing noise and resident interruptions at night to promote uninterrupted sleep. Therefore, is is unlikely, although possible, that resident willingness to continue participation will change over this relatively short amount of time.<br><br>The most likely cause of resident unwillingness to continue participation would be over the wearing of the actigraph and/or fitbit. NH staff will be trained by their NH leadership teams or their trained designees (who will have been trained by Dr. Snow or her study team designees) on proper use and placement of actigraphs and Fitbits, verbal and behavioral signs of<br>distress that might indicate actigraph/Fitbit-related discomfort/distress [e.g., verbal indicators such as words (e.g., "take it off!"), distress sounds (e.g., crying, moaning, groaning), physical movements (e.g., picking at the device, shaking the arm)], and appropriate modification techniques to try to relieve such distress. NH staff will be informed that the devices should be removed in the unlikely event that distress behaviors are not relieved by modification techniques. NH staff will be trained on importance of NH resident autonomy, and will be engaged in a discussion of how to assess for and honor NH<br>resident assent or lack thereof with regard to the devices.

b) Describe what provisions are in place to protect the participants' rights in the event they lose their capacity to consent or their capacity to withdraw during the course of the research. (e.g., power of attorney, consent a caregiver as well as the patient, etc.).

Most residents will not have the capacity to consent. As described above, behavioral indicators of withdraw of assent will be honored and NH staff will be carefully trained to recognize these indicators. In addition, LARs will have the study team's contact information and a copy of their consent form which clearly indicates how they can reach the study team and withdrawn consent at any time. These procedures will be reviewed with LARs as part of the consent process.

c) Describe what provisions are in place for use of additional waiting periods to allow potential participants time to consult with family members about whether or not to participate.

Most residents will not have the capacity to consent, and LARs will be consenting. LARs will always be contacted about potential participation before the NH resident is contacted. As described above, if it becomes apparent that the NH resident is able to consent for themselves, their decision will be honored even if it is discrepant from that of the LAR (i.e., if LAR says yes but resident decides no).

4. Explain how you will identify who is authorized to give legally valid consent on behalf of any individual(s) determined to be incapable of consenting on their own behalf.

We will be guided by the medical chart documentation of who is identified as the legally authorized representative (LAR), combined with guidance from the nursing home staff

who know the NH resident and family the best regarding who is the appropriate LAR.

5. Explain the criteria you will use for determining when assent is required for participants who are not competent.

We will always look for assent for participants who are not competent to consent. The behavioral indicators of lack of assent for the actigraph/fitbit are described above.

There are no study self-report data collected from residents. All resident data involved in this study are data that the NH collects as part of their standard clinical practices (e.g., Minimum Data Set (MDS) data that is mandated data collected as per Medicare/Medicaid reimbursement rules). All other resident involvement is concordant with clinical best practice. The main focus of the intervention is with staff behavior. Staff will be implementing interventions that are considered good standard clinical practice, such as providing meaningful activities during the day to help reduce resident daytime napping and reducing noise and resident interruptions at night to promote uninterrupted sleep.

6. Explain what methods will be used for evaluating dissent (e.g., description of behaviors that would indicate individual does not want to participate (such as moving away, certain facial expressions, head movements, etc...).

Verbal and behavioral signs of distress that might indicate actigraph/Fitbit-related discomfort/distress include: verbal indicators such as words (e.g., "take it off!"), distress sounds (e.g., crying, moaning, groaning), and physical movements (e.g., picking at the device, shaking the arm)].

7. The research protocol should include someone who can be reasonably assumed to have the participant's best interest in mind and can assist the participant in navigating the consent and research process. A person holding durable power of attorney or other legal designee, spouse, close relative who is involved in ongoing care of participant, other person with a personal or blood relationship who is involved in ongoing care of participant, or other close relatives or friends may assume this role. Describe how individuals will be identified to serve in this capacity. If this request is not appropriate for this study, justify why it should be waived.

As described in #4 above, we will be guided by the medical chart documentation of who is identified as the legally authorized representative (LAR), combined with guidance from the nursing home staff who know the NH resident and family the best. LARs will always be contacted about potential participation before the NH resident is contacted.

8. If applicable, describe when and how the individual's health care provider will be consulted prior to participation in the research. NOTE: If the Principal Investigator (PI) is also the individual's health care provider, address how the PI will separate the roles of clinician and researcher.

The medical director of the nursing home will be consulted before NH residents are enrolled in the study. By law, NH residents may use any physician they would like and therefore the medical director may not be their personal physician, but would still have medical oversight over their well-being and would be able to offer recommendations if for any reason a particular resident had contraindications for participation. Similarly, the director of nursing and the entire NH clinical interdisciplinary team will be consulted before NH residents are enrolled in the study, providing adequate opportunity for any clinical team member to indicate if participation of a particular resident would be contraindicated.

9. Will the research interfere with current therapy or medications? N

If yes, describe what the changes may entail (i.e., if the subjparticipantl be removed from routine drugs/treatments, wash out periods, etc.) and the potential risks.

10. Does your research involve institutionalized individuals? Y

a) Justify the use of institutionalized individuals and explain why non-institutionalized individuals can not be substituted.

This is a study of how to improve nursing home treatment. Because the factors that affect nursing home treatment are systemic, investigation of interventions to improve nursing home treatment can only occur within the system of the nursing home itself.

Section 3.

Complete this section if your research involves individuals from the Department of Veterans Affairs (VA).

1. Address procedures you will use to ensure the participant's representative is informed regarding his/her role and obligation to protect the incompetent participant or person with impaired decision-making capacity.

N/A

2. Address procedures you will use to ensure the participant's representative has been told of his/her obligation to try to determine what the prospective participant would do if competent, or if the prospective participant's wishes cannot be determined, what the participant's representative thinks is in the incompetent person's best interests:

N/A

3. The VA has specific requirements and procedures for determining and documenting in the person's medical record that an individual is incompetent or decisionally-impaired. There are additional requirements if the lack of decision-making capacity is based on diagnoses of mental illness. These requirements are outlined in the Veterans Health Administration (VHA) Handbook 1200.5, Section II. Have you reviewed these requirements and included them in your procedures? N

4. Justify that the research involves no significant risks, or if the research presents probability of harm, justify that there is at least a greater probability of direct benefit to the participant:

N/A

Note:[Veterans Health Administration Handbook 1200.5, July 15, 2003, Section 11 - Research Involving Human participants with Surrogate Consent, and Appendix D Vulnerable Populations, Section 6(c)]

Section 4.

For research involving cognitively impaired persons outside the state of Alabama, also complete this section.

a) Provide information regarding the state definition of legally authorized representative, child, decisionally-impaired, or guardian, as applicable to the research and to the federal definitions. [If the research is to be conducted in more than one state outside of Alabama, provide this information for each state.]

States that will be involved have not yet been fully determined. Recruitment of Nursing Home corporations is underway. An amendment to this protocol will be submitted when the all nursing home corporations have been recruited and the specific names of the participating nursing homes and their states are known.

Definitions:

Assent - is defined as a child's or decisionally-challenged individual's affirmative agreement to participate in research. Mere failure to object should not, absent affirmative agreement, be construed as assent.

Competence "Technically, a legal term, used to denote capacity to act on one's own behalf;

the ability to understand information presented, to appreciate the consequences of acting (or not acting) on that information, and to make a choice." [OHRP Institutional Review Board Guidebook, Chapter VI, Section D]

Permission is defined as the agreement of parent(s) or guardian to the participation of their child or ward in research or clinical investigation. Permission includes the element of consent set forth in federal regulations and outlined in the informed consent template included in the IRB expedited and full review applications.

In Alabama child/children refers to all individuals less than 18 years of age unless the individual(s) is/are legally emancipated. (See Guidance: Alabama Law on Children, Minors, Consent, and Other Research-Related Topics. Individuals less than 18 years of age who are not emancipated meet the federal definition for "child" (e.g., Department of Health and Human Services (DHHS), Food and Drug Administration (FDA), and U.S. Department of Education).

Legally authorized representative (LAR) is an individual who has the authority to make research participation decisions on behalf of another. Alabama law does not specify who may make such decisions. UA legal counsel recommends the following in this order of preference: A legally appointed guardian, a health care proxy or person authorized to make medical decisions in conjunction with a durable power of attorney, a spouse, an adult child, next of kin, or a person or agency acting in loco parentis.

NOTE: Consent from a legally authorized representative involves all the ethical and regulatory concerns that apply to consent from the prospective participant.

----

* * * Subject Population(o) * * *

Pregnant Women, Fetuses and Neonates

NOTE: Investigators, please include this information with the e-Protocol application. Check the box that best fits your research and address the issues that immediately follow as they apply to your research. If your research involves women with more than one vulnerability (e.g. , pregnant women who are children under Alabama law or pregnant women who are cognitively impaired), attach the supplementary application form for that population as well.

Note: Definition of Minimal Risk: The probability and magnitude of harm or discomfort anticipated in the research are not greater in and of themselves than those ordinarily encountered in daily life or during the performance of routine physical or psychological exams or tests.

Research Involving Pregnant Women or Fetuses [45 CFR 46.204]

A. Explain why the proposed research is scientifically appropriate, including descriptions of any pre-clinical studies on pregnant animals and any clinical studies conducted on non-pregnant women that have provided data for assessing potential risks to pregnant women and fetuses.

B. Check the box next to the item that best describes the anticipated risk to the fetus:

1. Not greater than minimal
2. Greater than minimal risk and the risk to the fetus is caused solely by interventions or procedures that hold out the prospect of direct benefit for the woman or the fetus.

C. Provide a rationale for the anticipated risk:

D. Explain why any risk is the least possible for achieving the objectives of the research:

E. Check the appropriate box as it applies to this research:

1. This research holds out the prospect of a direct benefit to the pregnant woman.

2. This research holds out the prospect of direct benefit both to the pregnant woman and the fetus.

3. This research does not hold out the prospect of a direct benefit for the woman or the fetus, but the risk to the fetus is not greater than minimal, and the purpose of the research is the development of important biomedical knowledge that cannot be obtained by any other means.

Note: If "Yes" to any of the above in "E", informed consent must be obtained from the pregnant women or her legally authorized representative (LAR) as required in 45 CFR 46.116 & 117, but consent from the father is not required. The informed consent process should include a clear explanation of the reasonably foreseeable impact of the research on the fetus.

4. This research holds out the prospect of a direct benefit solely to the fetus.

Note: If "yes", informed consent must be obtained from the pregnant woman and the father as required in 45 CFR 46.116 & 117. The informed consent process should include a clear explanation regarding the reasonably foreseeable impact of the research on the fetus. NOTE: The father's informed consent need not be obtained if he is unable to consent because of non-availability, incompetence or temporary incapacity or if the pregnancy resulted from rape or incest.

5. This research will involve individuals under the age of 19 who are pregnant and are not considered emancipated minors.

Note: If "Yes", assent from the pregnant child and permission from her parent or legal guardian must be obtained in accordance with the provisions of 45 CFR 46, Subpart D.

6. Will there be any inducements, monetary or otherwise, offered to terminate a pregnancy?

7. Will individuals performing research procedures have any part in any decisions as to the timing, method, or procedures used to terminate a pregnancy?

8. Will individuals performing research procedures have any part in determining the viability of a fetus?

Note: "Yes" answers to 6-8 mean that the research cannot be approved.

Section 2. Research Involving Neonates [§ 46.205]

A. Neonates of Uncertain Viability AND Nonviable Neonates - Until it has been ascertained whether or not a neonate is viable, a neonate may not be involved in research covered by 45 CFR 46 Subpart B unless the IRB determines that the following conditions are met.

1. Explain why the proposed research is scientifically appropriate and provide a description of any pre-clinical and clinical studies that have been conducted which provide data for assessing potential risks to neonates.

2. Will individuals engaged in the research have any part in determining the viability of a neonate?

Note: A "Yes" answer means that the research cannot be approved. Individuals engaged in the research may have no part in any decisions as to the timing, method, or procedures used to terminate a pregnancy.

3. Is any inducement, monetary or otherwise, offered to terminate a pregnancy?

Note: A "Yes" answer means that the research cannot be approved. No inducements, monetary or otherwise, may be offered to terminate a pregnancy.

B. Neonates of Uncertain Viability - Additional Requirements. Check if applicable.

1. The research holds out the prospect of enhancing the probability of survival of the neonate to the point of viability, AND any risk is the least possible for achieving that objective, or

2. The research has the main purpose of the development of important biomedical knowledge, which cannot be obtained by other means AND there will be no added risk to the neonate resulting from the research.
3. Explain the procedures that will be used to obtain legally effective informed consent of either parent of the neonate.

NOTE: If neither parent is able to consent because of unavailability, incompetence, or temporary incapacity, the legally effective informed consent of either parent's LAR will be obtained as required by 45 CFR 46.116 & 117. These procedures must assure that each individual providing informed consent will be fully informed regarding the reasonably foreseeable impact of the research on the neonate. The father's informed consent need not be obtained if he is unable to consent because of non-availability, incompetence or temporary incapacity or if the pregnancy resulted from rape or incest.

C. Nonviable Neonates - Additional Requirements. After delivery a nonviable neonate may not be involved in research covered by 45 CFR 46 Subpart B unless the IRB determines that the following additional conditions are met. Please check if applicable to your research.

1. Will the vital functions of the neonate be artificially maintained?
2. Does the research include procedures to terminate the heartbeat or respiration of the neonate?
3. Will there be any added risk to the neonate from this research?

"Yes" answers to 1-3 mean that the research cannot be approved.

4. Is the sole purpose of the research for the development of important biomedical knowledge that cannot be obtained by other means?

If "Yes", please explain:

5. Explain the procedures that will be used to obtain legally effective informed consent of both parents of the neonate.

Note: If either parent is unable to consent because of unavailability, incompetence, or temporary incapacity, the informed consent of one parent of a nonviable neonate will suffice. The consent of the father need not be obtained if the pregnancy resulted from rape or incest. The consent of a legally authorized representative of either or both of the parents of a nonviable neonate will NOT suffice. These procedures must assure that each individual providing informed consent will be fully informed regarding the reasonably foreseeable impact of the research on the neonate.

D. **Viable Neonates - A neonate, after delivery, that has been determined to be viable may be included in research only to the extent permitted by and in accordance with the requirement of 45 CFR 46 Subparts A and D. The neonate is now a child. Please follow policies and consenting process for research with children and attach FORM: APPLICATION FOR RESEARCH WITH CHILDREN.**

Section 3. Research Involving After Delivery, The Placenta, The Dead Fetus, or Fetal Material [§ 46.206]

A. This research proposes to use the following: (Check all that apply)

Placenta

The dead fetus

Macerated fetal material

Cells excised from dead fetus

Tissue excised from dead fetus

Organs excised from dead fetus

Other(Describe)

Note: The use of any of the above must be conducted in accordance with any applicable Federal, State, or local laws, regulations, and institutional policies regarding such activities.

B. Will any information associated with the above material be recorded for research purposes in such a manner that living individuals can be identified directly or through identifiers linked to those individuals?

If "Yes", provide a rationale for the recording of identifiable information.

Note: These individuals are considered to be research participants and all pertinent human participant regulations are applicable to their participation.

Section 4. Research Not Otherwise Approvable Which Presents an Opportunity to Understand, Prevent, or Alleviate a Serious Problem Affecting the Health or Welfare of Pregnant Women, Human Fetuses, or Neonates [§ 46.207, § 46.208, §46.209, § 46.210]

A. If the study is Department of Health and Human Services (HHS) funded, or if funding by HHS is sought, review by the Secretary of HHS and posting in the Federal Register for public comments and review is required. If this category is applicable, the Research Compliance Office will prepare and submit a report of IRB review to the appropriate HHS institutional official.

---

* * * Subject Population(p) * * *

Research Involving Prisoners

NOTE: Investigators, please include this information with the e-Protocol application if your research involves prisoners. This includes studies of known prisoners and studies recruiting participants at risk of becoming involuntary prisoners, such as participants with histories of substance abuse. Remember that persons involuntarily committed to mental health facilities (Taylor Hardin Secure Mental Health Facility, Mary Starke Harper, etc.) by the courts are also prisoners.

If participants unexpectedly become prisoners, go directly to SECTION FOUR of this form.

If your research involves prisoners with more than one vulnerability (i.e., prisoners who are also children or pregnant, are involuntarily committed to mental health facilities), attach the supplementary form for that vulnerable population as well.

Regardless of the category of your research, be sure that your application makes clear why the research must be done on prisoners.

Indicate the category that best represents your research by checking the applicable box below, and explain in the space provided for that category why your research meets the criteria.

Note: For research involving prisoners, the definition of minimal risk refers to the probability and magnitude of physical or psychological harm that is normally encountered in the daily lives, or in the routine medical, dental or psychological examination of healthy persons.

Category 1 (45 CFR 46.306(a)(2)(i))

My research involves the study of possible causes, effects, processes of incarceration, and of criminal behavior. (Processes of incarceration can be interpreted broadly to include substance abuse research, half-way houses, counseling techniques, criminal behavior, etc.)

Justify how the research presents no more than minimal risk and no more than inconvenience to the participants:

Category 2 (45 CFR 46.306(a)(2)(ii))

My research involves the study of prisons as institutional structures, or of prisoners as incarcerated persons. (This category is usually used fairly narrowly as when looking at prisoner diet and conditions of prison life.)

Justify how the research presents no more than minimal risk and no more than inconvenience to the participants

Category 3 (45 CFR 46.306(a)(2)(iii))

My research involves the study of conditions particularly affecting prisoners as a class. (This category is less frequently used than the previous ones and refers to such research as vaccine trials, research on hepatitis, and social and psychological problems such as alcoholism, drug addiction, and sexual assaults. Minimal risk studies should not go under this category.) For DHHS-funded research, OHRP has consulted with appropriate experts including experts in penology, medicine, and ethics, and published notice, in the Federal Register, of its intent to approve such research.

Note: Contact the Office of Research Compliance at (205) (348-8461 for more information

Explain what condition(s) will be studied and provide rationale for each:

Category 4 (45 CFR 46.306(a)(2)(iv))

My research involves the study of practices, both innovative and accepted, which have the intent and reasonable probability of improving the health or well-being of the participant. (Note: It is rare for research involving placebo or control groups to fall in this category because of the difficulty in justifying improvement of the health or well-being of the participant being given placebo or in a control group.) For DHHS-funded research which requires the assignment of prisoners in a manner consistent with protocols approved by the IRB to control groups which may not benefit from the research, the study may proceed only after OHRP has consulted with appropriate experts, including experts in penology, medicine, and ethics, and published notice, in the Federal Register, of its intent to approve such research.

Note: Contact the Office of Research Compliance at (205) 348-8461 for more information.

Explain the research practices that will be used in this study and how they are intended to improve the health and well-being of the participants:

Section 2. [45 CFR 46.305]

Note: When an IRB is reviewing a protocol in which a prisoner will be a participant, the IRB must find and document justification that six additional conditions are met. Describe in the space provided how each condition applies to your research.

1. Advantages acquired through participation in the research, when compared to the prisoners' current situation, are not so great that they impair their ability to weigh risks.

   Describe the possible advantages that can be expected for prisoner participants:

2. Risks are the same as those that would be accepted by non-prisoners.

   Describe the possible risks that can be expected for prisoner participants and justify that they are the same as for non-prisoners:

3. Procedures for selection are fair to all prisoners and are immune from intervention by prison authorities in prisons; control participants must be randomly selected.

   a) **Describe how prisoners will be selected for participation:**

   b) **Describe what measures will be taken to prevent intervention by prison authorities in the selection process:**

4. Parole boards cannot take into consideration a prisoner's participation in research. Informed consent must state participation will not affect length of sentence or parole.

5. For studies that require follow-up, provisions are made including consideration for the length of individual sentences; informed consent must reflect provisions for follow-up.

Describe what provisions have been made for follow-up and how this information will be relayed to the prisoner participants:

6. Information about the study is presented in a language understandable to prisoners.

Describe what efforts have been made to present information about the study in a language that is understandable to the prisoner population. This may mean a non-English language or an appropriate reading level in whatever language the prisoner uses.:

Section 3. Only complete if applicable: Epidemiologic Research Involving Prisoners and Funded by the Department of Health and Human Services (DHHS)

Note: Effective June 20, 2003, DHHS adopted policy that allows waiver of the requirement for documenting applicability of a 45 CFR 306(a)(2) category (as found in Section 1 of this form) for certain epidemiologic research involving prisoners. This waiver applies to DHHS conducted or supported epidemiologic research on prisoners that presents no more than minimal risk and no more than inconvenience to the prisoner-participants.

Check the box below if your research meets the listed criteria, then provide justification in the space provided.

1. My research is funded by HHS and I request a waiver for meeting the category conditions under Section 1 of this form.
2. My research involves epidemiologic research intended to describe the prevalence/incidence of a disease by identifying all cases, or to study potential risk factor associations for a disease; and
3. Prisoners are not the sole focus of my research.

Justify how the research presents no more than minimal risk and no more than inconvenience to the participants:

Section 4. Complete if applicable:

Prisoners are not the targeted population

Note: Although prisoners may not be the target population for your research, a participant could become a prisoner during the course of the study (particularly if studying a subject population at high-risk of incarceration).

Note: If you did not receive IRB approval for involvement of prisoners, and a participant becomes a prisoner during the study, all research interactions and interventions with, and obtaining identifiable private information about, the now-incarcerated participant must cease until IRB approval has been issued for their continuation in the research. If you need IRB approval for a prisoner participant to continue participation in your research, select and complete the applicable category from Section 1, complete section 2 and this section, then submit for IRB review.

In special circumstances in which the Principal Investigator asserts that it is in the best interest of the participant to remain in the research study while incarcerated, the IRB Chairperson may determine that the participant may continue to participate in the research prior to satisfying the requirements of Subpart C. However, subsequent IRB review and approval of this completed form, documenting that the requirements of Subpart C are met, is required.

Prisoners are not a target population for my research, but a participant became a prisoner during the study and I am seeking IRB approval so the participant can continue

during the study and I am seeking IRB approval so the participant can continue participation in the research.

Explain the importance of continuing to intervene, interact, or collect identifiable private information during the participant's incarceration:

Note: Prisoner: An individual involuntarily confined in a penal institution, including persons: (1) sentenced under a criminal or civil statue; (2) detained pending arraignment, trial, or sentencing; and (3) detained in other facilities (e.g., for drug detoxification or treatment of alcoholism,) under statutes or commitment procedures providing such alternatives to criminal prosecution or incarceration in a penal institution [45 CFR 46.303(c)]. Note: Persons on Probation and parole are usually NOT considered to be prisoners.

If you will receive or are seeking Department of Health and Human Services (HHS) funding for this study, a certification letter must be submitted to the Office for Human Research Protections (OHRP). The research cannot be initiated until OHRP issues approval. The Office of Research Compliance (ORC) will prepare and submit the certification report to OHRP. Contact the Director for the Office of Research Compliance at 205-348-8461 8641 for more information.

----

* * * Risks * * *

8. Risks

There is no research that can be considered totally risk free (e.g., a potential risk of breach of confidentiality). Therefore, when describing the risk, the lowest level of risk is "no more than minimal risk".

a) For the following categories include a scientific estimate of the frequency, severity, and reversibility of potential risks. Wherever possible, include statistical incidence of complications and the mortality rate of proposed procedures. Where there has been insufficient time to accumulate significant data on risk,a statement to this effect should be included. (In describing these risks in the consent form to the participant, it is helpful to use comparisons which are meaningful to persons unfamiliar with medical terminology).

Address any risks related to (input N/A if not applicable):

1. Use of investigational drugs. Please include the clinical adverse events (AEs) associated with each of the drugs with an indication of frequency, severity and reversibility. This information can often be found in the Investigator(s) brochure. NOTE: Include any likely adverse effects associated with placebos or washout periods that participants may experience while in the study.

   N/A

2. Use of investigational devices. Please include the clinical adverse events (AEs) associated with each of the devices with an indication of frequency, severity and reversibility. This information can often be found in the Investigator(s) brochure. NOTE: Include any likely adverse effects associated with procedures that participants may experience while in the study.

   No investigational devices will be used.

3. Use of FDA approved drugs, reagents, chemicals, or biologic products. Please include the clinical adverse events (AEs) associated with each of the drugs with an indication of frequency, severity and reversibility. This information can often be found in the package insert provided by the manufacturer. NOTE: Include any likely adverse effects associated with placebos or washout periods that participants may experience while in the study.

   N/A

4. Use of FDA approved devices. Please include the clinical adverse events (AEs) associated with each of the devices with an indication of frequency, severity and reversibility. This information can often be found in the Investigator(s) brochure. NOTE: Include any likely adverse effects associated with procedures that participants may

experience while in the study.

N/A

5. Describe any risks related to performing study procedures. Please include all investigational, non-investigational, and non-invasive procedures (e.g., surgery, blood draws, treadmill tests).

No more than minimal risk.

Regarding psychological risks to NH residents, the LOCK sleep program including frontline huddling provides a sequence of steps for the process of assessing and responding to sleep problems. During the intervention period NH residents enrolled in the LOCK sleep program will receive actigraph and Fitbit assessment, and although our teams' experience with use of these devices in hundreds of people with dementia indicates that they are widely tolerated as comfortable, there is a small risk a resident may find the devices to be uncomfortable or otherwise distressing. As part of their intervention training, staff will be instructed on behavioral signs of distress. If a participant exhibits significant behavioral distress due to assessment, staff will be instructed on modifications and alternatives. If the distress does not subside, staff will be instructed to discontinue the assessment.

6. Describe any risks related to the use of radioisotopes/radiation-producing machines (e.g., X-rays, CT scans, fluoroscopy).

N/A

7. For clinical studies (of a drug, vaccine, device or treatment), describe any alternative procedure(s) or course(s) of treatment. List important risks and benefits of these alternatives in order to compare to study procedure(s) or course(s) of treatment. This information MUST be included here. Any standard treatment that is being withheld must be disclosed and the information must be included in the consent form.

The intervention described will be implemented NH-wide. The study is a pilot (R61) for the methods for a subsequent randomized controlled trial (R33 -- an incomplete stepped-wedge cluster randomized controlled trial design in which each NH serves as its own control). There will therefore be no alternative treatments. NH residents and their legally authorized representatives will have the right to refuse participation in any portion of their care just as they would with usual NH care, including use of actigraphs and Fitbits. NH staff will have the right to refuse any interviews.

8a. Describe any other physical, psychological, social or legal risks the participant may experience.

No more than minimal risk.

The risks for participants in this trial are minimal. There are no anticipated physical or economic risks associated with this study. There are no psychological risks beyond what was described in Risks sub-item #5 above.

For both NH residents and staff, an unlikely legal or social risk associated with participating in this study would be invasion of privacy in the remote chance that there is a loss or lack of protection of data containing personally identifiable information. These are research risks, not therapeutic risks. The study team will use careful procedures to guard the security and confidentiality of all data.

For both NH residents and staff, a potential risk is that a person may not want to participate in the study and may feel coerced to do so. Therefore, to minimize this type of risk, all informed consent contact with potential participants will come through the study staff only, and all NH staff participation discussions and decisions will be kept confidential.

8b. Data Safety Monitoring

Is there a Data Monitoring Committee (DMC) or Board (DSMB)? Y

If yes, describe its role, if it is independent of the sponsor or research team, the make-up of the Board and their qualifications, and how often the Board will meet.

1 Frequency of Data and Safety Monitoring
The Principal Investigator (PI) will be responsible for ensuring participants' safety on a daily basis. The Data and Safety Monitoring Board will meet at least once a year by teleconference (and more often as needed). The DSMB will act in an advisory capacity to the NIA Director by monitoring participant safety; evaluating the progress of the study; and reviewing procedures for maintaining the confidentiality of data and the quality of data collection, management, and analyses.

2 Content of Data and Safety Monitoring Report
The content of the data and safety monitoring report will include: protocol synopsis, study status, summary of past DSMB meetings, recruitment and participant status, data quality status, and safety information. Blinded reports will be produced for open sessions and unblinded reports for closed sessions. The DSMB will be guided by the NIA DSMB Report Template.

3 DSMB Membership and Affiliation
All members of the DSMB will have extensive experience in the conduct and analysis of clinical trials and/or clinical expertise in the study population. The committee will be named in conjunction with the NIA Program Officer. The board will comprise a physician with geriatrics or nursing home (NH) expertise, a nurse scientist with expertise in geriatrics or NHs, a behavioral clinician with expertise in geriatrics or NHs, a biostatistician, and an additional clinician scientist with sleep expertise. They will be completely independent of the study investigators and staff and have no scientific, financial, or other conflict of interest with the study.
DSMB membership will be reviewed and approved by the NIA. Should there be any questions regarding the independence of the DSMB, it will be addressed and corrected if necessary at that time.

4 Conflict of Interest for DSMB
Each DSMB member will sign a Conflict of Interest Statement which includes current affiliations, if any, with pharmaceutical and biotechnology companies (e.g., stockholder, consultant), and any other relationship that could be perceived as a conflict of interest related to the study and / or associated with commercial interests pertinent to study objectives.

5 Protection of Confidentiality
Data will be presented in a blinded manner during the open sessions of the DSMB. At DSMB meetings, data and discussion are confidential. Participant identities will not be known to the DSMB members.

6 DSMB Responsibilities and Procedures
The DSMB will be responsible for reviewing the research protocol and informed consent documents, and developing plans for data safety and monitoring. They will evaluate the progress of the trial, including periodic assessments of data quality and timeliness, recruitment, accrual and retention, participant risk versus benefit, performance of the trial sites, and other factors that can affect study outcome; consider factors external to the study when relevant information becomes available, such as scientific or therapeutic developments that may have an impact on the safety of the participants or the ethics of the trial; review study performance and make recommendations; and assist in the resolution of problems reported by the Principal Investigator (PI). The DSMB will report on the safety and progress of the trial to the NIA Program Officer and make

recommendations to the Program Officer and the PI concerning continuation, termination, or other modifications of the trial based on the observed beneficial or adverse effects. They will ensure the confidentiality of the results by monitoring all study data and analysis.

The DSMB will meet at least once a year, or more frequently as determined by the board at the first meeting. Emergency meetings also may be called at any time by the DSMB Chairperson or by NIA. A majority of members must be present for called meetings in order to constitute a quorum. The NIA Program Officer or designee will be invited to attend all meetings.

DSMB meetings will consist of open and closed sessions. Open session discussion will focus on the conduct and progress of the study, including participant accrual, protocol compliance, and problems encountered. The closed sessions will be attended by the DSMB members and the study statistician. Meetings shall be closed to the public because discussions may address confidential participant data. During the closed sessions, data will be presented and discussed. An executive, open session may follow a closed session at request of PI or DSMB; at that time, the Board will inform the NIA Program Officer and PI of their recommendation to continue or to terminate the study as was decided by the formal DSMB vote in closed session. The PI and key members of the study team will generally attend the open and executive sessions of the meetings.

At their first meeting, the DSMB will discuss the protocol and establish guidelines for study monitoring. The DSMB Chairperson, in consultation with the PI and the NIA Program Officer as needed, will prepare the agenda. Unless otherwise designated by the DSMB, the DSMB Chair will serve as the contact person for unanticipated problem and serious adverse event reporting. Procedures for notifying the Chair of the DSMB and the NIA Program Official of any such events will also be discussed. The format and content of the reports for both the open and closed sessions will be finalized and approved, although additional changes and requests can be addressed throughout the study. At the first meeting, the Board will decide if an interim analysis will take place (e.g. after n participants have been accrued or completed the trial). Interim analyses of efficacy data will only be performed if planned and approved in advance, and when criteria for possible stopping are clearly defined. Well-defined stopping rules will be established during the first DSMB meeting to guide expected causes of termination.

The PI and Dr. Morgan (study statistician) will be responsible for coordinating activities of the DSMB including the following: 1) Arranging DSMB meetings and communications. 2) Identifying and reviewing open session materials to be presented to the DSMB. DSMB meeting materials to be reviewed by the DSMB members will be prepared by Dr. Morgan and sent to members at least 7 days before the meeting. Interim data reports will consist of two parts: Part 1 –Open Session Reports (for example, accrual, participant baseline characteristics, adverse events, serious adverse events); and Part 2 - Closed Session Reports.

The Chair of the DSMB will be responsible for the following: 1) developing the meeting agenda; 2) requesting information for the meetings from the PI and the study statistician; 3) overseeing the meetings; 4) verifying that the reports and recommendations prepared are an accurate and complete record of the DSMB's deliberations; 5) serving as contact person for unanticipated problem and serious adverse event reporting.

The DSMB members will 1) familiarize themselves with the research protocol and consent forms, 2) review interim reports of adverse events, 3) review interim analyses of outcome data as it relates to safety, 4) review interim reports of trial participant accrual, and 5) make recommendations to the investigators concerning continuation,

termination, or modification of the trial.

Between meetings of the DSMB and throughout the course of the trial, information regarding issues deemed critical to the trial or to the safety of research participants will be provided to the Chair of the DSMB and the Program Officer by the PI.

A formal report containing the recommendations for continuation or modifications of the study will be prepared by the DSMB Chairperson. This draft report will be sent to the DSMB members within 7 days after the meeting. Once approved by the DSMB members, and no later than 2 weeks after the DSMB meeting, the DSMB Chair will forward the formal DSMB recommendation to the NIA Program Officer and PI. It is the responsibility of the PI to distribute the DSMB recommendation to all co-investigators and to ensure that copies are submitted to all the IRBs associated with the study when necessary.

If study termination is ever considered by the DSMB, the full vote of the DSMB will be required. A recommendation to terminate the study will be made by a majority vote. The DSMB Chair will provide the tiebreaking vote in the event of a 50-50 split vote. In the event of a majority vote for continuation with at least one vote for study termination, a written statement with accompanying reasons for the vote(s) to terminate should be contained within the report issued by the DSMB. In the event of a recommendation to modify the protocol or to terminate the study, the PI may disagree with the recommendation of the DSMB. If the PI does not concur with the recommendation of the DSMB, then the IRB, the institution official, and the funding agency must be notified in writing of this disagreement and the reason for it. The PI may appeal the decision and offer an alternative to termination or study modification for consideration by the DSMB. If an impasse is reached, the appeal will be forwarded to the Chair of the Institutional IRB who will review the case under advisement from the PI and the Chair of the DSMB and will recommend a course of action. This course of action will be recorded by the DSMB and the NIA Program Officer.

If no, please justify why not.

Is there a Data Safety Monitoring Plan (DSMP)? Y

If yes, describe the data and safety monitoring plan developed to ensure the safety of participants and the validity and integrity of research data. Monitoring should be commensurate with risks and with the size and complexity of the trials. As such, state that SAEs will be reviewed by a qualified MD in real time and indicate how often aggregate data will be reviewed for safety trends.

Data and Safety Monitoring Plan

1.0 Participant Safety

1.1 Potential Risks and Benefits for Participants

Potential Risks
The risks for participants in this trial are minimal. There are no anticipated physical or economic risks associated with this study. Regarding psychological risks to NH residents, the LOCK sleep program including frontline huddling provides a sequence of steps for the process of assessing and responding to sleep problems. During the intervention period NH residents enrolled in the LOCK sleep program will receive actigraph and Fitbit assessment, and although our team's experience with use of these devices in hundreds of people with dementia indicates that they are widely tolerated as comfortable, there is a small risk a resident may find the devices to be uncomfortable or otherwise distressing. As part of their intervention training, staff will be instructed on

behavioral signs of distress. If a participant exhibits significant behavioral distress due to assessment, staff will be instructed on modifications and alternatives. If the distress does not subside, staff will be instructed to discontinue the assessment (staff training also discussed in 1.3.b. Protection Against Risks).

For both NH residents and staff, an unlikely legal or social risk associated with participating in this study would be invasion of privacy in the remote chance that there is a loss or lack of protection of data containing personally identifiable information. These are research risks, not therapeutic risks. The study team will use careful procedures to guard the security and confidentiality of all data (see 1.3.b. Protection Against Risks).

For both NH residents and staff, a potential risk is that a person may not want to participate in the study and may feel coerced to do so. Therefore, to minimize this type of risk, all informed consent contact with potential participants will come through the study staff only, and all NH staff participation discussions and decisions will be kept confidential (see 1.3.b. Protection Against Risks).

Potential Benefits
The intervention may benefit NH residents by leading to better and earlier detection and treatment of sleep problems and from increased attention to their conditions. The intervention may benefit NH staff by improving communication and teamwork, thereby improving the work experience.

1.2 Adverse Event/Serious Adverse Event/Unanticipated Problem Collection and Reporting
Definitions
We will follow OHRP and UA IRB guidance and policies regarding definitions, collection, and reporting of Adverse Events (AEs), Serious Adverse Events (SAEs), and Unanticipated Problems (UPs). We expect that there will potentially be four categories of events/problems as follows: 1) Anticipated AEs unrelated to the intervention, 2) Anticipated AEs related to the intervention, 3) Unanticipated AEs and unanticipated problems unrelated to the intervention, and 4) Unanticipated AEs and unanticipated problems related to the intervention.

Reporting
As per National Institute on Aging (NIA) policy, all deaths will be reported within 24 hours of the study staff's knowledge of death to the NIA Program Officer and the DSMB Chair. Unanticipated SAEs related to the intervention will be reported within 48 hours of the study staff's knowledge of death to the NIA Program Officer and the DSMB Chair. A summary of all other SAEs will be reported quarterly to the NIA Program Officer and the DSMB Chair.

Study staff will continue to follow up with the participant and their treating clinical team until any clinical events have resolved. If there is a SAE that is thought by a treating clinician, co-investigator nurse scientist Dr. Richards, study consultant geriatrician Dr. Fry, or Dr. Snow to be possibly or probably related to the intervention, the PI will immediately notify the DSMB and NIA Program Officer. The PI or designee will also immediately communicate with the NH leadership team and corporate coach at the NH for immediate investigation by the relevant treating team. Any insights about any possible iatrogenic effects of any components of the intervention will be immediately shared with all active NH intervention sites.

Grading Scale and Study Relatedness Criteria

Anticipated AEs unrelated to the intervention: NH residents enrolled in the trial will have dementia and likely will have other pre-existing significant health issues that require NH

placement. It is anticipated that some participants will experience health events (new conditions or worsening of previous conditions), hospitalization, or death during the trial that will be due to the dementia and/or other pre-existing significant health issues and unrelated to the intervention. As per NIA policy, all deaths will be reported to the NIA and IRB within 24 hours, regardless of relation to intervention.

Anticipated AEs related to the intervention: Intervention-related AEs for NH residents might include: a) actigraph/fitbit-related skin reactions such as redness, itching, or other skin conditions or discomfort; b) distress or discomfort associated with actigraph/fitbit-related wear or NH staff attention; c) distress or discomfort associated with other LOCK sleep intervention NH staff attention or requests. Intervention-related AEs for NH staff might include: distress related to interview questions. Such events may be reportable depending upon the seriousness of the event and increase of risk to participant. The NH's treating clinical team's opinion of the seriousness of the event will be solicited and the PI, co-investigator nurse scientist Dr. Richards and study co-investigator geriatrician Dr. Fry will be consulted to determine seriousness and reportability of the event.

Unanticipated AEs and unanticipated problems related to the intervention: As with intervention-related AEs, unanticipated AEs and unanticipated problems will be evaluated for seriousness with input from the NH's treating clinical team (if applicable), the PI and clinical study team members Drs. Richards and Fry (if applicable) or data-related study team members including data coordinating center leader and study statistician Dr. Morgan (if applicable).

1.3 Protection Against Study Risks

a. Informed Consent and Assent
Study staff will obtain all necessary IRB authorizations for protocols and materials described below. All consents will be conducted by an IRB-approved study staff member who has been trained by Dr. Snow. The study staff member will be responsible for explaining the study, answering questions, and obtaining informed consent.

NH Resident Consent – Step 1. Legally Authorized Representative Consent
NH leadership team or their designees will identify all NH residents with an ADRD diagnosis and mail to their legally authorized representatives (LARs) an IRB-approved study introduction letter (including option to opt out within one month if they do not wish to be contacted by study staff). Opt-out procedures have been demonstrated to yield higher response rates and lower rates of non-response bias compared to opt-in procedures, and to be acceptable to participants, with no difference in rates of reported distress or complaints compared to opt-in procedures.105-108 In our past federally funded research involving people with dementia, opt-out consent has been approved by multiple IRBs and worked well. Contact information of those LARs not opting out will be shared with University of Alabama (UA) study staff. We will request of the IRB a waiver of consent and HIPAA authorization for screening purposes regarding the sharing of these contact information.

Study staff will mail an IRB-approved study information packet via a tracked, express mailing service to all LARs who do not opt out. These materials will include a cover letter, an IRB-approved informed consent form (ICF), and an express mail return envelope so the LAR can send the signed ICF to the UA study office. LARs will be encouraged to contact study staff via phone or email for an appointment to discuss the study and go through the consent form together before signing the ICFs. Study staff will contact the LARs via phone and/or email within one week of sending the packet and will re-contact the LAR up to 6 times within the next month as needed to establish contact (if contact has not been established within one month, and NH staff are not able to determine that the LAR's contact information has changed, then the lack of contact will

be considered a soft refusal and no other contact will be attempted).

During a phone appointment, study staff will review all important elements of the ICF with the LAR, including the following: description of the study, information on how to contact the investigators and the NH administrator and director of nursing, a statement that there is no prejudice for refusal to participate, a statement that the subject may withdraw from the study at any time without prejudice, and statement regarding risk and benefit of participation. The LAR will be encouraged to ask questions. The LAR will be encouraged to call the PI if they have any remaining questions before signing the consent form.

NH Resident Consent – Step 2. Screening for Capacity for NH Resident Consent/NH Resident Assent
Some NH residents with mild ADRD may have the capacity to provide their own consent. A two-step process will be used to address the ethical balance of the beneficence of assuring that residents who do not have the capacity to consent are protected by LAR oversight with the promotion of autonomy for residents who do have capacity to consent. Once LAR consent is received for a resident, the NH leadership team will be asked to identify that resident's most recent MDS Brief Interview for Mental Status120 (BIMS) score, a cognitive screening measure with good reliability and validity. For residents with BIMS scores in the mild range (above 10), the NH leadership team will be asked to arrange and support a telephone appointment between that resident and a study staff person (if the resident turns out to not have the cognitive ability to participate in the following telephone-based procedure, then they would also not have the cognitive capacity to provide informed consent, which requires higher level cognitive abilities).

In the telephone appointment, the study staff member will provide information about the study and will screen for capacity to consent by assessing the following. Does the resident respond appropriately to a social greeting? Is the resident oriented to place? Does the resident understand who the study team member is after the team member explains their identity? During the course of this introductory conversation, was the resident able to respond fluently and socially appropriately? If so, the team member will go through the consent form with them. Residents will then be asked a series of questions to assure their understanding. These questions are as follows:
Describe in your own words what this study is about?
Describe what I am asking you to do to participate in this study?
Are you required to do this interview with me?
What will happen if you don't talk to me?
What happens if you decide you don't want to participate once we get started?
What will I do with the information you give me?

The NH leadership team member will support this telephone conversation by assuring that the resident has a quiet environment to have the telephone conversation and that the resident has in front of them a printed large-font version of the ICF with the signature area flagged. If the resident is able to respond appropriately to all the capacity screening questions, and they do wish to participate, then the resident will be instructed to sign the ICF. The study staff person will contact the NH leadership team member and ask them to make a copy of the ICF for the resident to keep, place a copy in the medical chart, and express mail the original to the UA study office using a provided pre-paid envelope. If the resident is able to respond appropriately to all the capacity screening questions and does not wish to participate then they will not be enrolled in the study, regardless of the LAR's consent. In this case, the study team member will let the NH leadership team and the LAR know that the resident has declined participation.

Under all circumstances, the NH resident's autonomy will be respected. For NH

residents without capacity to consent, their assent to participate will be obtained whenever possible, and their decision to withdraw at any time (whether expressed verbally or by resistance to participation) will be honored (see also staff training in 1.3.b. Protection Against Risks).

All above procedures apply to securing consent for the study team to have access to NH resident personally identifiable data. We note that because the LOCK sleep program does not include any activities that are outside of standard NH practice, NH staff will be free to enroll any NH residents that they believe will benefit into the LOCK sleep program regardless of resident consent status; but the NH leadership team will only share with researchers data from residents who are consented for the study.

NH Staff Consent
The NH leadership team or their designees will provide to all NH staff an IRB-approved study introduction letter (including option to opt out within two weeks if they do not wish to be contacted by study staff). Contact information of those NH staff not opting out will be shared with UA study staff. We will request of the IRB a waiver of consent for screening purposes regarding the sharing of these contact information.

UA Study staff will contact NH staff (via email and/or phone, see next paragraph) to invite them to participate in mid-implementation phone interviews. Study staff will also contact NH staff to invite them to participate in post-implementation interviews (phone and in-person). The list of NH staff who agree and decline interview participation will remain confidential to protect NH staff from any possible coercion to participate (see 1.3.b. Protection Against Risks).

For mid-implementation interviews, study team members will, on the PI’s behalf, send out e-mails to all NH staff who do not opt out. The first e-mail will contain text from Dr. Snow soliciting voluntary participation in the telephone interviews. E-mail reminders will be sent, one in each of the four weeks after the initial e-mail, with follow up phone calls to the participant’s work phone to remind them about the email. All emails will include information on how to opt out of the study. We will continue recruitment until we have recruited the desired sample size or until one month has passed. To facilitate staff involvement, we will secure permission from the director of each NH that staff may take part in the study during their normal hours of work. For post-implementation interviews, study team members will follow these same procedures. In addition, a study team member will visit each NH in person at the end of the intervention period to collect information from the enrolled NH residents’ medical record and also to recruit for and conduct any needed remaining post-implementation interviews in person. The study staff person will attend change of shift meetings, huddles, and other staff meetings to invite interview participation and will also approach staff one-on-one to discuss interviews and to ask if the staff member would like to volunteer to participate. We have successfully used these procedures in our previous federally funded studies involving NH staff.

In all recruitment activities, it will be made clear that participation is strictly voluntary and no adverse consequences to the person’s job status or any other adverse consequences will occur if the person declines to participate in these activities and that their final decision to participate or not will be kept confidential from all NH and corporate staff.

Mid-implementation interviews will take place via telephone. Post-implementation interviews will take place either via telephone or in person at a NH location offering privacy.

We will apply for a waiver of documentation of informed consent for the 1-hour, audio-

recorded mid-implementation and post-implementation interviews, allowing us to obtain verbal consent. We will provide a consent information sheet to potential participants in the stead of an informed consent document that requires signature. We will record each subject giving permission to be recorded on the audio recording.

The study staff member will review with the NH staff member all relevant elements of consent on the consent information sheet, including the following: description of the study activities, time requirement, information on how to contact the investigators, a statement that there is no prejudice for refusal to participate, a statement that the subject may withdraw from the study at any time without prejudice, a statement encouraging the reader to ask questions, and a statement regarding risk and benefit of participation. All informed consent guidelines of the IRB will be followed. If at any time during the interview the staff member indicates a desire to stop participating, the participant will be withdrawn from the interview portion of the study. Confidentiality of all interview data will be protected by using participant code numbers on the data collection forms.

b. Protection Against Risks
Study staff training
To protect all vulnerable subjects, in addition to the NIH-required computer-based trainings (on the protection of human research participants, HIPAA, and Good Clinical Practice), the UA study staff will attend an in-person 2-day training session specific to the proposed project led by Dr. Snow. The PI will review the overall goals of the study, study policies and procedures, data collection manuals, adverse event identification and reporting, subject confidentiality, communication techniques and principles for working with persons with dementia, and appropriate procedures for working by telephone with LARs for consent, for working by telephone with people with dementia for consent and for determining capacity to consent. Procedures will be reviewed for protecting employees from possible coercion for participation from supervisors including maintaining confidentiality of those employees who do and do not consent to research interviews. During these training sessions, professionally produced videotapes of staff interactions with persons with dementia will be shown and the PI and study staff will role play all informed consent, capacity assessment, and data collection processes. Dr. Richards (via skype) and Parmelee (in person) will work together to provide training on appropriate assessment equipment maintenance and data downloading. Drs. McCullough and Hartmann (via skype) will provide training on appropriate procedures for NH staff interviews.

NH leadership team training: The NH leadership team will complete NIH-required computer-based trainings (on the protection of human research participants, HIPAA, and Good Clinical Principles). Study staff will also provide a 2-hour training on procedures to protect vulnerable subjects, as follows: proper procedure for, and importance of only using IRB-approved procedures and letters when sending study opt-out letters to LARs; proper procedures for maintaining security and privacy of data packages mailed to the University of Alabama project office; proper procedures for avoiding coercion or appearance of coercion of research participation when working with NH staff (e.g., not asking staff about research interview participation); proper procedures for assisting in telephone appointment arrangements and providing logistical support when study team members conduct telephone-based NH resident consenting and capacity to consent assessment; importance of only study team members conducting actual consent procedures of anyone (LARs, NH residents, NH staff); importance of only properly trained NH leadership team members participating in study procedures. Dr. Richards and Parmelee (via skype teleconference) will work together to provide training on the appropriate application of actigraphs and Fitbits to promote comfort and prevent adverse experiences, as well as appropriate assessment equipment maintenance and data mailing.

NH staff training: As part of the intervention training, NH staff will be trained by their NH leadership teams or their trained designees on proper use and placement of actigraphs and Fitbits, verbal and behavioral signs of distress that might indicate actigraph/Fitbit-related discomfort/distress, and appropriate modification techniques to try to relieve such distress. NH staff will be informed that the devices should be removed in the unlikely event that distress behaviors are not relieved by modification techniques. NH staff will be trained on importance of NH resident autonomy, and will be engaged in a discussion of how to assess for and honor NH resident assent or lack thereof with regard to the devices.

Personally Identifiable Information: Access, Management, and Protection
The study staff will have access to individually identifiable data about participants. The NH leadership team will have access to individually identifiable data about participants (see also Protection of Human Subjects, section 1.a, sub-sections Subject Populations and Collaborating Sites and Roles). To protect against the risk of accidental access to or inappropriate divulging of identifiable data, the flow of individually identifiable data and steps to assure its appropriate access, management, and protection, are outlined below.

1. NH Residents
a. Study staff will successfully obtain all necessary IRB authorizations for protocols and materials described below

b. NH leadership team or their designees will identify all NH residents with an ADRD diagnosis and the NH leadership team will mail to those residents' legally authorized representatives (LARs) an IRB-approved study introduction letter (including option to opt out within one month if they do not wish to be contacted by study staff). Contact information of those LARs not opting out will be shared with University of Alabama (UA) study staff.

c. UA study staff will contact LARs and attempt to gain informed consent for NH resident participation. Study staff will weekly provide an updated list of consented residents with study identification numbers to the NH leadership team along with copies of all completed informed consent forms so that a copy can be filed in the resident's medical records and a copy can be stored along with the complete list and other IRB approvals and relevant study information in an essential documents binder at the NH. These processes will assure that NH staff will be able to easily verify that study staff only have access to personally identifiable data of residents are involved in the study.

d. NH staff will work together in frontline huddles to complete screening of all eligible residents, identify residents with sleep problems to enroll in the LOCK sleep program, and collect assessment data for enrolled residents.

e. Every week, the NH leadership team will use researcher-provided tracked express mail service envelopes to send the UA study staff the following: 1) all completed staff rating forms, identified by NH resident subject number; and 2) any actigraphs and Fitbits for which the assessment periods are complete, identified by subject number. Research staff will mail back actigraphs and Fitbits after data download, deletion of data from the devices, and maintenance.

f. UA study staff will travel to each NH once during the post-intervention period to extract information from the medical records of consented NH residents (this process and the information to be extracted will be clearly described in IRB-approved protocols and consent forms).

g. UA study staff will upload all data at UA and then transfer to the University of Texas School of Public Health (UTHealth SPH) Data Center via secure file transfer procedures as established by Dr. Morgan and the Data Center.

h. MDS data will be obtained by Dr. Morgan and the UTHealth SPH Data Center through data use agreement for use of personally identifiable information from CMS. MDS data for consented NH residents will be linked and merged with the other data described above.

i. When data are reported in publication and presentation they will only be reported in aggregate form, and neither NH nor participant will be identifiable.

2. NH Staff for interviews
a. Study staff will successfully obtain all necessary IRB authorizations for protocols and materials described below.

b. NH leadership team will provide to all NH staff an IRB-approved study introduction letter (including option to opt out within one month if they do not wish to be contacted by study staff). Contact information of those NH staff not opting out will be shared with UA study staff.

c. UA Study staff will contact NH staff to invite them to participate in mid-implementation phone interviews. Study staff will also contact NH staff to invite them to participate in post-implementation interviews. The list of NH staff who agree and decline interview participation will remain confidential to protect NH staff from any possible coercion to participate (see 1.3.b. Protection Against Risks).

d. UA study staff will conduct interviews with consented NH staff. Interviews will be audio recorded. Recordings will be uploaded at UA, identified only by subject number, and shared with a university-approved transcription service via secure file transfer procedures as established by Dr. Morgan and the UTHealth SPH Data Center. Transcribed files, identified only by subject number, will be returned via the same secure file transfer procedures to UA for tracked storage and analysis and to Drs. McCullough and Hartmann at Boston University for analysis.

e. When data are reported in publication and presentation they will only be reported in aggregate form, and neither NH nor participant will be identifiable.

3. Data Management and Protection. Dr. Morgan and the UTHealth SPH Data Center will create protocols for overseeing the security and protection of all data collected for this study. Personally identifiable information will be only be stored in the secure and securely backed up UA data server and UTHealth SPH Data Center in password protected files. Other collaborating sites (Boston University, Brown University, and University of Texas at Austin) will only work with data identified by subject number.

2.0 Interim Analysis
At the first meeting, the Data Safety and Monitoring Board (DSMB) will decide if an interim analysis will take place (e.g. after n participants have been accrued or completed the trial). Interim analyses of efficacy data will only be performed if planned and approved in advance, and when criteria for possible stopping are clearly defined.

It is not anticipated that interim analyses will be undertaken given the minimal potential risks to participants in this trial.

3.0 Data and Safety Monitoring
The DSMB procedures are described above in the Risks Sub-section #8b.

If no, please justify why not.

*** * * Benefits/Alternatives, Procedures to Maintain Confidentiality * * ***

9. Benefits/Alternatives

a) **Benefits. Describe the potential benefit(s) to be gained by the participants and how the results of the study may benefit future participants and/or society in general. Indicate if there is no direct benefit to the participants.**

The intervention may benefit NH residents by leading to better and earlier detection and treatment of sleep problems and from increased attention to their conditions. The intervention may benefit NH staff by improving communication and teamwork, thereby improving the work experience.

Sleep problems and related issues in NH residents with dementia is a well-documented problem. This study implements an intervention to improve the sleep experience for these residents. If the LOCK sleep program is found to be effective in enhancing clinical care and outcomes, the approach can be shared with other NHs and incorporated into general NH practice. The risks in this study for NH residents are minimal, and the knowledge gained may result in improved quality of sleep, and ultimately improved quality of life for NH residents with dementia. Similarly the risks for NH staff are minimal, and the knowledge to be gained may result in a more effective and enjoyable workplace experience. Thus, the potential benefits exceed risks.

b) **Alternatives. Describe any alternative treatments and procedures available to the participants should they choose not to participate in the study. If no such alternatives exist, please state that the alternative is nonparticipation. For some studies, such as record reviews, a description of alternatives would not be applicable.**

The intervention described will be implemented NH-wide. The study uses an incomplete stepped-wedge cluster randomized controlled trial design in which each NH serves as its own control. There will therefore be no alternative treatments. NH residents and their legally authorized representatives will have the right to refuse participation in any portion of their care just as they would with usual NH care, including use of actigraphs and Fitbits. NH staff will have the right to refuse any interviews.

10. Procedures to Maintain Confidentiality

Federal regulations require that study data and consent documents be kept for a minimum of three (3) years, and HIPAA documents be kept for a minimum of six (6) years after the completion of the study by the PI. For longitudinal or sponsored projects, the PI may be required to keep the data and documents for a longer time period.

Data Security

Please indicate how information will be secured. All information must be stored using at least two of the following safeguards and must be kept in accordance with the University of Alabama Information Security Policies. (If you are using both electronic data and hard copy data, you will need two safeguards for each type).

a) Electronic Data: (mark all that apply - at least 2 - or indicate not applicable)

- Not applicable
- X Password access
- X Coded, with a master list kept as a hardcopy or on a secure network (confidential)
- Data collected anonymously
- Secure network (e.g., firewall)
- Data are de-identified by PI or research team
- Other

Please specify:

b) Hardcopy Data: (mark all that apply - at least 2 - or indicate not applicable))

- Not applicable
- Locked suite
- X Locked office
- X Locked file cabinet
- X Coded, with a master list secured and kept separately (confidential)
- Data collected anonymously
- 24 hour personnel supervision
- Data are de-identified by PI or research team
- Other

Please specify:

c) Describe measures employed to protect the identity of the participants, their responses, and any data that you obtain from private records (e.g., identifiers will be stripped so data cannot be linked to participants, or code numbers will be used, etc.). If data will be coded, specify the procedures for coding the data so that confidentiality of individual participants is protected. If you will keep a master list linking study codes to participant identifiers, explain why this is necessary, how and where you will secure the master list, and how long it will be kept.

Sources of Materials
NH resident data will be collected for consented NH residents from NH staff ratings (global assessments of change in overall sleep quality and sleep-related conditions) and actigraph/Fitbit measurements (wristwatch-sized devices worn on the wrist); medical records (age, diagnoses, medical history, and medications); and MDS assessments (Brief Interview for Mental Status score, psychotropic medication use, pain and analgesic medication use, activities of daily living decline, sleep-related items). NH staff interview data will be collected via audiorecorded qualitative interview. See Table 1 in Attachments for a listing of all study outcomes and data sources.

Personally Identifiable Information: Access, Management, and Protection
The study staff will have access to individually identifiable data about participants. The NH leadership team will have access to individually identifiable data about participants. The flow of individually identifiable data and steps to assure its appropriate access, management, and protection, are outlined below.

1. NH Residents
a. Study staff will successfully obtain all necessary IRB authorizations for protocols and materials described below

b. NH leadership team or their designees will identify all NH residents with an ADRD diagnosis and the NH leadership team will mail to those residents' legally authorized representatives (LARs) an IRB-approved study introduction letter (including option to opt out within one month if they do not wish to be contacted by study staff). Contact information of those LARs not opting out will be shared with University of Alabama (UA) study staff.

c. UA study staff will contact LARs and attempt to gain informed consent for NH resident participation (see 2.a. Informed Consent and Assent). Study staff will weekly provide an updated list of consented residents with study identification numbers to the NH

leadership team along with copies of all completed informed consent forms so that a copy can be filed in the resident's medical records and a copy can be stored along with the complete list and other IRB approvals and relevant study information in an essential documents binder at the NH. These processes will assure that NH staff will be able to easily verify that study staff only have access to personally identifiable data of residents are involved in the study.

d. NH staff will work together in frontline huddles to complete screening of all eligible residents, identify residents with sleep problems to enroll in the LOCK sleep program, and collect assessment data for enrolled residents.

e. Every week, the NH leadership team will use researcher-provided tracked express mail service envelopes to send the UA study staff the following: 1) all completed staff rating forms, identified by NH resident subject number; and 2) any actigraphs and Fitbits for which the assessment periods are complete, identified by subject number. Research staff will mail back actigraphs and Fitbits after data download, deletion of data from the devices, and maintenance.

f. UA study staff will travel to each NH once during the post-intervention period to extract information from the medical records of consented NH residents (this process and the information to be extracted will be clearly described in IRB-approved protocols and consent forms).

g. UA study staff will upload all data at UA and then transfer to the University of Texas School of Public Health (UTHealth SPH) Data Center via secure file transfer procedures as established by Dr. Morgan and the Data Center.

h. MDS data will be obtained by Dr. Morgan and the UTHealth SPH Data Center through data use agreement for use of personally identifiable information from CMS. MDS data for consented NH residents will be linked and merged with the other data described above.

i. When data are reported in publication and presentation they will only be reported in aggregate form, and neither NH nor participant will be identifiable.

2. NH Staff for interviews
a. Study staff will successfully obtain all necessary IRB authorizations for protocols and materials described below.

b. NH leadership team will provide to all NH staff an IRB-approved study introduction letter (including option to opt out within one month if they do not wish to be contacted by study staff). Contact information of those NH staff not opting out will be shared with UA study staff.

c. UA Study staff will contact NH staff to invite them to participate in mid-implementation phone interviews. Study staff will also contact NH staff to invite them to participate in post-implementation interviews. The list of NH staff who agree and decline interview participation will remain confidential to protect NH staff from any possible coercion to participate.

d. UA study staff will conduct interviews with consented NH staff. Interviews will be audio recorded. Recordings will be uploaded at UA, identified only by subject number, and shared with a university-approved transcription service via secure file transfer procedures as established by Dr. Morgan and the UTHealth SPH Data Center. Transcribed files, identified only by subject number, will be returned via the same secure file transfer procedures to UA for tracked storage and analysis and to Dr. Hartmann at

UMass Lowell for analysis.

e. When data are reported in publication and presentation they will only be reported in aggregate form, and neither NH nor participant will be identifiable.

3. Data Management and Protection. Dr. Morgan and the UTHealth SPH Data Center will create protocols for overseeing the security and protection of all data collected for this study. Personally identifiable information will be only be stored in the secure and securely backed up UA data server and UTHealth SPH Data Center in password protected files. Other collaborating sites (UMass Lowell, Brown University, and University of Texas at Austin) will only work with data identified by subject number.

d) If data or specimens are being shared outside of the research team, indicate who will receive the material and specifically what they will receive (data or specimens).

N/A. Data will be shared across different sites that are all part of the research team -- this is described above.

e) If samples or data will be provided from an outside source, indicate whether you will have access to identifiers, and, if so, how identifiable information is protected. Please provide a letter from the appropriate persons indicating that data will be provided in a de-identified manner.

Data will be collected from NH medical records and from NH Minimum Data Set (MDS) records. We will have access to identifiers, and are submitting the appropriate HIPPAA authorizations. We will protect data by separating identifiers from the data itself using subject IDs and a master coding checklist kept locked in a separate location.

f) If data will be collected via e-mail or the internet, how will anonymity or confidentiality be protected? Describe how data will be protected during electronic transmission and how data will be recorded (i.e., will internet protocol (IP) address and/or e-mail addresses be removed from data?).

g) If you will be audio/video recording or photographing participants, provide a rationale for recording/photographing. Describe confidentiality procedures, including the final disposition of the recordings/photos (destruction, archiving, etc.) and a reasonable timeline by which this disposition will occur.

----

* * * Potential Conflict of Interest * * *

11) Potential Conflict of Interest

Federal regulations and UA policy require all investigators to disclose their significant financial interests to allow a review of potential conflicts of interest. If a potential conflict of interest is identified, a formal plan must be developed and implemented to manage, reduce, or eliminate the conflict.

Examples of significant financial interests include receipt of income, honoraria, and stock or stock options from a public or private entity sponsoring the research. They may also include a consulting arrangement or membership on an advisory board of the entity. Significant financial interests are reported on the UA Statement of Financial Interest.

All members of the research team who are involved in the design, conduct, or reporting of research (i.e., senior/key personnel) should have a current Statement of Financial Interest and conflict of interest training on file prior to submitting the IRB protocol. Please refer to the Office for Research Compliance website for additional information regarding the financial conflict of interest requirements, as well as links to the disclosure form and training at (http://osp.ua.edu/site/RC_CoI.html).

The Statement of Financial Interest must be submitted annually and within 30 days of discovering or acquiring a new or increased financial interest. Conflict of interest training must be completed once every four years.

If such a relationship as described above exists between a member of the research team and the sponsor of the research, the investigator is also required to disclose this relationship and identify the entity involved on the informed consent form. For questions regarding Conflict of Interest consult the   Conflict of Interest in Research Policy.

Check one of the following:

1) X No Financial Interest or Financial interest less than or equal to $5K
2) Financial Interest exceeding $5K but not exceeding $25K, and/or more than 5 percent equity interest in aggregate
3) Financial Interest exceeding $25K

Check all those that apply:

Consulting

Speaking Fees or Honoraria

Gifts

Patent

Copyright

Licensing agreement or royalty income

Equity interests, (including stock, stock options, warrants, partnership or equitable ownership interests), or serving on a scientific advisory board or board of directors

Other fees/compensation

Describe financial interests(s) and indicate specific amounts for each subcategory checked. Be sure to describe how these financial interests relate to the protocol being submitted.

Note to Investigator(s) Reporting a Potential Conflict of Interest

Investigator(s) must have:

1) Current, up-to-date Conflict of Interest Disclosure Form on file with the University of Alabama Conflict of Interest Committee (COIC) that describes any financial relationship indicated above.

. This information must be disclosed on the University of Alabama confidential Conflict of Interest Disclosure Form for review by the COIC before accruing research participants in this study. If your current Disclosure Form does not contain this information, you are required to submit an updated Disclosure Form to the COIC.

2) Financial disclosure statement incorporated into the consent document. Please see Model Consent for suggested language.

3) You may not begin your study until your disclosure form has been reviewed and any required management plan has been approved by the COIC.

Does any member of the study team, members' spouses, or members' dependent children have any significant financial interests related to the work to be conducted as part of the above-referenced project? N

Name of Personnel with Financial Conflict of Interest

Other research staff that may have a conflict. Please specify below.

Any member of the study team who answers in the affirmative must be listed in the box below.

A staff person will contact any researcher listed above to obtain additional information regarding the specific financial interest(s).

I certify that all members of the study team have answered the financial interests question and only those individuals listed in the box above have disclosed any financial interest related to this study. Y

----

* * * Informed Consent * * *

12 Informed Consent

Federal regulations require that informed consent be obtained from individuals prior to their participation in research unless the IRB grants a waiver of consent. Answer the questions, below, then click Add to provide the necessary consent documents and information regarding participant consent. Multiple consents/waivers may be added, but they must be uploaded one at a time.

NOTE: You may refer to the University of Alabama IRB Guidance for Obtaining Informed Consent for considerations regarding the consent/assent process.

State N/A if not applicable.

1) How is consent being obtained? When and where will the discussion take place?

Informed Consent and Assent
Study staff will obtain all necessary IRB authorizations for protocols and materials described below. All consents will be conducted by an IRB-approved study staff member who has been trained by Dr. Snow. The study staff member will be responsible for explaining the study, answering questions, and obtaining informed consent.

NH RESIDENT CONSENT – Step 1. Legally Authorized Representative Consent
NH leadership team or their designees will identify all NH residents with an ADRD diagnosis and mail to their legally authorized representatives (LARs) an IRB-approved study introduction letter (including option to opt out within one month if they do not wish to be contacted by study staff). Opt-out procedures have been demonstrated to yield higher response rates and lower rates of non-response bias compared to opt-in procedures, and to be acceptable to participants, with no difference in rates of reported distress or complaints compared to opt-in procedures.105-108 In our past federally funded research involving people with dementia, opt-out consent has been approved by multiple IRBs and worked well. Contact information of those LARs not opting out will be shared with University of Alabama (UA) study staff. We will request of the IRB a waiver of consent and HIPAA authorization for screening purposes regarding the sharing of these contact information.
Study staff will mail an IRB-approved study information packet via a tracked, express mailing service to all LARs who do not opt out. These materials will include a cover letter, an IRB-approved informed consent form (ICF), and an express mail return envelope so the LAR can send the signed ICF to the UA study office. LARs will be encouraged to contact study staff via phone or email for an appointment to discuss the study and go through the consent form together before signing the ICFs. Study staff will contact the LARs via phone and/or email within one week of sending the packet and will re-contact the LAR up to 8 times within the next month as needed to establish contact (if contact has not been established within one month, and NH staff are not able to determine that the LAR's contact information has changed, then the lack of contact will be considered a soft refusal and no other contact will be attempted).

During a phone appointment, study staff will review all important elements of the ICF with the LAR, including the following: description of the study, information on how to contact the investigators and the NH administrator and director of nursing, a statement that there is no prejudice for refusal to participate, a statement that the subject may withdraw from the study at any time without prejudice, and statement regarding risk and benefit of participation. The LAR will be encouraged to ask questions. The LAR will be encouraged to call the PI if they have any remaining questions before signing the consent form.

NH RESIDENT CONSENT – Step 2. Screening for Capacity for NH Resident Consent/NH Resident Assent

Some NH residents with mild ADRD may have the capacity to provide their own consent. A two-step process will be used to address the ethical balance of the beneficence of assuring that residents who do not have the capacity to consent are protected by LAR oversight with the promotion of autonomy for residents who do have capacity to consent. Once LAR consent is received for a resident, the NH leadership team will be asked to identify that resident’s most recent MDS Brief Interview for Mental Status [120] (BIMS; see Appendix for copy of measure) score, a cognitive screening measure with good reliability and validity. For residents with BIMS scores in the mild range (above 10), the NH leadership team will be asked to arrange and support a telephone appointment between that resident and a study staff person (if the resident turns out to not have the cognitive ability to participate in the following telephone-based procedure, then they would also not have the cognitive capacity to provide informed consent, which requires higher level cognitive abilities).

In the telephone appointment, the study staff member will provide information about the study and will screen for capacity to consent by assessing the following. Does the resident respond appropriately to a social greeting? Is the resident oriented to place? Does the resident understand who the study team member is after the team member explains their identity? During the course of this introductory conversation, was the resident able to respond fluently and socially appropriately? If so, the team member will go through the consent form with them. Residents will then be asked a series of questions to assure their understanding. These questions are as follows:

- Describe in your own words what this study is about?
- Describe what I am asking you to do to participate in this study?
- Are you required to do this interview with me?
- What will happen if you don’t talk to me?
- What happens if you decide you don’t want to participate once we get started?
- What will I do with the information you give me?

The NH leadership team member will support this telephone conversation by assuring that the resident has a quiet environment to have the telephone conversation and that the resident has in front of them a printed large-font version of the ICF with the signature area flagged. If the resident is able to respond appropriately to all the capacity screening questions, and they do wish to participate, then the resident will be instructed to sign the ICF. The study staff person will contact the NH leadership team member and ask them to make a copy of the ICF for the resident to keep, place a copy in the medical chart, and express mail the original to the UA study office using a provided pre-paid envelope. If the resident is able to respond appropriately to all the capacity screening questions and does not wish to participate then they will not be enrolled in the study, regardless of the LAR’s consent. In this case, the study team member will let the NH leadership team and the LAR know that the resident has declined participation.

Under all circumstances, the NH resident’s autonomy will be respected. For NH residents without capacity to consent, their assent to participate will be obtained whenever possible, and their decision to withdraw at any time (whether expressed verbally or by resistance to participation) will be honored (see also staff training in 2.c Vulnerable Subjects).

All above procedures apply to securing consent for the study team to have access to NH resident personally identifiable data. We note that because the LOCK sleep program does not include any activities that are outside of standard NH practice, NH staff will be free to enroll any NH residents that they believe will benefit into the LOCK sleep program regardless of resident consent status; but the NH leadership team will only share with researchers data from residents who are consented for the study.

NH STAFF CONSENT

The NH leadership team or their designees will provide to all NH staff an IRB-approved study introduction letter (including option to opt out within two weeks if they do not wish to be contacted by study staff). Contact information of those NH staff not opting out will be shared with UA study staff. We will request of the IRB a waiver of consent for screening purposes regarding the sharing of these contact information.

UA Study staff will contact NH staff (via email and/or phone, see next paragraph) to invite them to participate in mid-implementation phone interviews. Study staff will also contact NH staff to invite them to participate in post-implementation interviews (phone and in-person). The list of NH staff who agree and decline interview participation will remain confidential to protect NH staff from any possible coercion to participate.

For mid-implementation interviews, study team members will, on the PI's behalf, send out e-mails to all NH staff who do not opt out. The first e-mail will contain text from Dr. Snow soliciting voluntary participation in the telephone interviews. E-mail reminders will be sent, one in each of the four weeks after the initial e-mail, with follow up phone calls to the participant's work phone to remind them about the email. All emails will include information on how to opt out of the study. We will continue recruitment until we have recruited the desired sample size or until one month has passed. To facilitate staff involvement, we will secure permission from the director of each NH that staff may take part in the study during their normal hours of work. For post-implementation interviews, study team members will follow these same procedures. In addition, a study team member will visit each NH in person at the end of the intervention period to collect information from the enrolled NH residents' medical record and also to recruit for and conduct any needed remaining post-implementation interviews in person. The study staff person will attend change of shift meetings, huddles, and other staff meetings to invite interview participation and will also approach staff one-on-one to discuss interviews and to ask if the staff member would like to volunteer to participate. We have successfully used these procedures in our previous federally funded studies involving NH staff.

In all recruitment activities, it will be made clear that participation is strictly voluntary and no adverse consequences to the person's job status or any other adverse consequences will occur if the person declines to participate in these activities and that their final decision to participate or not will be kept confidential from all NH and corporate staff.

Mid-implementation interviews will take place via telephone. Post-implementation interviews will take place either via telephone or in person at a NH location offering privacy.

We are applying for a waiver of documentation of informed consent for the 1-hour, audio-recorded mid-implementation, post-implementation, and sustainment interviews, allowing us to obtain verbal consent. We will provide a consent information sheet to potential participants in the stead of an informed consent document that requires signature. We will record each subject giving permission to be recorded on the audio recording.
The study staff member will review with the NH staff member all relevant elements of consent on the consent information sheet, including the following: description of the study activities, time requirement, information on how to contact the investigators, a statement that there is no prejudice for refusal to participate, a statement that the subject may withdraw from the study at any time without prejudice, a statement encouraging the reader to ask questions, and a statement regarding risk and benefit of participation. All informed consent guidelines of the IRB will be followed. If at any time during the interview the staff member indicates a desire to stop participating, the participant will be withdrawn from the interview portion of the study. Confidentiality of all interview data will be protected by using participant code numbers on the data collection forms.

SELECTED REFERENCES
120.Chodosh J, Edelen MO, Buchanan JL, et al. Nursing home assessment of cognitive impairment: development and testing of a brief instrument of mental status (BIMS). J Am Geriatr Soc. 2008;56(11):2069-2075.

2) **Explain how risks, benefits, and alternatives will be discussed.**

Risks will be described first. Efforts to mitigate risk (e.g., data security procedures to ensure confidentiality of data, procedures to keep employee participation information confidential, staff training to ensure that cognitively impaired individuals' autonomy and potential withdrawal of assent are honored).

Benefits will then be described in an appropriately tentative manner, as follows. Sleep problems and related issues in NH residents with dementia is a well-documented problem. This study implements an intervention which may improve the sleep experience for these residents. If the LOCK sleep program is found to be effective in enhancing clinical care and outcomes, the approach could be shared with other NHs and incorporated into general NH practice. The risks in this study for NH residents are considered minimal, but risk cannot be eliminated. The knowledge gained may result in improved quality of sleep, and ultimately improved quality of life for NH residents with dementia, but benefits cannot be guaranteed. Similarly the risks for NH staff are minimal, but risk cannot be eliminated, and the knowledge to be gained may result in a more effective and enjoyable workplace experience, but these benefits cannot be guaranteed.

Regarding alternatives to participation, the LOCK sleep program intervention described will be implemented NH-wide. The study uses an incomplete stepped-wedge cluster randomized controlled trial design in which each NH serves as its own control. There will therefore be no alternative treatments. NH residents and their legally authorized representatives will have the right to refuse participation in any portion of their care just as they would with usual NH care, including use of actigraphs and Fitbits. NH staff will have the right to refuse any interviews.

Informed Consent

Title	Consent Type	Attached Date
LAR Consent	Consent	10/16/2019
Waiver of written consent for NH staff	Waiver of Written Consent	10/16/2019
NH Staff Consent	Consent	11/26/2019

*** * * Assent * * ***

13 Assent

Complete this section if your study includes minors. An assent document should be used if participants are 6 to 18 years of age. The Assent Form Template provides guidelines for writing assent documents.

1) **Will minors be asked to give assent? If not, please justify.**

Note: For studies that require a discussion about reproductive risks, note that the conversation with the minor should take place separately from the parents. Also, if a minor will reach adulthood (18 in Missouri) during the course of the study, they will need to be asked to consent as an adult at that time to continue in the study.

*** * * HIPAA * * ***

14 HIPAA

Studies that receive or create protected health information (PHI) are subject to HIPAA regulations. PHI is health information with one or more personal identifiers. For more information see: http://www.ua.edu/research/index.html If you are working with UMC, then a separate IRB approval is required. This must be obtained prior to IRB submission and attached.

1) Will health information be accessed, received or collected?

No health information. HIPAA does not apply.

X Yes (continue to question 2).

2) Which personal identifiers will be accessed, received or collected?

No identifiers. I certify that no identifiers from the list below will be received or collected and linked to health information. HIPAA does not apply (skip remainder of page).

X Names

Social Security numbers

X Telephone numbers

Linkable code or any other unique identifying number (note this does not mean the unique code assigned by the Investigator(s) to code the research data)

All geographic subdivisions smaller than a State, including street address, city, county, precinct, zip code, and their equivalent geocodes, except for the initial three digits of a zip code, if, according to the current publicly available data from the Bureau of the Census: (1) The geographic unit formed by combining all zip codes with the same three initial digits contains more than 20,000 people; and (2) The initial three digits of a zip code for all such geographic units containing 20,000 or fewer people is changed to 000

X All elements of dates (except year) for dates directly related to an individual, including birth date, admission date, discharge date, date of death; and all ages over 89 and all elements of dates (including year) indicative of such age, except that such ages and elements may be aggregated into a single category of age 90 or older

Fax numbers

X Electronic mail addresses

Medical record numbers

Health plan beneficiary numbers

Account numbers

Certificate/license numbers

Vehicle identifiers and serial numbers, including license plate numbers

Device identifiers and serial numbers

Web Universal Resource Locations (URLs)

Internet Protocol (IP) address numbers

Biometric identifiers, including finger and voice prints

Full face photographic images and any comparable images

If you are receiving or collecting health information and at least one personal identifier, HIPAA applies to your study. Please continue to complete the sections, below.

3) Sources of Protected Health Information:

X Hospital/medical records for in or out patients

X Physician/clinic records

Laboratory, pathology and/or radiology results

Biological samples

X Interviews or questionnaires/health histories

Mental health records

Data previously collected for research purposes

Billing records

X Other Please describe:

Our secondary outcomes come from the MDS 3.0. Every Medicare-certified NH is required to complete
an MDS assessment for every resident at admission, quarterly, and at discharge, in addition to
whenever the resident's status changes.

4) If data will be shared outside the research team and the study involves PHI indicate how the research team will share the information. Contact the University of Alabama Privacy Officer for guidance on the proper procedures for sharing of protected health information. http://hipaa.ua.edu/

X Not applicable (continue to question 5).

Only linkable code that can link data to the identity of the participant. A code access agreement or business associate agreement may be needed when data are shared with other non-University of Alabama entities. If necessary, the agreement can be added and uploaded in item #5, below.

Limited identifiers: Zip codes, dates of birth, or other dates only. The study qualifies as a Limited Data Set. A data use agreement may be needed when data are shared with other non-AlaUniversity of Alabamatities. If necessary, the agreement can be added and uploaded in item #5, below.

With unlimited identifiers. The consent document and HIPAA Authorization form must describe how the information will be disclosed.

5) A HIPAA Authorization Form or Waiver of HIPAA Authorization is required for this study. Use the table below to add HIPAA Documents for your study. If you are accessing medical records, or other health records that include PHI, you must complete a waiver of HIPAA authorization.

HIPAA Documents

HIPAA Documents	Title	Attached Date
HIPAA Authorization	HiPAA Authorization	10/16/2019
Waiver of Authorization	Waiver HiPAA Authorization for Screening	

*** * * Attachments * * ***

15) Attachments

In this section, please upload additional documents associated with your protocol. Failure to attach files associated with the protocol may result in the protocol being returned to you.

Possible documents for this protocol could include:

Bibliography

Cooperating Institution's IRB Approval

Data Collection Sheet

Debriefing Script

Device Information/Documentation

Grant Proposal/Sub-Contract

Human Participants Training Certificate/Proof of Training

IND Application Letter

Information Sheet/Brochure

Interview/Focus Group Questions
Investigator's Brochure
Letter of Agreement/Cooperation
Package Insert
Patient Diary Form
Phone Script
Questionnaire/Survey
Recruitment Material (e.g., flyers, ads, e-mail text)
Recruitment Statement (if there is no waiver of written consent)
Scientific/PPC Review
Sponsor's Protocol
Sponsor's Protocol Amendment
Study Design Chart/Table
Waiver Request
Other files associated with the protocol (most standard formats accepted: pdf, jpg, tif, mp3, wmv, etc.)

To update or revise any attachments, please delete the existing attachment and upload the revised document to replace it.

Document Type	Document Name	Attached Date	Submitted Date
Questionnaire/Survey	IRB protocol appendices measures	10/15/2019	10/16/2019
Human Subjects Training Certificate/Proof of Training	11. Snow - CITI	10/15/2019	10/16/2019
Human Subjects Training Certificate/Proof of Training	9. Parmelee - CITI	10/15/2019	10/16/2019
Human Subjects Training Certificate/Proof of Training	5. Hartmann -CITI	10/15/2019	10/16/2019
Human Subjects Training Certificate/Proof of Training	10. Richards - CITI	10/15/2019	10/16/2019
Human Subjects Training Certificate/Proof of Training	4. Fry - CITI	10/15/2019	10/16/2019
Human Subjects Training Certificate/Proof of Training	8. Morgan - CITI	10/15/2019	10/16/2019
Other	Figure 1 LOCK Elements	10/15/2019	10/16/2019

Study Design Chart/Table	Figure 2 Example Step with Measurements	10/15/2019	10/16/2019
Other	Table 1 Outcome measurements and sources	10/15/2019	10/16/2019
Grant Proposal/Sub-Contract	Research Plan_Bibliography_NIA 1R61AG065619-01	10/15/2019	10/16/2019
Grant Proposal/Sub-Contract	NIH_Notice of Award_1R61AG065619-01	10/15/2019	10/16/2019
Other	All Investigator Biosketches_Bundled	10/15/2019	10/16/2019
Human Subjects Training Certificate/Proof of Training	1. Baier - CITI	10/15/2019	10/16/2019
Human Subjects Training Certificate/Proof of Training	2. Brady - CITI	10/15/2019	10/16/2019
Human Subjects Training Certificate/Proof of Training	3. Frank - CITI	10/15/2019	10/16/2019
Human Subjects Training Certificate/Proof of Training	6. McCreedy - CITI	10/15/2019	10/16/2019
Human Subjects Training Certificate/Proof of Training	7. McCullough - CITI	10/15/2019	10/16/2019
Other	Snow 19-024-ME 11-18-19 Notification	11/18/2019	11/18/2019
Other	B&F Consulting LOS Excerpt	11/26/2019	
Other	Biosketch CathieBrady_20190219	11/26/2019	
Other	Biosketch BarbaraFrank_20190218	11/26/2019	
Other	19-024-ME_response cover letter_11.26.19	11/26/2019	

----

* * * PI Obligations * * *

PI Obligations

By clicking the box below, you indicate that you accept responsibility for and will follow the

ethical guidelines.

1) Have you completed the annual Statement of Financial Interest (i.e., disclosure)? Y

NOTE: An annual disclosure must be completed by all faculty, staff, and students who are identified as senior/key personnel receiving federal funding for research. The disclosure can be completed online at https://www.formstack.com/forms/index.php?1338617-e6Kw9EILFS.

2) Have your financial interests changed significantly since you completed the annual disclosure form? N

According to the UA policy on conflict of interest, it is the PI's responsibility to inform co-investigators, staff, or students involved in the design, conduct, or reporting of federally sponsored research of their requirement to complete the Statement of Financial Interest.

X **I accept this responsibility.**

By submitting this form, the PRINCIPAL INVESTIGATOR certifies that he/she has read the UA policy on conflict of interest and has a current Statement of Financial Interest on file. In addition, the PI certifies that, to the best of his/her knowledge, no person working on this project at UA has a conflict of interest or, if a conflict of interest does exist, an appropriate management plan is in place.

X **The Principal Investigator has read and agrees to abide by the above obligations.**

The Department Chair has read and agrees to abide by the above obligations.

The Faculty Sponsor / Mentor has read and agrees to abide by the above obligations.

---

* * * Event History * * *

Event History

Date	Status	View Attachments	Letters
09/22/2019	NEW FORM CREATED		
10/16/2019	NEW FORM SUBMITTED	Y	
10/31/2019	NEW FORM PANEL ASSIGNED		
11/01/2019	NEW FORM REVIEWER(S) ASSIGNED		

Appendix H – Consent and HIPAA forms

THE UNIVERSITY OF ALABAMA®
Office of the Vice President for
Research & Economic Development
Office for Research Compliance

December 11, 2019

A. Lynn Snow, PhD
Professor
Department of Psychology
The University of Alabama
Box 870348

Re: IRB Protocol # 19-024-ME
"Enhancing Sleep Quality for Nursing Home Residents with Dementia: Pragmatic Trial of an Evidence-Based Frontline Huddling Program (Pilot PhaseR61)"

Dr. Snow:

The University of Alabama Medical Institutional Review Board has granted approval for your proposed research. You have also been granted the requested waiver of documentation of informed consent. Your application has been given full board approval according to 45 CFR part 46.

The approval for your application will lapse on December 4, 2020. If your research will continue beyond this date, please submit a continuing review to the IRB as required by University policy before the lapse. Please note, any modifications made in research design, methodology, or procedures must be submitted to and approved by the IRB before implementation. Please submit a final report form when the study is complete.

Please use reproductions of the IRB approved stamped consent form to obtain consent from your participants.

Good luck with your research.

Sincerely,

J. Grier Stewart, MD, FACP
Medical IRB Chair

Legally Authorized Representative Informed Consent

Please read this informed consent carefully before you decide to participate in the study.

Consent Form Key Information:

- This is a study of an intervention to improve sleep for nursing home residents with dementia
- The intervention focuses on teaching nursing home staff new ways of working within their teams to improve how they provide care to the person you represent to improve their sleep
- The person you represent will be asked to wear an actigraph (a wrist-watch like device) and a fit-bit (another wrist-watch like device) for 7 days on four separate occasions to measure their sleep quality
- We are also asking your permission for us to access the medical chart of the person you represent for information that would be relevant to our understanding of their sleep – this includes their diagnoses, functional abilities, cognitive abilities, mood experiences, pain experiences, and sleep experiences.
- We are also asking your permission for us to access staff ratings of the sleep and sleep-related experiences (like pain or agitation) of the person you represent during the study period
- The interventions the staff may provide to the person you represent as part of our training program are consistent with good clinical practice, and may include: decreasing light, volume, and resident interruptions at night; increasing meaningful activity during the day to promote better sleep at night; and modifying evening nutrition, hydration, and medication practices to promote uninterrupted sleep. Any interventions would be the final decision of the nursing home clinical team in collaboration with the physician – our study team would only be providing training recommendations and serving as advisors.

Purpose of the research study: The purpose of the study is to trial a nursing home staff training program that may help improve the sleep of nursing home residents with dementia. It is hoped this study will increase our understanding of how to implement and sustain nursing home quality improvement interventions. If the study is successful, the results will be used to guide the study team in making refinements to the sleep program so that the effectiveness of the sleep program can be thoroughly investigated in a subsequent large randomized controlled trial.

We are inviting the person you represent to participate in this study because they meet the following inclusion criteria for participation: they are >= 50 years old, they have an Alzheimer's diseas or related dementia diagnosis, and the nursing home staff think they may have a sleep problem that is likely to benefit from sleep intervention. They also do not have the following exclusion criteria: obstructive sleep apnea, bilateral resting tremor, nor paralysis in both arms.

What the person you represent will do in the study: If you agree for the person you represent to participate in this study, then the nursing home clinical team will assess the person's sleep quality and if their sleep is poor, the staff will work together to try a variety of evidence-based approaches to improve his or her sleep.

UNIVERSITY OF ALABAMA IRB
CONSENT FORM APPROVED: 12-11-19
EXPIRATION DATE: 12-4-20

If the team thinks the person you represent has sleep difficulty, they will measure the person's sleep quality using an actigraph and a fitbit. These are wrist-watch like devices that are worn on the wrist while the person sleeps. They give estimates of how well the person is sleeping and how long the person sleeps. The sleep measurement period using these devices would be 7 days, and would occur at four times over a 22 week period – at the beginning of the 22 weeks, at 8 weeks, at 15 weeks, and at the end of the 22 weeks.

The team would come together regularly to discuss the person you represent and to evaluate what adjustments to their care might help improve their sleep. The interventions the staff may provide to the person you represent as part of our training program are consistent with good clinical practice, and may include: decreasing light, volume, and resident interruptions at night; increasing meaningful activity during the day to promote better sleep at night; and modifying evening nutrition, hydration, and medication practices to promote uninterrupted sleep. Any interventions would be the final decision of the nursing home clinical team in collaboration with the physician – our study team would only be providing training recommendations and serving as advisors.

We also ask your permission for us to access the medical chart of the person you represent for information that would be relevant to our understanding of their sleep – this includes their diagnoses, functional abilities, cognitive abilities, mood experiences, pain experiences, and sleep experiences.

We also ask your permission for us to access staff ratings of the sleep and sleep-related experiences (like pain or agitation) of the person you represent during the study period

Time required: The person that you represent would participate in the study for 22 weeks.

Risks: There are minimal risks associated with participation in this study.

The risks for participants in this trial are minimal. There are no anticipated physical or economic risks associated with this study. Regarding psychological risks to NH residents, the LOCK sleep program including frontline huddling provides a sequence of steps for the process of assessing and responding to sleep problems. During the intervention period NH residents enrolled in the LOCK sleep program will receive actigraph and Fitbit assessment, and although our teams' experience with use of these devices in hundreds of people with dementia indicates that they are widely tolerated as comfortable, there is a small risk a resident may find the devices to be uncomfortable or otherwise distressing. As part of their intervention training, staff will be instructed on behavioral signs of distress. If a participant exhibits significant behavioral distress due to assessment, staff will be instructed on modifications and alternatives. If the distress does not subside, staff will be instructed to discontinue the assessment.

An unlikely but possible legal or social risk associated with participating in this study would be invasion of privacy in the remote chance that there is a loss or lack of protection of data containing personally identifiable information. The study team will use careful procedures to guard the security and confidentiality of all data, including only using insured and tracked shipping services to ship data from the nursing home to the study team (e.g., UPS or Fedex) and using a numeric codes instead of names on

UNIVERSITY OF ALABAMA IRB
CONSENT FORM APPROVED: 12-11-19
EXPIRATION DATE: 12-4-20

data forms, and keeping the code list in a locked and password-protected location at the researcher's office at the University of Alabama in a separate location from the data itself.

Finally, it is possible that you as a legally authorized representative or the person you represent may encounter the risk of feeling coerced to participate in this study. To minimize this type of risk, all informed consent contact with potential participants such as yourself come through the study staff only, rather than through NH staff, and your discussions with us are kept confidential. Please know that the treatment of the person you represent will not be affected in any way by your decision to allow or not allow their participation, now or on in the future.

Benefits: There are no direct benefits to you for participating in this research study. The study may help us understand how better and earlier detection and treatment of sleep problems. The study may direct result in nursing home staff providing increased attention to person's conditions, particularly their sleep. The intervention may help nursing home staff to improve their communication and teamwork.

Confidentiality - Data linked with identifying information: The information from the person you represent in the study will be handled confidentially. Their information will be assigned a code number. The list connecting their and your name to this code will be kept in a locked file separate from the rest of the data. When the study is completed, and the data have been analyzed, this list will be destroyed. Their name and your name will not be used in any report.

Confidentiality cannot be guaranteed: Because of the clinical nature of the data, the data from the person you represent will not be confidential from the nursing home clinical team. The information will be known to the nursing home clinical team who collects the data and uses it for the treatment of the person you represent. These data will be recorded in the medical record of the person you represent and will be accessible to the nursing home clinical team. However, we can guarantee that the data from the person you represent will be confidential outside of the nursing home. The research study team will safeguard the data the study team uses and no one who is not a part of the nursing home clinical team nor part of the research study team have access to the data.

Voluntary participation: The participation of the person you represent in this study is completely voluntary. The nursing home care of the person you represent will not be affected in any way by their participation or lack of participation in this study.

Right to withdraw from the study: You have the right to withdraw the person you represent from the study at any time without penalty.

How to withdraw from the study: If you want to withdraw from the study, "contact the researcher using the contact information on the last page of this form, or tell anyone who works with the study. You may also ask the nursing home staff to pass this information along to the study staff – in this case the study staff may contact you to verify your wishes. There is no penalty to you or the person you represent for withdrawing at any time. If you would like to withdraw after your materials have been submitted, please contact the researcher or a member of the study team to provide these instructions.

UNIVERSITY OF ALABAMA IRB
CONSENT FORM APPROVED: 12-11-19
EXPIRATION DATE: 12-4-20

Compensation/Reimbursement: You and the person you represent will receive no payment for participating in the study.

Using data beyond this study:

The researcher would like to make the information collected in this study available to other researchers after the study is completed. Your information will be stored, used and shared for future research studies, including but not limited to studies of how to improve care in nursing homes and how to understand the experiences of people who live and work in nursing homes. Researchers of future studies will not ask your permission for each new study. However, the information you provide will be combined with the information provided by others to create a large data set. Your name and other information that could potentially identify you will not be connected to the information shared with other researchers nor will they attempt to identify you.

If you have questions about the study or need to report a study related issue please contact, contact:
Name of Principal Investigator: A. Lynn Snow, PhD
Title: Professor
Department Name: Alabama Research Institute on Aging and Psychology Department
Telephone: 205-348-7518
Email address: LSNOW@ua.edu

If you have questions about your rights as a participant in a research study, would like to make suggestions or file complaints and concerns about the research study, please contact:
Ms. Tanta Myles, the University of Alabama Research Compliance Officer at (205)-348-8461 or toll-free at 1-877-820-3066. You may also ask questions, make suggestions, or file complaints and concerns through the IRB Outreach Website at http://ovpred.ua.edu/research-compliance/prco/. You may email the Office for Research Compliance at rscompliance@research.ua.edu.

Agreement:

☐ I agree to provide proxy consent on behalf of the person I represent (as listed below) to participate in the research study described above.

☐ I do not agree to provide proxy consent on behalf of the person I represent (as listed below) to participate in the research study described above.

UNIVERSITY OF ALABAMA IRB
CONSENT FORM APPROVED: 12-11-19
EXPIRATION DATE: 12-4-20

Signature of Legally Authorized Representative Date

Print Name of Legally Authorized Representative Date

Print Name of Research Participant (Nursing Home Resident)

Signature of Investigator or other Person Obtaining Consent Date

Print Name of Investigator or other Person Obtaining Consent

UNIVERSITY OF ALABAMA IRB
CONSENT FORM APPROVED: 12-11-19
EXPIRATION DATE: 12-4-20

Nursing Home Staff Informed Consent

Please read this informed consent carefully before you decide to participate in the study.

Consent Form Key Information:

- This is a study of an intervention to improve sleep for nursing home residents with dementia
- The intervention focuses on teaching nursing home staff new ways of working with their teams to improve how they provide care to nursing home residents with dementia to improve their sleep
- The interventions the staff may provide to the person you represent as part of our training program are consistent with good clinical practice, and may include: decreasing light, volume, and resident interruptions at night; increasing meaningful activity during the day to promote better sleep at night; and modifying evening nutrition, hydration, and medication practices to promote uninterrupted sleep. Any interventions would be the final decision of the nursing home clinical team in collaboration with the physician – our study team would only be providing training recommendations and serving as advisors.
- We are asking your permission for us to interview you to hear your experiences and opinions about the intervention and training.
- We are also asking your permission for us to record you during the interview so that we can transcribe this interview

Purpose of the research study: The purpose of the study is to trial a nursing home staff training program that may help improve the sleep of nursing home residents with dementia. It is hoped this study will increase our understanding of how to implement and sustain nursing home quality improvement interventions. If the study is successful, the results will be used to guide the study team in making refinements to the sleep program so that the effectiveness of the sleep program can be thoroughly investigated in a subsequent large randomized controlled trial.

We are inviting you to participate in this study because you work here at the nursing home.

What the person you represent will do in the study: If you agree to participate in this study, then we will interview you at a time and location of our mutual choosing, and will record the interview.

Time required: This interview will take approximately one hour. We would like to conduct a second follow-up interview with you at the end of the study (in a few months) but you are not obligated to participate in the second interview if you do not wish to do so.

Risks: There are minimal risks associated with participation in this study.

The risks for participants in this trial are minimal. An unlikely but possible legal or social risk associated with participating in this study would be invasion of privacy in the remote chance that there is a loss or lack of protection of data containing personally identifiable information. The study team will use careful procedures to guard the security and confidentiality of all data, including using a numeric code instead

UNIVERSITY OF ALABAMA IRB
CONSENT FORM APPROVED: 12-11-19
EXPIRATION DATE: 12-4-20

of your names on data forms and recordings, and keeping the code list in a locked and password-protected location at the researcher's office at the University of Alabama in a separate location from the data itself. We will also maintain confidentiality regarding your participation in this interview. We will not share information about your participation (or decision to not participate) with anyone else who works here in this nursing home nor for this nursing home corporation.

Finally, it is possible that you as a legally authorized representative or the person you represent may encounter the risk of feeling coerced to participate in this study. To minimize this type of risk, all informed consent contact with potential participants such as yourself come through the study staff only, rather than through NH staff or leadership, and your discussions with us are kept confidential. Please know that your treatment as an employee will not be affected in any way by your decision to participate or not participate, now or on in the future.

Benefits: There are no direct benefits to you for participating in this research study. The study may help us understand how better and earlier detection and treatment of sleep problems. The study may direct result in nursing home staff providing increased attention to person's conditions, particularly their sleep. The intervention may help nursing home staff to improve their communication and teamwork, which in turn may increase job satisfaction.

Confidentiality - Data linked with identifying information: The information from your interview will be handled confidentially. The recording, notes, and forms will all be assigned a code number. The list connecting their and your name to this code will be kept in a locked file separate from the rest of the data. Your interview audiorecording will be kept in a locked and password protected area accessible only to research staff. When the study is completed, and the data have been analyzed, the recording will be destroyed. Your name will not be used in any report.

Voluntary participation: Your participation is completely voluntary. Your employment will not be affected in any way by your participation or lack of participation in this study.

Right to withdraw from the study: You have the right to withdraw from the study at any time without penalty.

How to withdraw from the study: If you want to withdraw from the study, contact the researcher using the contact information on the last page of this form, or tell anyone who works with the study. There is no penalty to you for withdrawing at any time. If you would like to withdraw after your materials have been submitted, please contact the researcher or a member of the study team to provide these instructions.

Compensation/Reimbursement: You will receive no payment for participating in the study.

Using data beyond this study: The researcher would like to make the information collected in this study available to other researchers after the study is completed. Your information will be stored, used and shared for future research studies, including but not limited to studies of how to improve care in nursing homes and how to understand the experiences of people who live and work in nursing homes. Researchers of future studies will not ask your permission for each new study. However, the information

UNIVERSITY OF ALABAMA IRB
CONSENT FORM APPROVED: 12-11-19
EXPIRATION DATE: 12-4-20

you provide will be combined with the information provided by others to create a large data set. Your name and other information that could potentially identify you will not be connected to the information shared with other researchers nor will they attempt to identify you.

If you have questions about the study or need to report a study related issue please contact, contact:
Name of Principal Investigator: A. Lynn Snow, PhD
Title: Professor
Department Name: Alabama Research Institute on Aging and Psychology Department
Telephone: 205-348-7518
Email address: LSNOW@ua.edu

If you have questions about your rights as a participant in a research study, would like to make suggestions or file complaints and concerns about the research study, please contact:
Ms. Tanta Myles, the University of Alabama Research Compliance Officer at (205)-348-8461 or toll-free at 1-877-820-3066. You may also ask questions, make suggestions, or file complaints and concerns through the IRB Outreach Website at http://ovpred.ua.edu/research-compliance/prco/. You may email the Office for Research Compliance at rscompliance@research.ua.edu.

Agreement:

☐ I agree to provide consent to participate in the research study described above.

☐ I do not agree to provide consent to participate in the research study described above.

☐ I agree to audiotaping in the research study described above.

☐ I do not agree to video (audio, photograph) in the research study described above.

____
Signature of Research Participant Date

____
Print Name of Research Participant

____
Signature of Investigator or other Person Obtaining Consent Date

____
Print Name of Investigator or other Person Obtaining Consent

UNIVERSITY OF ALABAMA IRB
CONSENT FORM APPROVED: 12-11-19
EXPIRATION DATE: 12-4-20

THE UNIVERSITY
OF
ALABAMA

AAHRPP DOCUMENT # 114
UNIVERSITY MEDICAL CENTER
[hereinafter referred to as "UMC]

Title: Authorization For Use or Disclosure of Health Information

I hereby authorize the use or disclosure of my individually identifiable protected health information ("PHI") as described below. Unless explicitly excluded, this Authorization includes any information relating to drug and/or alcohol abuse/treatment, communications with psychiatrists or psychologists or records pertaining to sexually transmitted diseases, if they are a part of my medical record. I understand that this authorization is voluntary. Once this information has been disclosed, it may be subject to redisclosure and may no longer be protected by federal privacy regulations.

Patient name: ______________________________ **Chart Number:** ______________________________
Patient SSN: ______-________-_____________ **Patient DOB:** ______/______/________________

Persons/organizations providing the information:
____
____
____

Persons/organizations receiving the information:
____
____
____

Specific description of information (including date(s)): (more detailed description of information may be attached)
____
____
____

Release Information By: **Mail:( ) yes ( ) no** **Telephone: ( ) yes ( ) no** **Other: ( ) yes ( ) no**
Fax: ( ) yes ( ) no **Email: ( ) yes ( ) no**

Purpose of Use or Disclosure: (individual may indicate "at the request of the individual")
____
____

If for marketing, will UMC receive payment/benefit from the third party receiving the PHI? __Yes __No __N/A

Authorization Expiration Date or Event: ___________________________________
(NOTE: After this date or event has passed, this authorization to use/disclose will no longer be valid. Unless otherwise specified, an authorization will be valid for 6 months after the date it is signed. If authorization is for research purposes, the statement "end of the research study" or "none" or similar language will extend your permission beyond 6 months.)

The patient or the patient's representative must read and initial the following statements:

Initials: _______ I understand that I may revoke this Authorization at any time by notifying UMC Privacy Officer in writing, but if I do, it will not have any affect to the extent UMC took action in reliance on the Authorization.

Initials: _______ I understand that UMC may not condition the provision of treatment, payment, enrollment in a health plan, or eligibility for benefits on signing this Authorization, except under the following circumstances:

- participating in research projects can be conditioned on my signing an Authorization to use and disclose PHI in the research
- initial enrollment in health plans can be conditioned on signing an Authorization for the health plan to review PHI to make eligibility determinations
- furnishing healthcare services to me at the request of a third party can be conditioned on me signing an Authorization for disclosure of the PHI to the third party requesting the treatment.

Signature of patient or patient's representative: ___________________________________Date:________________

Printed Name of patient's representative: ____

Relationship to the patient/description of authority to act for patient: ______________________________

850 5th Avenue East Tuscaloosa, AL 35401 Ph: (205) 348-1252 Fax: (205) 348-2402
Attn: Medical Records Department

UA IRB Approved Document
Approval date: 12-11-19
Expiration date: 12-4-20

APPENDIX I - STUDY MEASURES AND INTERVIEWS

1: Clinical Global Impression of Change Rating

2: R61 Mid-implementation interview guide

3: R61 Post-implementation interview guide

4: R33 Post-implementation interview guide

5: R33 Sustainment interview guide

6: BIMS

7: STOP-BANG

Clinical Global Impression of Change Rating

Clinical Impression of Change from Baseline:

- ☐ Marked improvement
- ☐ Moderate improvement
- ☐ Minimal improvement
- ☐ No change
- ☐ Minimal worsening
- ☐ Moderate worsening
- ☐ Marked worsening

R61 Mid-implementation interview guide

Note: The CFIR implementation framework and the Relational Coordination conceptual framework guided the construction of this interview guide. This is a semi-structured guide and meant to be used flexibly, with follow-ups, open-ended questions, and requests for clarification. Questions may be asked in any order.

Ice breaker

- How long have you worked here?
- How long have you been working in nursing homes (NHs)?
- It would help me to understand a little bit about you and your role in the NH. Could you outline for me what your daily tasks and responsibilities are?

Intervention Characteristics (relative advantage of the intervention)

- Tell me what you know about frontline staff huddling to improve residents' sleep.
- How was frontline staff huddling introduced to you?
- Frontline staff huddling directly addresses the issue of sleep and sleep disruption for residents. Have you tackled this area of care before?
    - How much of an issue/impact do you think disrupted sleep has for residents?
- How does the frontline staff huddling compare to existing programs to address sleep and teamwork?
    - Advantages?
    - Disadvantages?
- Do you know who the champions are for this in your NH?
- Who trained you and other staff about frontline staff huddling? What did you think of that training?
- Does the frontline staff huddling to improve residents' sleep make sense to you?

Process

- Tell me how things are going with the frontline staff huddling intervention…
- At the beginning, was it clear what you were being asked to do?
- Have you participated in the frontline staff huddles? How is that going?
  - What was the huddle about?
  - How often did you huddle? (e.g. daily, weekly)
  - What did it look like?
  - How many people involved?
  - How long were the huddles?
  - How often do the huddles end with action plans?
  - How often do the huddles discuss the results of action plans from prior huddles (i.e., follow up on whether the action was effective or not)?
  - What did you like most about the huddle?
  - What worked well about the huddle?
  - What didn't work well?
  - How would you improve the huddle?
- If you had questions about frontline staff huddling, who could you see to ask questions?
  - Do staff feel as if there is NH leadership support for frontline staff huddling?
- How involved are your fellow staff in the huddling?
  - Do staff feel engaged? Please describe
  - If not, what do you think they need to be more engaged?
- What kinds of information do staff received about how they are doing in improving teamwork and improving resident sleep?
  - Is there follow through on the action plans you come up with in the huddles?
- If you do get feedback on how you are doing, what do you do with that information? What do staff and managers say or do about that performance information?
- How prepared do you feel to do the huddles?
  - What helped make you feel prepared?
  - If you don't feel prepared, why?
- How confident (capable) are you about your participation in the frontline staff huddles?
- How confident do you think your team/work colleagues feel about participating in the frontline staff huddles?
- Is there anything you think you need to feel more capable of doing the frontline staff huddles?
- Any suggestions for improving frontline staff huddling from the staff point of view?
  - Are there parts of it that you would want to change if you could?
  - What would those be and why?

Inner setting (networks and communications—maps to relational coordination, culture, implementation climate)

- What is it like to work here?
  - Is there a team culture? (please give an example)
  - What is the atmosphere like? (how do staff treat each other, how are residents treated and how do management treat staff)

    i. Are things really structured at your workplace—for example, is it a top down approach? Does leadership/management check-in with all levels of staff to see how things are going? How do things work here?
    ii. Do staff feel supported to make changes in
    iii. resident care or advance in their careers?
    iv. What happens when there are issues or problems?

- How does your supervisor communicate new information to you? (e.g., shift change report, hallway conversation, email, notice board)
    - Are there other ways you would like them to communicate new information to you?
- How do you communicate important information to your supervisor?
    - Are there other ways you would like to communicate to your supervisor?
- Who do you consider part of your team?
    - Do you feel like your team respects the work you do?
    - When problems arise how does your team work to solve the problem?
    - How do staff communicate with each other? What kinds of information is shared?
    - How has participating in frontline staff huddles changed things in your unit?
- In thinking about your work place, how open are people to new ideas?
    - What happens when someone has a suggestion for a change?
- What kinds of support and action have you seen from leaders and managers that support the frontline staff huddles?
    - Is there something that you would like to see and hear from leadership that you have not heard?
    - Has there been something particularly helpful that has happened that you could share?

Outer setting (peer pressure to implement)

- Have you heard about other nursing homes implementing frontline staff huddling?
- Have you heard other success stories about frontline staff huddles?
- To what extent does implementing frontline staff huddling provide an advantage for your residents and your NH as compared to other NHs?

Fitbit and actigraph

- You're using both Fitbits and actigraphs with your residents. Do you prefer one or the other?
- How do you use the data from the actigraphs, if at all?
- How do you use the data from the Fitbits?
- Thoughts on how helpful this data is for you and other staff?

Final questions

- Is there anything else you would like to tell me?
- Anything else you think I should know about the frontline staff huddles?
- Any concerns or thoughts you have?

R61 Post-intervention interview guide

Note: The CFIR implementation framework and the Relational Coordination conceptual framework guided the construction of this interview guide. This is a semi-structured guide and meant to be used flexibly, with follow-ups, open-ended questions, and requests for clarification. Questions may be asked in any order.

Ice breaker

- It would help me to understand a little bit about you and your role in the NH. We may have covered this before, but could you outline for me what your daily tasks and responsibilities are?

Intervention Characteristics (relative advantage of the intervention)

- How was frontline staff huddling introduced to you?
- frontline staff huddling was designed to directly address the issue of sleep and sleep disruption for residents.
    - How much of an issue/impact do you think disrupted sleep has on residents and do you think this has changed over the course of doing frontline staff huddles?
- How does frontline staff huddling compare to existing programs that addressed sleep and/or teamwork?
    - Advantages?
    - Disadvantages?

Process

- Tell me how things went with the frontline staff huddling…
- Have you participated in the frontline staff huddles? How did that go? Or is that still going?
    - What was the huddle about?
    - How often did you huddle? (e.g. daily, weekly)
    - What did it look like?
    - How many people were involved?
    - How long were the huddles?
    - How often did the huddles end with action plans?
    - How often did the huddles discuss the results of action plans from prior huddles (i.e., follow up on whether the action was effective or not)?
    - What did you like most about the huddle?
    - What worked well about the huddle?
    - What didn't work well?
    - How would you improve the huddle?
- What kinds of information did staff received about how they are doing in improving teamwork and improving resident sleep?
    - Is there follow through on the action plans you came up with in the huddles?
- If you had questions about frontline staff huddling who could you see to ask questions?
- Do staff feel as if there is NH leadership support for frontline staff huddles?

- Looking back on the intervention, how involved and engaged were staff in the frontline staff huddles? Can you give me some examples?
- If you did get feedback on how you were doing, what did you do with that information? What did staff and managers say or do about that performance information?
- What has changed/improved for frontline staff since participating in the frontline staff huddling?
- Looking back, did you feel prepared for the frontline staff huddles?
    - What helped make you feel prepared?
- How confident has frontline staff huddling made you feel about taking on problems or challenges?
- Have you learned any new skills from participating in frontline staff huddles?
- Any suggestions for improving the frontline staff huddles from the staff point of view?
    - Are there parts of it that you would want to change if you could?
    - What would those be and why?

Inner setting (networks and communications—maps to relational coordination, culture, implementation climate)

- Since the frontline staff huddles, has anything changed in your work environment and in your work atmosphere?
    - Any changes before the huddling versus after?
- Has teamwork improved?
    - Do you feel like your team respects the work you do?
    - When problems arise how does your team work to solve the problem?
    - How do staff communicate with each other? What kinds of information is shared?
    - How has participating in frontline staff huddling and doing huddles changed things in your unit?
- Has team communication changed in any way? Can you give an example?
- After participating in frontline staff huddling, tell me how does your supervisor communicate new information to you? (e.g. shift change report, hallway conversation, email, notice board)
    - How do you communicate important information to your supervisor?
    - Are there other ways you would like to communicate to your supervisor?
- What was challenging about doing frontline staff huddling?
    - Were there any sticking points we should know about?
    - Can you give me some examples?
- Over the time of the frontline staff huddles, what did you hear from leaders and managers about the frontline staff huddles?
    - Is there some kind of support that you would have liked to have seen?
    - Has there been something particularly helpful that has happened in terms of leadership/manager support that you could share?

Fitbit and actigraph

- You have used both Fitbits and actigraphs with your residents. Tell me about that experience…
- How did you use the data from the actigraphs, if at all?
- How did you use the data from the Fitbits? How valuable was the Fitbit data?

    - How did the Fitbit compare to the actigraph in terms of ease of use, meaningfulness of data, etc?
    - If you to choose which to use in your NH from now on, which would you choose?
- How easy or difficult was it to put 2 devices on residents instead of one? Would you recommend using both to other NHs in this study? Do you have any recommendations for other NHs on how to use both?
- Any other feedback that you have about the Fitbits and the actigraphs?
    - Any concerns we should know about?
    - How helpful was it having them?
    - Any other preference for one or the other that we should understand? And let us know why…

Sustainment

- Thinking about frontline staff huddling, do you think you will continue huddling? Are there other issues or problems that you think the huddle could be used to work on?
    - [If yes re continuing] What things are in place that will help your nursing home continue the frontline staff huddles? What hurdles do you think still exist and are there plans to overcome them? What do you see happening in the future?
    - [If no re continuing] What is missing that would support your nursing home continue the frontline staff huddles? What hurdles are still in place and what prevents overcoming them? What do you see happening in the future?

Final questions

- We will conduct phone interviews with busy NH staff like yourself in the next phase of this study. What worked best for you about this phone interview? What worked least well?
- Do you have any suggestions for the best way to do a phone interview with frontline staff?
    - In thinking about how your work day goes, when would the best time be to have a chat with someone about how the frontline staff huddling went?
    - What are the key questions that you felt were the most important to share with me as I tried to learn how things went?
- Is there anything else you would like to tell me? Anything else you think I should know about the frontline staff huddles? Any concerns or thoughts you have?

R33 Post-intervention interview guide

Note: The CFIR implementation framework and the Relational Coordination conceptual framework guided the construction of this interview guide. This is a semi-structured guide and meant to be used flexibly, with follow-ups, open-ended questions, and requests for clarification. Questions may be asked in any order.

Ice breaker

- How long have you worked here?
- How long have you been working in nursing homes (NH)?
- It would help me to understand a little bit about you and your role in the NH. Could you outline for me what your daily tasks and responsibilities are?

Intervention Characteristics (relative advantage of the intervention)

- How was frontline staff huddling introduced to you?
- Frontline staff huddling was designed to directly address the issue of sleep and sleep disruption for residents.
    - How much of an issue/impact do you think disrupted sleep has on residents and do you think this has changed over the course of doing frontline staff huddling?
- How does frontline staff huddling compare to existing programs that addressed sleep and/or teamwork?
    - Advantages?
    - Disadvantages?

Process

- Tell me how things went with the frontline staff huddling…
- Have you participated in the frontline staff huddles? How did that go? Or is that still going?
    - What was the huddle about?
    - How often did you huddle? (e.g. daily, weekly)
    - What did it look like?
    - How many people were involved?
    - How long were the huddles?
    - How often did the huddles end with action plans?
    - How often did the huddles discuss the results of action plans from prior huddles (i.e., follow up on whether the action was effective or not)?
    - What did you like most about the huddle?
    - What worked well about the huddle?
    - What didn't work well?
    - How would you improve the huddle?
- What kinds of information did staff received about how they are doing in improving teamwork and improving resident sleep?
    - Is there follow through on the action plans you came up with in the huddles?

- If you had questions about frontline staff huddling who could you see to ask questions?
- Do staff feel as if there is NH leadership support for frontline staff huddling?
- Looking back on the intervention, how involved and engaged were staff in the frontline staff huddling? Can you give me some examples?
- If you did get feedback on how you were doing, what did you do with that information? What did staff and managers say or do about that performance information?
- What has changed/improved for frontline staff since participating in the Frontline Staff Huddling?
- Looking back, did you feel prepared for doing frontline staff huddling?
- How confident has frontline staff huddling made you feel about taking on problems or challenges?
- Have you learned any new skills from participating in frontline staff huddling?
-
    - Are there parts of it that you would want to change if you could?
    - What would those be and why?

Inner setting (networks and communications—maps to relational coordination, culture, implementation climate)

- Since frontline staff huddling began, has anything changed in your work environment and in your work atmosphere?
    - Any changes before versus after?
- Has teamwork improved?
    - Do you feel like your team respects the work you do?
    - When problems arise how does your team work to solve the problem?
    - How do staff communicate with each other? What kinds of information is shared?
    - How has participating in frontline staff huddling and doing huddles changed things in your unit?
- Has team communication changed in any way? Can you give an example?
- After participating in frontline staff huddling, tell me how does your supervisor communicate new information to you? (e.g. email, staff meeting)
    - How do you communicate important information to your supervisor?
    - Are there other ways you would like to communicate to your supervisor?
- What was challenging about doing frontline staff huddling?
    - Were there any sticking points we should know about?
    - Can you give me some examples?
- Over the time you have been doing frontline staff huddles, what did you hear from leaders and managers about the frontline staff huddling?
    - Is there some kind of support that you would have liked to have seen?
    - Has there been something particularly helpful that has happened in terms of leadership/manager support that you could share?

Fitbit and actigraph [if R33 phase uses both]

- You have used both Fitbits and actigraphs with your residents. Tell me about that experience…
- How did you use the data from the actigraphs, if at all?

- How did you use the data from the Fitbits? How valuable was the Fitbit data?
    - How did the Fitbit compare to the actigraph in terms of ease of use, meaningfulness of data, etc?
    - If you to choose which to use in your NH from now on, which would you choose?
- How easy or difficult was it to put 2 devices on residents instead of one? Would you recommend using both to other NHs in this study? Do you have any recommendations for other NHs on how to use both?
- Any other feedback that you have about the Fitbits and the actigraphs?
    - Any concerns we should know about?
    - How helpful was it having them?
    - Any other preference for one or the other that we should understand? And let us know why…

Sustainment

- Thinking about the frontline staff huddles, do you think you will continue huddling? Are there other issues or problems that you think the huddle could be used to work on?
    - [If yes regarding continuing] What things are in place that will help your nursing home continue the frontline staff huddles? What hurdles do you think still exist and are there plans to overcome them? What do you see happening in the future?
    - [If no regarding continuing] What is missing that would support your nursing home continue the frontline staff huddles? What hurdles are still in place and what prevents overcoming them? What do you see happening in the future?

Final Question

- Is there anything else you would like to tell me? Anything else you think I should know about the frontline staff huddles? Any concerns or thoughts you have?

R33 Sustainment interview guide

Note: The CFIR implementation framework and the Relational Coordination conceptual framework guided the construction of this interview guide. This is a semi-structured guide and meant to be used flexibly, with follow-ups, open-ended questions, and requests for clarification. Questions may be asked in any order.

Ice breaker

- Before we get going, it would help me to understand a little bit about you and your role in the nursing home (NH). You may have done this in an earlier interview, but could you briefly outline for me what your daily tasks and responsibilities are?

Intervention Characteristics (relative advantage of the intervention)

- Looking back at the frontline staff huddling intervention, do you think it was successful in addressing the issue of sleep and sleep disruption for residents?
- Thinking about other quality improvement and change initiatives you may have participated in…how does this intervention compare to existing programs that addressed sleep? What about teamwork?
    - Advantages?
    - Disadvantages?

Process

- Tell me, is your nursing home/unit/neighborhood using frontline staff huddling?
    - If no, why not?
        - i. What challenges are happening on your unit that you think is preventing huddling from happening?
        - ii. Do you miss the huddling?
        - iii. What is happening for residents and sleep now that huddling has decreased or is not happening?
        - iv. How do you feel about the huddles stopping?
        - v. What do other staff feel about the decrease or stopping of the huddling?
        - vi. Has anything else changed that we should know about it?

    - If yes, please tell me more about what you are doing?
        - i. How often do you huddle?
        - ii. What is the huddle about?
        - iii. What does a typical huddle look like?
        - iv. How long is a typical huddle?
        - v. What kind of action plans do huddles come up with?
        - vi. How are action plans followed up on? Who does this?
        - vii. Are you still following the original structure of the huddles?
            1. For example, are the same people in the same roles involved? Do huddles end with action plans? Do huddles discuss the results pf previous action plans?
        - viii. Have you improved or changed the huddles in any way?

ix. How confident has the frontline staff huddling intervention made you feel about taking on problems or challenges?
x. Have you learned any new skills from participating in the frontline staff huddling intervention? How are you applying these new skills currently?
xi. Why are you still doing huddles? What do they accomplish for residents? For frontline staff?

- If actigraph us is not addressed in the answers to the above questions, are you still using the actigraph device we left you?
  - If yes:
    i. How are you using it? How often?
    ii. How are you using the data?
    iii. What changes have you implemented as a result?
    iv. What do staff like best about using the actigraph? Like least?
    v. Anything else you'd like to add about the actigraph?
- Looking back on to when you began using the frontline staff huddles, how involved and engaged were staff? Can you give me some examples? How does that differ from now?

Inner setting (networks and communications—maps to relational coordination, culture, implementation climate)

- If they are still huddling: Looking back over your history of frontline staff huddling, can you describe what teamwork is currently like for you?
  - How did teamwork change over time?
  - Did team communication change over time?
  - Were there times when teamwork led to successful change?
  - Did any change get held up because teamwork was not working? What happen then?
  - Please discuss any changes you noticed that happened in regard to teamwork before huddling versus after.
- If they are no longer huddling: What is teamwork is currently like for you?
  - How did teamwork change over time, from the time when you were doing the huddling to now?
  - Did team communication change over time?
  - Were there times when teamwork led to successful change?
  - Did any change get held up because teamwork was not working? What happen then?
  - Please discuss any changes you noticed that happened in regard to teamwork when you were huddling versus after you stopped.
- Do you feel like your team respects the work you do?
- When problems arise how does your team work to solve the problem?
- How do staff communicate with each other? What kinds of information is shared?
- How has participating in frontline staff huddling and doing huddles changed things in your unit?
- If they are still huddling: Is there anything still challenging about doing huddling?
  - Can you give me some examples?
- What did you hear from leaders and managers about the frontline staff huddling intervention? Has that changed over time?

Mechanics of Sustainment

- If they are still huddling: Changes come and changes go; all staff know this. Can you reflect on how you think the frontline staff huddling intervention really took hold at your site? How did this behavior and practice change come about?
    - What happened that you think helped huddling at your site?
    - What happened that didn't help and how did you overcome those things?
    - What were the leadership/management actions that supported the change?
    - What work did you and other staff do that helped the huddling intervention succeed?
    - What lessons have you learned from this experience?
- If they are no longer huddling: Changes come and changes go; all staff know this. Can you reflect on how you think the frontline staff huddling intervention didn't take hold at your site?
    - What happened that you think helped huddling at your site?
    - What happened that didn't help? Did you try to overcome these things? What happened?
    - What were the leadership/management actions that supported or didn't support the change?
    - What work did you and other staff try to do to help the huddling intervention succeed?
    - What lessons have you learned from this experience?
- Thinking into the future for a minute, do you think you will continue huddling/start huddling again?
    - [If yes regarding continuing] What things are in place that will help your nursing home continue the frontline staff huddles? What hurdles do you think still exist and are there plans to overcome them? What do you see happening in the future?
    - [If no regarding continuing] What is missing that would support your nursing home continue the frontline staff huddles? What hurdles are still in place and what prevents overcoming them? What do you see happening in the future?
- Are there other issues or problems that you think the huddle could be used to work on?

Final Question

- Is there anything else you would like to tell me? Anything else you think I should know about huddling? Any concerns or thoughts you have?

Resident Name ____________________ Identification # ____________ Date ______________

Brief Interview for Mental Status (BIMS)

Repetition of Three Words

Ask resident: *"I am going to say three words for you to remember. Please repeat the words after I have said all three. The words are:* ***sock, blue*** *and* ***bed****. Now tell me the three words."*

Number of words repeated after first attempt:

☐ **0. None** ☐ **1. One** ☐ **2. Two** ☐ **3. Three**

After the resident's first attempt, repeat the words using cues *("sock, something to wear; blue, a color; bed, a piece of furniture")*. You may repeat the words up to two more times.

Temporal Orientation (orientation to month, year and day)

Ask resident: *"Please tell me what year it is right now."*

Able to report correct year

☐ **0. Missed by > 5 years**, or no answer
☐ **1. Missed by 2-5 years**
☐ **2. Missed by 1 year**
☐ **3. Correct**

Ask resident: *"What month are we in right now?"*

Able to report correct month

☐ **0. Missed by > 1 month**, or no answer
☐ **1. Missed by 6 days to one month**
☐ **2. Accurate within 5 days**

Ask resident: *"What day of the week is today?"*

Able to report correct day of the week

☐ **0. Incorrect**, or no answer
☐ **1. Correct**

Recall

Ask resident: *"Let's go back to the earlier question. What were the three words that I asked you to repeat?"*
If unable to remember a word, give cue (*"something to wear," "a color," "a piece of furniture")* for that word.

**Able to recall "sock"**	☐ **0. No - could not recall**	☐ **1. Yes, after cueing ("something to wear")**	☐ **2. Yes, no cue required**
**Able to recall "blue"**	☐ **0. No - could not recall**	☐ **1. Yes, after cueing ("a color")**	☐ **2. Yes, no cue required**
**Able to recall "bed"**	☐ **0. No - could not recall**	☐ **1. Yes, after cueing ("a piece of furniture")**	☐ **2. Yes, no cue required**

Summary Score

Add scores for each question and fill in total score (00-15).
Enter 99 if the resident was unable to complete the interview. __________

Abbreviated Instructions for Conducting the BIMS

Intent: To determine the individual's attention, orientation and ability to register and recall new information.

Please note: For more in-depth instructions for completing the BIMS, please refer to Chapter 3: MDS Items Section C: Cognitive Patterns.

SHOULD THE BRIEF INTERVIEW FOR MENTAL STATUS BE CONDUCTED?

- The interview should be attempted if the individual is at least sometimes understood verbally or in writing, and if an interpreter is needed and one is available.
- The interview should not be attempted if the individual is rarely/never understood or an interpreter is needed but not available.

BASIC INTERVIEW INSTRUCTIONS FOR BIMS:

1. Interview any individual not excluded as indicated above.
2. Conduct the interview in a private setting if at all possible.
3. Be sure the individual can hear you. An individual with a hearing impairment should be tested using their usual communication devices/techniques as applicable.
4. Sit so that the individual can see your face.
5. Give an introduction.
   Suggested language: *"I would like to ask you some questions. We ask everyone these same questions. This will help us to provide you with better recommendations. Some of the questions may seem very easy, while others may be more difficult."*
6. If the individual expresses concern that you are testing his or her memory, he or she may be more comfortable if you reply: *"We ask these questions of everyone so we can make sure that we can meet your needs."*

Coding Tips:

- Nonsensical responses should be coded as zero.[1] A nonsensical response is any response that is unrelated, incomprehensible, or incoherent; and is not informative with respect to the item being rated.
- Refusal to answer a specific question is coded as "0."

Use Code 99 if:

- the individual chooses not to participate, or
- 4 or more items were coded 0 because the individual chose not to answer or gave a nonsensical response.

[1] Nonsensical response means any response that is unrelated, incomprehensible, or incoherent; it is not informative with respect to the item being rated.

Repetition of Three Words

This section determines if the individual is able to actively engage in a verbal interaction. Inability to repeat three words on the first attempt may indicate a hearing impairment, a language barrier or inattention.

Interview Instructions:

1. Say to the individual; *"I am going to say three words for you to remember. Please repeat the words after I have said all three."*
2. Immediately after presenting the three words, say to the individual: *"Now tell me the three words."*

**Repetition of Three Words**
Ask the individual: *"I am going to say three words for you to remember. Please repeat the words after I have said all three. The words are: sock, blue and bed. Now tell me the three words."*<br>**Number of words repeated after first attempt**<br>**0. None**<br>**1. One**<br>**2. Two**<br>**3. Three**<br>After the individual's first attempt, repeat the words using cues *("sock, something to wear; blue a color; bed, a piece of furniture")*. You may repeat the words up to two more times.

Coding Instructions:

1. Record the maximum number of words that the individual correctly repeated on the first attempt. This will be any number between 0 and 3.
2. The words may be recalled in any order and in any context.

Important: After scoring, and in preparation for **Recall,** repeat the three words, this time using category cues: "*sock, something to wear; blue, a color; bed; a piece of furniture*."[2] You may repeat these words and the corresponding cues up to two more times.

[2] Category cue means a phrase that puts a word in context to help with learning and to serve as a hint that helps prompt the individual. For example, the category cue for sock is "something to wear."

Temporal Orientation (Orientation to Year, Month and Day)

Temporal orientation means the ability to place oneself in correct time.[3] For the BIMS, it is the ability to indicate the correct date in current surroundings.

Interview Instructions:

1. Ask the individual each of the 3 questions separately.
2. Allow the individual up to 30 seconds for each answer and do not provide clues.
3. If the individual specifically asks for clues (e.g. *"is it bingo day?"*) respond by saying, *"I need to know if you can answer this question without any help from me."*
4. In some cases, you may need to write the individual's response in the margin and go back later to count how many years, months or days were missed. Do your best to keep focused on the interaction with the individual, not adding or subtracting.

**Temporal Orientation (orientation to year, month, and day)**
Ask the individual: *"Please tell me what year it is right now."*<br>**A. Able to report correct year**<br>**0. Missed by more than 5 years or no answer.**<br>**1. Missed by 2-5 years**<br>**2. Missed by 1 year**<br>**3. Correct**
Ask the individual: *"What month are we in right now?"*<br>**B. Able to report correct month**<br>**0. Missed by more than 1 month or no answer.**<br>**1. Missed by 6 days to 1 month**<br>**2. Accurate within 5 days.**
Ask the individual: *"What day of the week is today?*<br>**C. Able to report correct day of the week**<br>**0. Incorrect or no answer**<br>**2. Correct**

Coding Instructions:

Code as indicated in the corresponding boxes.

[3] Temporal orientation means the ability to place oneself in correct time. For the BIMS it is the ability to indicate the correct date in current surroundings.

Recall

Individuals with cognitive impairment can be helped to recall if provided clues. Providing memory cues can help maximize the individual's function and decrease frustrations for those individuals who respond.

Interview Instructions:

1. Ask the individual the following: *"Let's get back to an earlier question. What were those three words that I asked you to repeat?"*
2. Allow up to 5 seconds for spontaneous recall of each word.
3. For any word that is not correctly recalled after 5 seconds, provide a category cue (*something to wear, a color, a piece of furniture*). Give a cue for each word separately.
4. Category cues should be used only after the individual is unable to recall one or more of the three words.
5. Allow up to 5 seconds after category cueing for each missed word to be recalled.

**Recall**
Ask individual: *"Let's go back to an earlier question: What were those three words that I asked you to repeat?"* If unable to remember a word, give cue (*something to wear; a color; a piece of furniture*) for that word.<br>**A. Able to recall "sock"**<br>0. **No** – could not recall<br>1. **Yes, after cueing** ("*something to wear*")<br>**2. Yes, no cue required**
**B. Able to recall "blue"**<br>0. **No** – could not recall<br>1. **Yes, after cueing** ("*a color*")<br>**2. Yes, no cue required**
**C. Able to recall "bed"**<br>0. **No** – could not recall<br>1. **Yes, after cueing** ("*a piece of furniture*")<br>**2. Yes, no cue required**

Coding Instructions:

1. Code as indicated in the corresponding boxes.
2. If on the first try (without cueing), the individual names multiple items in a category, one of which is correct, they should be coded as correct for that item
3. If however, the interviewer gives the individual the cue and the individual then names multiple items in that category, the item is coded as *"could not recall,"* even if the correct item was in the list.

TOTAL SCORE

Enter the total score as a two-digit number. The total possible BIMS score ranges from 00 to 15.

13 – 15: cognitively intact
08 - 12: moderately impaired
00 – 07: severe impairment

Updated STOP-Bang Questionnaire

Snoring?

Yes ◯ No ◯

Do you **Snore Loudly** (loud enough to be heard through closed doors or your bed-partner elbows you for snoring at night)?

Tired?

Yes ◯ No ◯

Do you often feel **Tired, Fatigued, or Sleepy** during the daytime (such as falling asleep during driving or talking to someone)?

Observed?

Yes ◯ No ◯

Has anyone **Observed** you **Stop Breathing** or **Choking/Gasping** during your sleep?

Pressure?

Yes ◯ No ◯

Do you have or are being treated for **High Blood Pressure**?

Yes ◯ No ◯

Body Mass Index more than 35 kg/m^2?

Yes ◯ No ◯

Age older than 50 year old?

Neck size large? (Measured around Adams apple)

Yes ◯ No ◯

For male, is your shirt collar 17 inches/43 cm or larger?
For female, is your shirt collar 16 inches/41 cm or larger?

Yes ◯ No ◯

Gender = Male?

Scoring Criteria:

For general population
Low risk of OSA: Yes to 0-2 questions
Intermediate risk of OSA: Yes to 3-4 questions
High risk of OSA: Yes to 5-8 questions
or Yes to 2 or more of 4 STOP questions + male gender
or Yes to 2 or more of 4 STOP questions + BMI > 35 kg/m^2
or Yes to 2 or more of 4 STOP questions + neck circumference

(17”/43cm in male, 16”/41cm in female)

Proprietary to University Health Network. www.stopbang.ca
Modified from: Chung F et al. Anesthesiology 2008; 108:812-21; Chung F et al. Br J Anaesth 2012, 108:768–75; Chung F et al. J Clin Sleep Med 2014;10:951-8.
