# Supplementary material for: Enhancing sleep quality for nursing home residents with dementia: a pragmatic randomized controlled trial of an evidence-based frontline huddling program
Source: BMC Geriatr. 2021 Apr 27;21:281. doi: 10.1186/s12877-021-02189-8 (PMC8076882; doi:10.1186/s12877-021-02189-8)
Supplement: Supplementary file 2 — Additional file 2. Data Resource and Sharing Plan. [file 12877_2021_2189_MOESM2_ESM.docx]

Data and Resource Sharing Plan

Enhancing Sleep Quality for Nursing Home Residents with Dementia: Pragmatic Trial of an Evidence-Based Frontline Huddling Program

1R61AG065619-01

Sharing of data generated by this project is an essential part of our proposed activities and will be carried out in several different ways. We will make our results and data available both to the community of scientists interested in dementia care, sleep improvement, and/or nursing home quality of care to avoid unintentional duplication of research and assure that subsequent research builds upon the knowledge generated by this project. Conversely, we would welcome collaboration with and the opportunity to support others who could make use of the protocols developed in this project.

Our data and resource sharing plan includes the following:

**What data that will be shared:**

We will share de-identified participant data by depositing data files and associated code books and analysis files with the data repository dataverse (dataverse.org). We will follow the University of Alabama IRB guidance and approval for ensuring that our data is appropriately de-identified before posting to dataverse. Our IRB-approved informed consent and HIPPAA forms seek participant permission for broad inclusion of participant data for these data sharing and secondary analysis purposes. We do not have authority to share Minimum Data Set (MDS) data, so we cannot deposit these data directly. However, we will share our analysis files and instructions on how to work with the federal owners of these data to request access; in this way other researchers will be able to replicate our steps in constructing the MDS data sets once they obtain the data from the federal owners (who do provide the data to the public via a standardized credentialed request process for a fee). Submitted data will confirm with relevant data and terminology standards. We will follow the FAIR (findable, accessible, interoperable, and re-usable) data principles in preparing the data and data documentation (i.e., metadata) for use through the dataverse repository.

**Where the data will be available:**

We agree to deposit and maintain the data and associated files (as described above) at dataverse. The dataverse repository has data access policies and procedures that are consistent with NIH data sharing policies.

**When the data will be shared:**

We agree to deposit data in the dataverse repository as soon as possible, and will ensure that the data and reference resources associated with a manuscript will be made available no later than the on-line publication date for that manuscript.

**How will researchers locate and access the data:**

We agree that we will identify where the data will be available and how to access the data in any publications and presentations that we author or co-author about these data, as well as acknowledge the repository and funding source in any publications and presentations. As we will be using dataverse, this repository has policies and procedures in place that will provide data access to qualified researchers, fully consistent with NIH data sharing policies and applicable laws and regulations.

Our results dissemination plan includes the following:

Dr. Snow, as the PI and the responsible party, will ensure this trial’s compliance with NIH’s most current Policy on Dissemination of NIH-Funded Clinical Trial Information.

1. This clinical trial will be registered in ClinicalTrials.gov and we will ensure that the trial’s summary results are also submitted thereto for public posting. We will register the trial no later than 21 calendar days after the enrollment of the first participant and will submit results information no later than one year after the trial's primary completion date.

2. We have ensured this trial’s informed consent documents include a specific statement relating to posting of clinical trial information at ClinicalTrials.gov.

3. We affirm that the University of Alabama has a policy to ensure that clinical trials registration and results reporting occur in compliance with policy requirements. Specifically, she has personally communicated with Carpantato Myles, MSM, CIM, CIP, Director and Research Compliance Officer for the UA Office of Research Compliance regarding the policy requirements, who states, “the Office of Research Compliance offers support, and assistance to University of Alabama faculty and staff with registration and reporting of clinical research trials in ClinicalTrials.gov. The office also serves as the lead administrator of the ClinicalTrials.gov Protocol Registration System, which facilitates the provision of account administration, technical advice, consulting, problem solving, and compliance monitoring services.”

4. We will post all publications and presentation materials to the University of Alabama’s institutional repository (IR; <http://ovpred.ua.edu/research-compliance/nih-public-access-policy/>). We will abide by the NIH public access policy (including submitting a copy of each manuscript to PubMed Central; <http://ovpred.ua.edu/research-compliance/nih-public-access-policy/>).
